# Supplementary material for: Progress toward Health System Readiness for Genome-Based Testing in Canada
Source: Curr Oncol. 2023 Jun 1;30(6):5379–94. doi: 10.3390/curroncol30060408 (PMC10296918; doi:10.3390/curroncol30060408)
Supplement: Supplementary file 1 [file curroncol-30-00408-s001.zip › curroncol-2406432-supplementary.pdf]

Towards the routine use of genome-based testing in Canada's largest regions:

A State of Readiness Progress Report

May, 2023

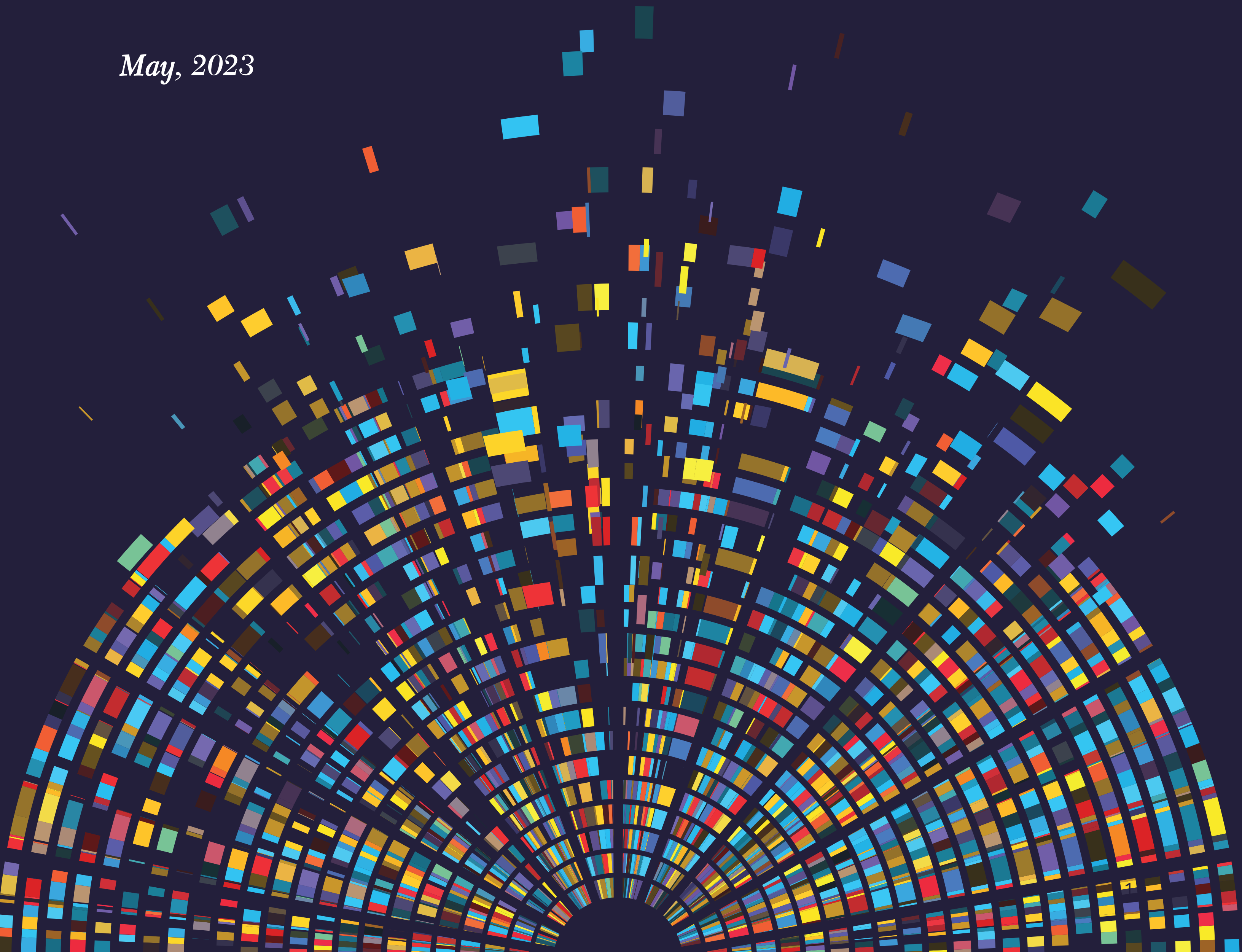

Acknowledgements

I would like to thank the numerous people who made this report possible. These include my medical librarian specialist Dagmara Chojecki, as well as my mentors and colleagues Terry Sullivan, Philip Jacobs, and Susan Rothery.

I am thankful for excellent engagement with local laboratory leaders and health system administrators who have the difficult task of trying to improve the system and policies surrounding this difficult issue. These include Craig Ivany, Michael Mengel, Denis Ouellet, François Sanschagrin, Jean Latreille, Guy Rouleau, Michael Carter, Chris Simpson, Anna Greenberg, Harriet Feilotter, Stephen Yip, and Raymond Kim. I am also thankful for advice and insights from international experts Lotte Steuten, Vivek Muthu, David Thomas, Daryl Spinner, as well as national experts Christopher McCabe, Arminee Kazanjian, and Christine Williams. I would finally like to thank Eva Villalba, who provided excellent insights on the patient journey and what improving testing services will mean for patients.

I am very thankful for Erin Milling, the Graphic Designer who provided support for formatting, layout and graphics of the report and was able to bring its findings and insights to life. I am also thankful to Hannah Casey, along with the Santis Health Communications Team who provided additional support, particularly for the executive summary and readiness grade infographics.

Finally, I am especially thankful to those individuals working in the life sciences sector who provided support and direction for this research and were able to convey the experiences of those innovating outside of health systems to improve the health and welfare of Canadians. This includes Larry Arshoff, Susan Baker, Stephen Burke, Oliver Johnston, Lisa Maslanka, Robyn Saccon, Jeffrey Smith, Nelson Ho, and Laura Thorpe. A very special thanks to Dana Corsen and Nadia Yee who led these efforts.

Additional thanks to regional representatives Michael Gingras, Jo-Ann Stuart Chatterley, Derek Badger, Aleina Spiegelman, Matthew Beer, Gurvinder Kenth, Laura Fitzgerald, Keith Gilchrist, Emanuela deFranco, John Brenton, Agnes Xenopolous, Shawn White, Brenda Fisher, Vivian Ng, Julie Gauthier, Manon Jobin, Isabelle Gagnon, Alain Couture, Sylvain Baril, Nathalie Brazeau, and Antonin Caron.

Citation:

Husereau D, Villalba E, Muthu V, Mengel M, Ivany C, Steuten L, Spinner DS, Sheffield B, Yip S, Thomas, DM, Jacobs, P, Sullivan T. etc. Towards the routine use of genome-based testing in Canada's largest regions: A State of Readiness Progress Report. 2023. 74 p. ISBN 978-1-7389568-0-7

Contributions

D.H conceived and designed all Chapters of the report, conducted the literature review and semi-structured interviews, and analysed content. D.H is responsible for the funding acquisition and led the writing of the original draft. L.S., V.M., D.M.T., D.S.S., C.I., M.M., B.S., S.Y., P.J. and T.S. contributed to the conceptualization, methodology, and writing—review and editing of Chapter 3 - the list of conditions. DH and EV conceived and designed sections related to patient impact and EV led the original draft writing. All authors approved the final version of their respective sections of the report . D.H. is the guarantor of this work. The conclusions of the authors were not contingent on the sponsor's approval or censorship of the manuscript. The conclusions are the authors and do not reflect the views of their affiliated organizations. All authors have read the report and agreed to the published versions of their relevant sections .

Inquiry:

Please direct any inquiries about this report to Don Husereau, Adjunct Professor, School of Epidemiology and Public Health, University of Ottawa : dhuserea@uottawa.ca

Funding:

Research support was provided equally to Don Husereau's company, 9363980 Canada Inc. by the following companies: Amgen Canada Inc., AstraZeneca Canada, Eli Lilly Canada Inc., GlaxoSmithKline Inc. (GSK Canada), Janssen Inc./J&J, Pfizer Canada ULC, Thermo Fisher Scientific Inc., and Roche Canada.

All of the views and interpretations in the report, including the readiness grades assigned, are my own and do not necessarily represent the official position of any of the individual participants and organizations who partici-pated in this work.

9363980 Canada Inc.
879 Winnington Ave
Ottawa, ON K2B 5C4
Tel: 613-299-4379
ISBN: 978-1-7389568-0-7

May , 2023

Executive Summary

Towards the routine use of genome-based testing in Canada's largest regions:

A State of Readiness Progress Report

Why genome-based testing?

High quality care that most benefits patients requires information from testing. Increasingly, laboratory-based biomarkers that measure the expression, function and regulation of genes and gene products are being used for this purpose. These genome-based tests have already become commonplace in oncology and there is expected to be an exponential growth in new tests and test approaches across a number of therapeutic areas in coming years — an era of genomic medicine.

A rapid increase in available innovation and technologic platforms will also lead to an increase in the complexity of implementing genome-based tests and how testing services can be delivered effectively and efficiently. As the structure, remit and organization of healthcare systems (and the laboratory functions within them) vary, there are likely to be no one-size-fits-all solution; however , some necessary conditions will be required to manage these technologies in a way that benefits patients and is sustainable.

Unlike traditional healthcare technologies, genome-based testing also provides research opportunities beyond healthcare decision making, and can lead to discoveries about the nature of disease or effectiveness of current and future therapies. They will also serve as an important part of implementing future cell and gene therapies and in the diagnosis and monitoring of genetic and rare disease.

What is the impact of system readiness?

Genomic medicine has already improved patient health outcomes through predicting who may benefit (or not be harmed) from therapy, and helping clinical decisions through better prognosis and diagnosis of disease.

However, genomic medicine harbors a number of additional benefits including:

- **Better patient and care provider experiences** – Reducing the need for referrals and other diagnostic tests, and improving time to diagnosis. Improving the state of readiness across Canada will lead to equitable care and access.
- **Better science and economic growth** – Aiding scientific discovery and clinical trial enrollment, creating commercial and investment opportunities as well as future-proofing Canada's healthcare workforce.
- **Healthcare efficiency** – Genomic medicine creates opportunities to reduce healthcare costs while creating the necessary infrastructure for delivering 21st century care.
- **Equitable access to care** – Readiness at a provincial level ensures equitable access to care across Canada and within provinces, including between academic and community settings. Standards for readiness also create opportunities for collaboration across provinces.

Summary

- Genomic medicine harbors the real potential to improve the health and healthcare journey of patients, care provider experiences, and improve health system efficiency – even reducing health care costs. There is expected to be an exponential growth in medically necessary new genome-based tests and test approaches in coming years.
    - Unlike traditional healthcare technology, genome-based testing can also create scientific research and commercial opportunities beyond healthcare decision-making.
- The State of Readiness Progress Report uses a purposive sample of 5 healthcare regions in Canada: Ontario, Quebec, British Columbia, Alberta, and Nova Scotia, representing >85% of the Canadian population.
    - While Canada has made some progress, it is far from ready. There are still significant gaps that need to be addressed. These gaps vary by province and include: better informatics/linked data systems; timely, fair and equitable test review processes; navigational and educational supports for care providers; timely and adequate financing for test services; and creating better opportunities for innovation through genomic medicine.
- Further research to fill these gaps should engage patients, care providers, and health systems.
    - Key priorities include implementation research to support innovators, linking research data to health data, and exploring the ethical legal and social implications of testing, particularly in high priorities such as rare disease.
- Further work must also be done to improve patient journeys from diagnosis of disease to receiving timely and high quality care that can benefit their families while advancing our scientific understanding of human health; creating better opportunities for genomic innovation is a clear means of improving the health and welfare of Canadians.

What Does Readiness Mean for Patients?

- **Improving health.** Genome-based testing enables more accurate and timely diagnosis or the use of targeted treatments which can be more effective and less harmful to patients. It may also help patients and care providers better understand the future of their disease, and help patients and their families make more informed decisions.
- **Improving care experiences for patients and their families.** Knowing what genetic mutations are responsible for their loved one's disease may be potentially lifesaving for both patients and family members and could also allow for earlier (and less costly) interventions.
- **Fair treatment.** Patients are concerned that access to valuable testing may be determined by where they live or how well-informed care providers are about the availability or health impacts of testing. Readiness for testing means patients can receive the best possible care, regardless of where they live.
- **Time and support.** Patients, especially those with rare conditions and poor prognoses, don't have time to waste. Genomic medicine can expedite their time to diagnosis and treatment. A ready health system can also offer necessary counselling and psychosocial support for patients and their loved ones to better understand their illness.

Is Canada Ready?

To better understand Canada's state of readiness, a set of necessary conditions was developed in consultation with regional and national experts. These conditions fell into three categories:

1) ***Infrastructure*** - Health and human resource infrastructure that includes communities of practice, resource planning and a digital infrastructure (informatics).

2) ***Operations*** - the ability to evaluate and , coordinate, and implement testing at a health system level including an entry point for innovation, an evaluative function, a model for coordinating service, and supports for care provider awareness and patient navigation.

3) ***Healthcare environment*** – the larger healthcare supports required including necessary approaches to financing, integrating innovation, education and training patients and providers, and regulating testing to ensure quality care.

Eleven essential conditions were then mapped to Canada's 4 largest provinces as well as Nova Scotia, the largest province in Canada's Atlantic region. Assessing the state of progress for each of these regions revealed varying states of readiness for genomic medicine.

Important gaps that will need to be addressed in Canada's future include:

- **Improving Informatics** – Is essential for test development, interpretation, and clinical decision support. Ensuring adequate integration of test results into electronic health records will also provide a key resource for real-world monitoring, disease management, quality assessment and assurance, and financing. Most provinces still lack sufficient data integration.
- **Evaluation/Health Technology Assessment (HTA)** – Fit for purpose HTA will be needed to identify high-value testing. Most provinces lack evaluative processes that adhere to HTA principles of timeliness, transparency, and engagement.
- **Navigational Tools** – Effective delivery of genetic testing requires navigation tools for patients and the public including referral guidelines, a test directory, eligibility criteria, tools/ education for ordering genetic testing, and a care clinic directory. Some of the provinces are working on these navigational resources.
- **Financing Approach** – Most provinces lack dedicated funding to facilitate rapid onboarding or a funding formula that supports test development and proficiency testing. The current reliance on the private sector to fund test development may be counterproductive as priorities are influenced by who is paying, rather than unmet need, equity, or efficiency.
- **Engagement** – High performing health systems require broad engagement of those impacted by testing. These include the patients, administrators, IT professionals, implementation and genome scientists, public and private sector innovators and others (scientists, legal and ethics experts, professional organizations, bioethicists, regulators).

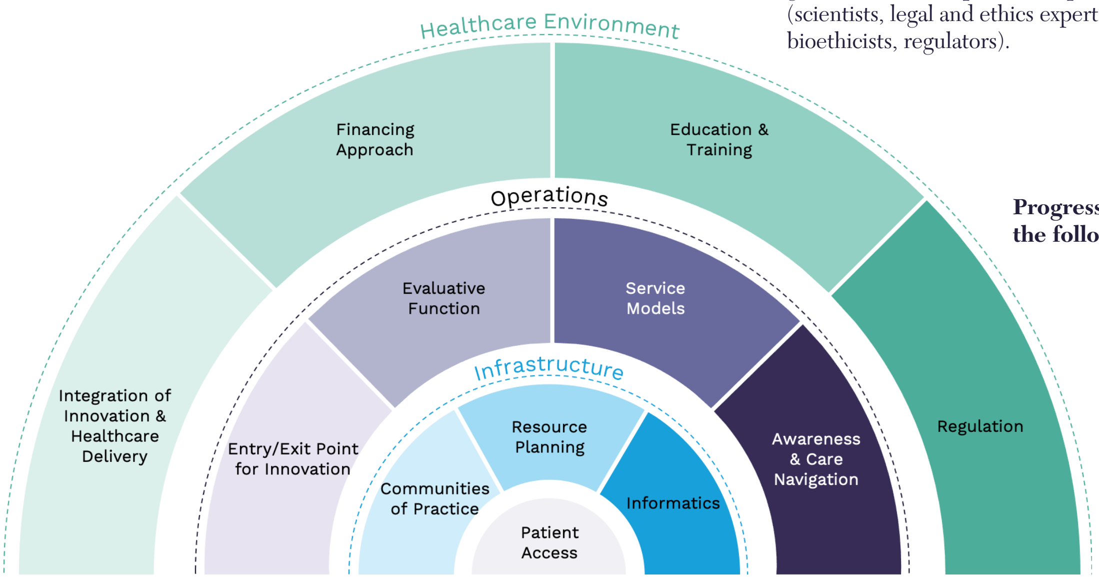

Progress by province is described in the following pages.

Where do the Provinces Stand?

Alberta and Quebec have created more of the necessary conditions for readiness, largely in part to the earlier establishment of single, laboratory service organizations and programs that provide the necessary infrastructure for coordination and planning as well as necessary operational conditions. In Nova Scotia, a higher level of coordination and planning is achieved due to lower levels of service demand and the ability of the government to work directly with the individual teaching hospitals who provide province-wide testing. However, many of the operational and evaluative processes are informal, and not public facing. The opposite is true in Ontario, which is challenged with much higher levels of demand for service, a complex web of formal evaluative processes, and until recently, a highly decentralized health system.

British Columbia

Strengths:

- Single service organization (Provincial Laboratory Medicine Services) that establishes a community of practice and supports resource planning.
- Single point of entry with explicit timelines for evaluation and coordination across service providers.
- Some integration of innovative testing.

Weaknesses:

- Lack of integration of laboratory information systems.
- Limited engagement and involvement of broader stakeholder community.
- Substantial opportunities to improve care navigation.

Priority Actions

- Create a cross-regional integrated laboratory information system and a plan for integration into electronic health records.
- Expand networks to include broader members of the innovation community,
- Improving the processes of navigation for care providers and patients and develop standards for education and training.

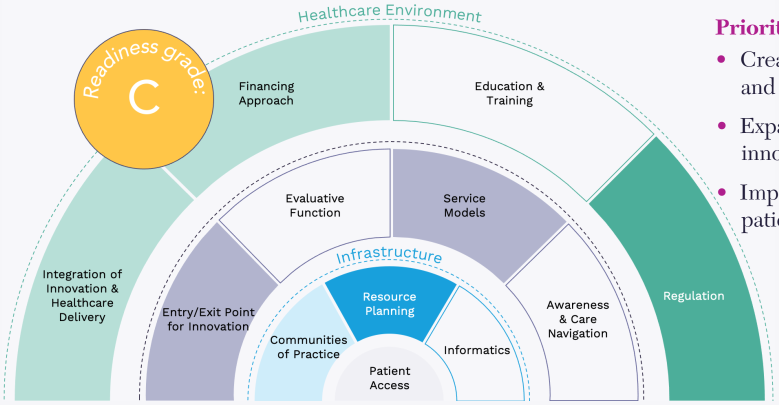

Its current state of readiness has earned British Columbia a grade of C

Alberta

Strengths:

- Single service organization (Alberta Precision Laboratories) that provides oversight and resource planning.
- Integration of laboratory information across province is established.
- Integration and exchange with innovators through dedicated translational research programs, open application processes and mainstream use of investigational testing.

Weaknesses:

- The test review process, timelines and criteria are not publicly available.
- There are still opportunities to improve care navigation and education standards for patients and providers.

Priority Actions

- Expand opportunities for engagement with broader members of the innovation community to improve healthcare planning and foster innovation.
- Improve the process of deliberation that surrounds the consideration and adoption of tests.
- Improving the financing approach to include funding for test development and to account for capital infrastructure, human resources, and other associated costs of testing.

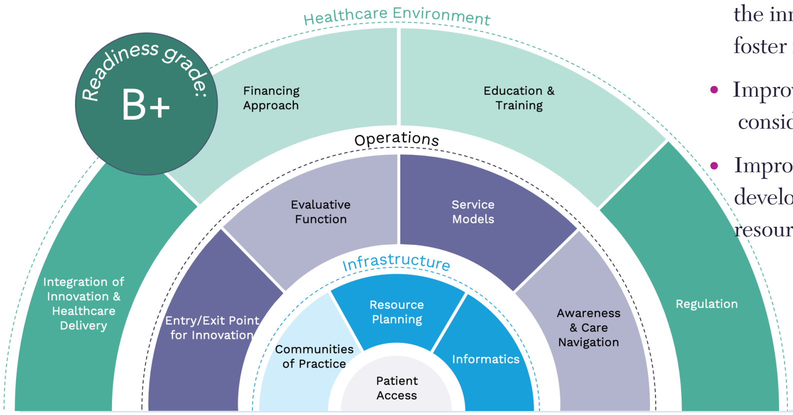

Its current state of readiness has earned Alberta a grade of B+

Needs Improvement
Partially Established
Established

Ontario

Strengths:

- Recently created single service organization (Provincial Genetics Program).
- Clear standards for accreditation and proficiency.

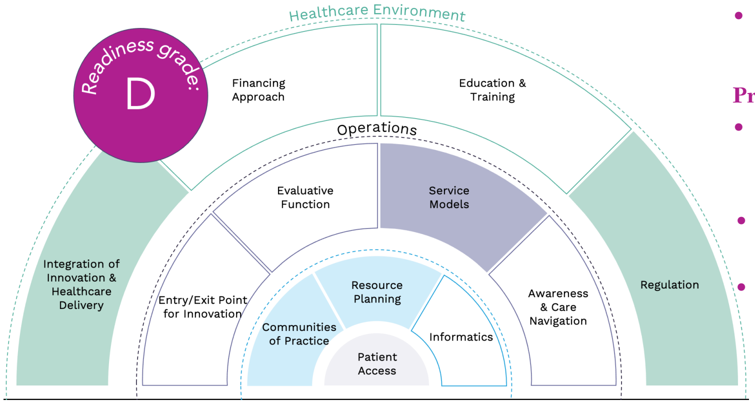

Weaknesses:

- Funding not timely or transparent; no funding for test development or human resources .
- No integration of laboratory information.
- Multiple evaluative frameworks.
- Limited engagement and involvement of broader stakeholder community.

Priority Actions

- Shift away from the Ministry acting as a decision-maker for the funding of individual tests, and toward a system of Ministry as a steward.
- Consolidate evaluation processes and adopt a single-entry approach, supported by horizon scanning.
- Create a laboratory information system integrated with clinical health records.

Its current state of readiness has earned Ontario a grade of D

Quebec

Strengths:

- Single service organization (Direction de la biovigilance et de la biologie médicale).
- Single point of entry and somewhat transparent evaluation.
- Nimble financing approach with funding available for test development.

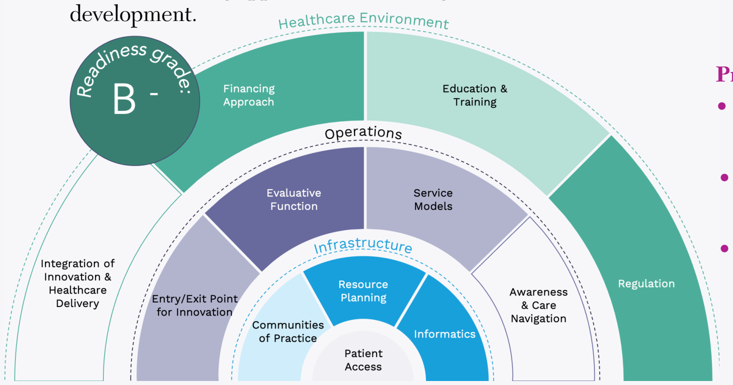

Weaknesses:

- Navigation and education for care providers and patients limited.
- Limited integration of innovation and healthcare delivery.
- Limited engagement and involvement of broader innovation community.

Priority Actions

- The DBBM should consider a separate advisory council for commercial innovators.
- Improve the processes of navigation for care providers and patients; develop standards for education and training.
- Further integrate innovative testing into the mainstream delivery of care, consistent with Quebec's goals within its Bureau of Innovation.

Its current state of readiness has earned Quebec a grade of B -

Nova Scotia

Strengths:

- Dedicated program (Pathology and Laboratory Medicine Program) that provides oversight and resource planning through key teaching hospitals.
- High level of service coordination.
- Integration of innovative testing.

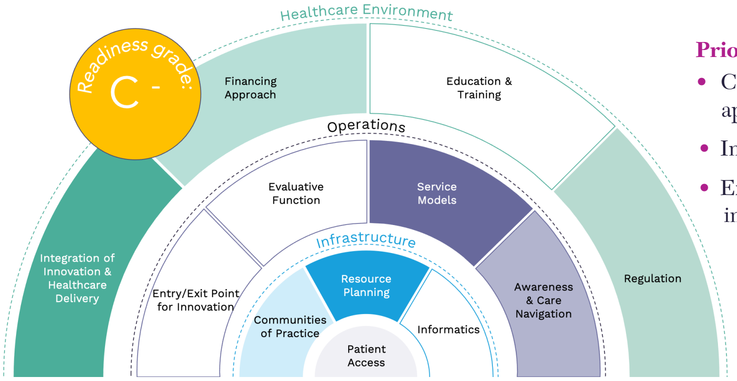

Weaknesses:

- No single entry point, explicit review process, timelines or criteria used to consider new tests.
- Lack of integration of laboratory information across centres.
- Limited engagement and involvement of broader stakeholder community.

Priority Actions

- Create a a transparent evaluation process and a single-entry approach, supported by horizon scanning.
- Integrate laboratory and clinical information systems.
- Expand engagement with broader members of the healthcare / innovation community, particularly commercial innovators.

Its current state of readiness has earned Nova Scotia a grade of C -

Needs Improvement
Partially Established
Established

More information about the State of Readiness Progress Report for Genomic Testing in Canada can be found here: TBD

Chapter 1. Background

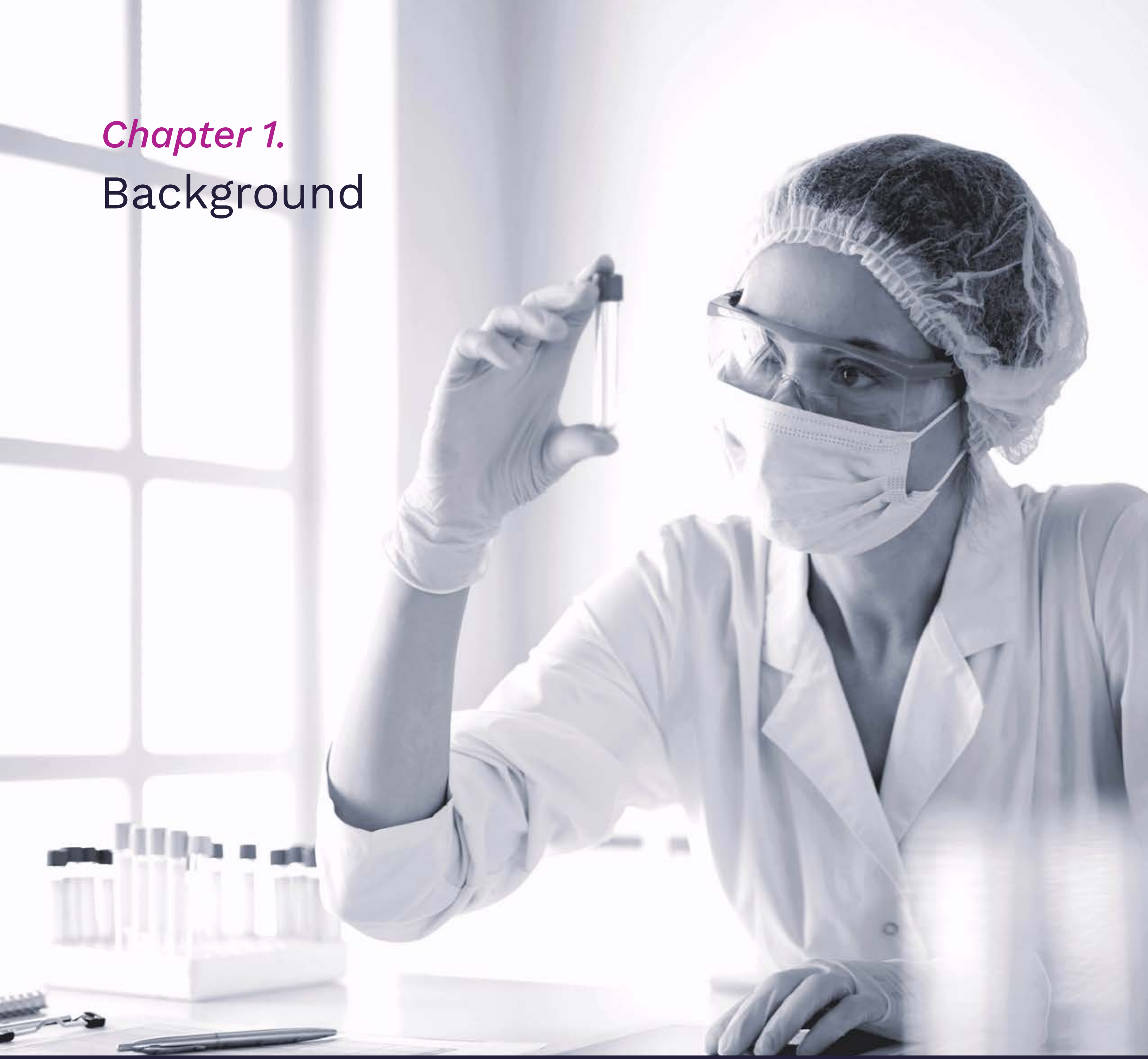

CHAPTER SUMMARY

- Genomic medicine plays an important role in scientific discovery as well as diagnosing disease, predicting and monitoring response to therapy, and understanding disease prognosis.
- In the immediate future, genome-based testing will play a heightened role in prenatal and neonatal care, oncology, rare diseases and diseases amenable to cell and gene therapy.
- Fully integrating testing into healthcare service delivery has the potential to reduce overall health system costs, while reducing harm or improving health benefits for patients.

Why Genome-Based Testing?

Effective patient care requires information about the nature of underlying disease (etiology/pathophysiology), what condition a patient has (diagnosis), what outcomes can be expected (prognosis), and how likely a patient may respond to any given inter-vention (prediction/monitoring). [1] Increasingly, laboratory-based biomarkers that measure the expression, function and regulation of genes directly (through cytogenetics or various genomic tools), or their protein products (for example by immunohistochemistry) are being used for this purpose. These genome-based tests have already become a hallmark of decision-making in oncology, given cancer is a genetic disease and may be amenable to targeted therapy. They are also increasingly used to detect hereditary conditions or to establish risk of future disease. In Canada, in addition to its use in oncology, genetic testing is used extensively in newborn screening and within hereditary disease programs.

DEFINING TERMS:

Personalized/precision medicine – an approach to tailoring disease prevention and treatment that takes into account differences in people's genes, environments, and lifestyles"

Genomic medicine –the use of laboratory-based biomarkers that measure the expression, function and regulation of genes and gene products to aid healthcare decision making and scientific discovery.

Genomic medicine, advanced testing, genetic testing, and genome-based testing may be used interchangeably throughout this report.

The rapidly developing field of genomic medicine and genome-based testing has led to an increasing number of administrative decisions regarding the implementation of new testing. These include decisions about different technical platforms (e.g., single-gene, multi- gene, whole-exome, and whole-genome sequencing and expression analysis); modalities (tissue, saliva, blood, or urine-based sampling); location (laboratory-based or delivered at point-of-care); provenance (commercially available in vitro diagnostic tests and services versus in-house/ laboratory developed tests); and timing and sequencing of tests. All of these factors affect how patients and health systems may benefit ('clinical utility'), including cost and patient outcomes, and broader health system goals such as caregiver and patient experiences. They will also influence how care is delivered.

Unlike traditional healthcare technologies, genetic and genomic ('genome-based') biomarker testing also provides research opportunities beyond healthcare decision making, and can lead to discoveries about the nature of disease or effectiveness of current and future therapies. These opportunities are being developed in 'real time', side by side with established clinical utility— blurring the boundaries between research and standard practice. For example, tests may qualify patients for clinical trials, which has important clinical, scientific and economic benefits.[2] The value of integrating research in clinical practice was forcibly clear during the COVID-19 pandemic, where rapid research-led development and implementation of testing capacity was essential for both monitoring the pandemic, and the development of preventive and therapeutic strategies. Yet the value of research and discovery when adopting new healthcare technology is typically not considered by health technology assessment processes in most single payer systems.[3]

Given the potential for exponential growth of new tests and test approaches, and the complexity of introducing them, health system planners preparing for a future of genomic medicine need to grapple with how these services can be delivered effectively and efficiently . As the structure, remit and organization of healthcare systems (and the laboratory functions within them) vary, there are likely to be no one-size-fits-all solution; however , some necessary conditions will be required to manage these technologies in a way that benefits patient and is sustainable.

Use of test	Example in cancer	Why test?	Test performed	Outcome
**Diagnosis**	33-year-old female presented with 2-month history of kidney stones and abdominal pain at night. No fever, but blood in urine.	Genetic mutation leads to protein expression that indicates disease.	Immunohistochemistry test revealed positivity for CD20 and Ki67.	Confirmed diagnosis of diffuse large B-cell lymphoma (DLBCL).
**Prognosis**	55-year-old male with diagnosis of chronic lymphocytic leukemia (CLL) and TP53.	Lack of gene leads to more unstable blood cell development and disease.	Molecular genetic test for TP53 mutation.	Confirmed mutation predicts poor disease prognosis and response to conventional therapy. Patient will receive specialty drug, ibrutinib.
**Prediction and monitoring**	A 67-year-old female previously diagnosed with (stage IV) metastatic colorectal cancer (mCRC).	Normal (wild-type) gene ensures drug binding to EGFR protein will reduce cell proliferation.	Molecular testing for (wild type) K-ras gene.	Confirmed lack of mutation predicts improved response and lower costs from use of specialty anti-EGFR drugs (cetuximab or panitumumab).
**Research**	A 41-year-old male abdominal pain consistent with gallstones has scan revealing cholangiocarcinoma, which spread to liver. Received two courses of therapy with no response.	There may be genes expressed that will qualify patient for investigational drug trials or research.	Molecular diagnostic testing was performed, which revealed positivity for FGFR2-BICC1 fusion, MYC amplification, and NF2 inactivation.	Alterations that activate FGFR2 may confer sensitivity to FGFR inhibitors, such as investigational drugs pazopanib and ponatinib. Patient was enrolled in ongoing trial.

Anticipating Future Needs

Since the first human genome was sequenced, there has been a lot of enthusiasm about the potential for genome-based medicine. This has led to a rate of technological innovation that has outpaced the available evidence required to estimate its future impact.

A 2020 survey of 84 Canadian genetic professionals with leadership roles in all publicly funded genetic clinics in Canada, a majority of respondents felt that the use of genome-based testing would be expanded to 2030 across 4 key areas.[4] Some of the relevant findings of the survey:

Prenatal and newborn screening

- 95% of survey respondents felt there would be a move toward cerebral fluid DNA non-invasive prenatal screening as a first-line test for determining whether there are missing or extra chromosomes.
- 66% believed cerebral fluid DNA non-invasive prenatal screening would be routinely offered in preconception settings to all couples planning pregnancies.

Oncology

- 72% believed guidelines for considering who is eligible for germline genetic testing based on family history will be expanded to include all types of cancer.
- 61% believed tumor testing will be performed using genome-wide screening instead of single- or multi-gene panel testing.

Neonatal care

- 100% of respondents believe genome-wide screening will be routinely offered for patients with suspected genetic disease in the neonatal and pediatric intensive care setting.
- 94% believe individuals who currently undergo chromosomal microarray analysis for a neurodevelopmental disorder will receive whole-genome sequencing instead.
- 88% believe individuals who currently receive a gene panel for a neurodevelopmental disorder will receive genome-wide sequencing instead.

Service delivery

- 53% believed post-test genetic counselling will be fully automated in most cases when the genetic test results are negative or uninformative.
- 53% believed regulatory changes will lead to genetic counseling being offered independently in Canadian provinces for patients who do not require physician-protected tasks.

The survey results highlight the potential impact on models of service delivery as well as potential impacts on wait times, costs, and length and quality of life. Other areas of medicine likely to drive the need for genome-based testing include:

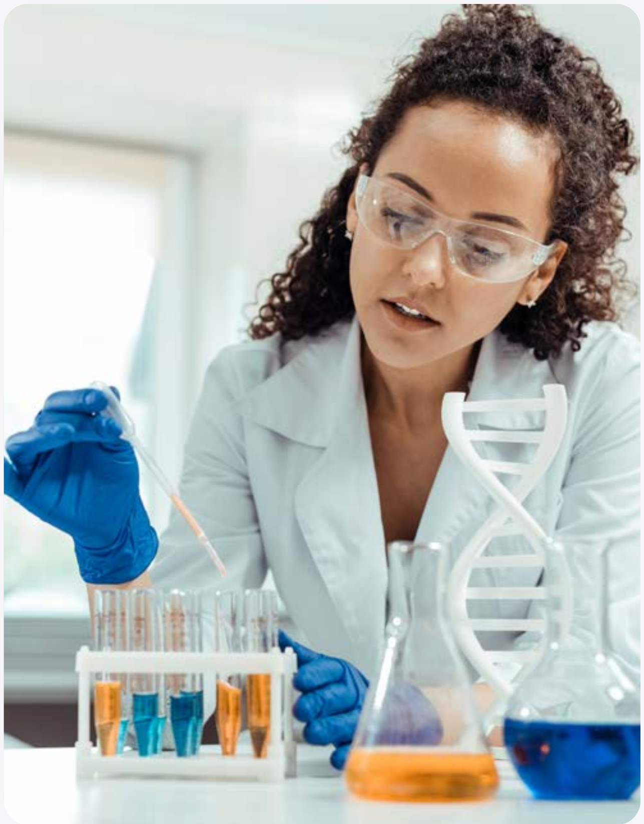

Rare Diseases

Many rare diseases are characterized by inborn errors of metabolism which leads to defects of single genes, which code for enzymes that aid normal metabolism. Symptoms of these rare diseases may appear when a child has an accumulation of toxins or deficiency of essential metabolites from the defect. While biochemical, functional and other testing can be used to aid diagnosis of these rare conditions, genetic testing is recognized as a means of obtaining an accurate diagnosis in a timelier manner.[5]

Many rare diseases of this type have traditionally used lifestyle (diet and supportive therapy), as an intervention (in many cases, not successfully). There are, however, a growing number of treatment options, including enzyme replacement therapy or other interventions that aid metabolism and removal of toxins; advances in cell and organ transplantation, and upcoming gene therapies to address these recognized defects.

- There are estimates of between 5000-8000 rare diseases with 95% of these currently without regulatory approved treatments.
- Access to testing for rare disease widely varies by province and is heavily reliant on out-of-province providers.
- In the US in 2019, there were 838 orphan indications granted to 564 drugs since the US Orphan Drug Act ; the number of drugs having more than one indication is increasing. (Figure 2)

Figure 2: *Number of drugs and indications approved with an orphan drug designation in the US up to 2019*

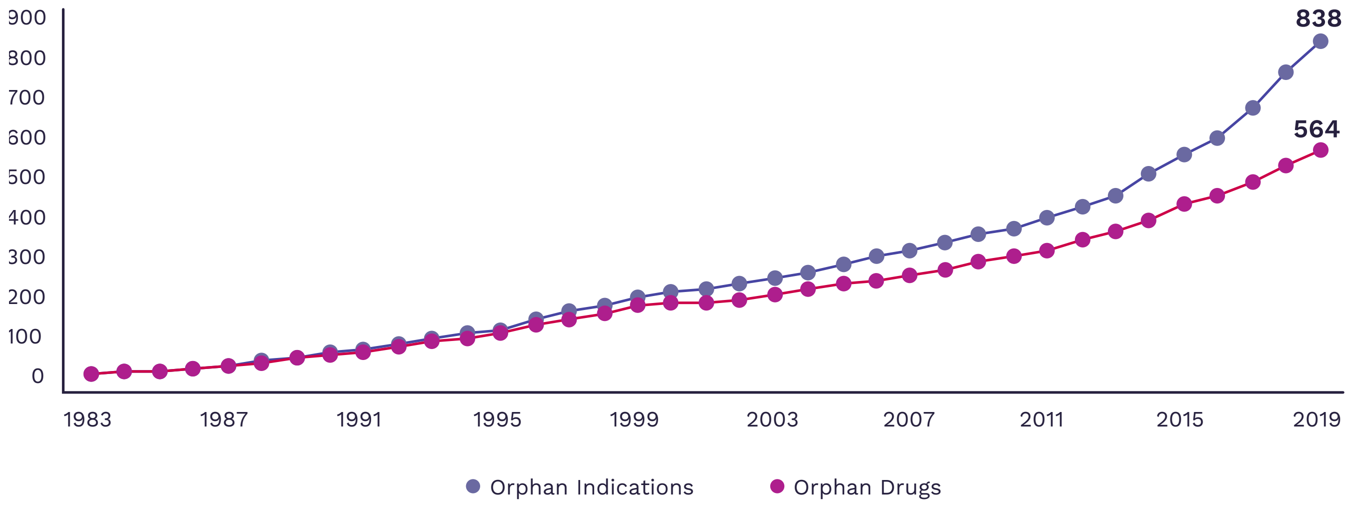

Source: IQVIA Institute, Aug 2020; FDA Orphan Drug Designations and Approvals[8]

Oncology

As a genetic disease where prevalence will increase with an aging population, oncology will continue to drive the need for genome-based testing. Some examples:

- Single biomarkers are now relevant across multiple tumour types, and within single tumour types, genomic biomarkers are increasingly required to qualify patients for therapies.
- While the number of new drugs requiring testing is increasing (Figure 3), the number of new genes that will be required for testing outside of companion diagnostics could outpace the introduction of drugs.
- Recent worldwide efforts across thousands of scientists to understand the role of genetics such as the Pan-Cancer Analysis of Whole Genomes Consortium[6] have given unprecedented view of the genetic changes that can contribute to cancer:
  - It is now known that almost all (95%) tumours have identifiable mutations that are responsible for (i.e., driver mutations) the growth of cancerous tissue.
  - Driver mutations can occur years before cancer is diagnosed, which has implications for early detection and biomarker development.

Figure 3: *U.S. oncology medicines that recommend or require pharmacogenomic testing on their prescribing labels prior to use*

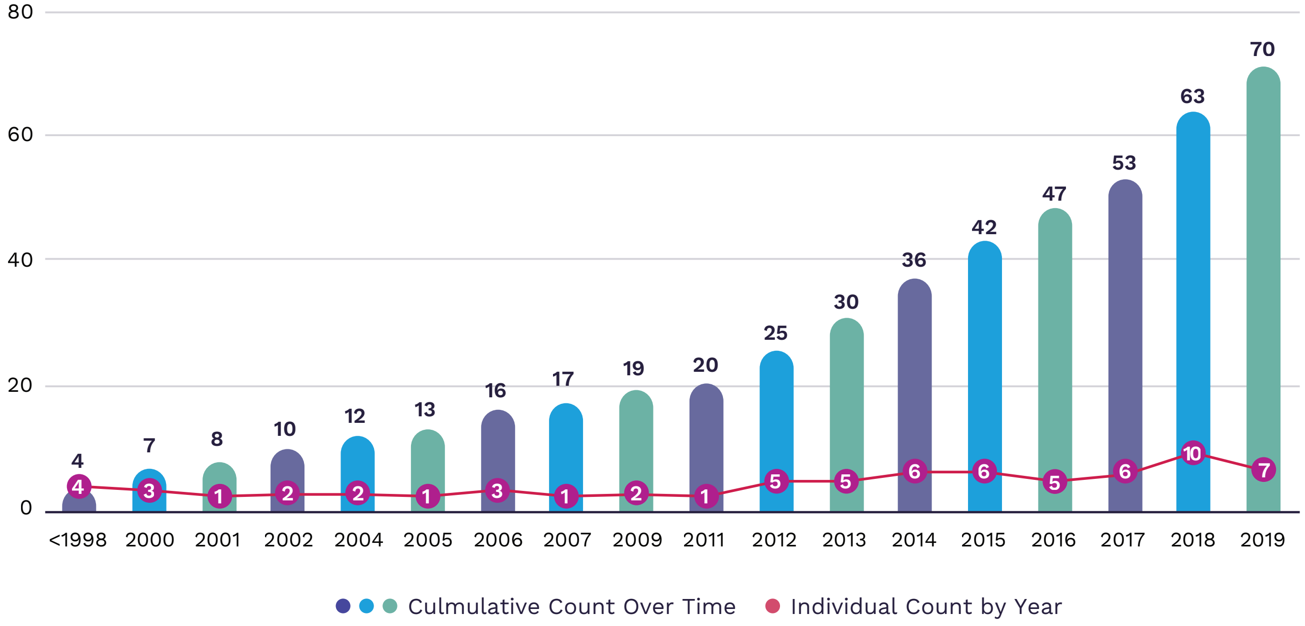

Source: IQVIA Institute, Supporting Precision Oncology: Targeted Therapies, Immuno-oncology, And Predictive Biomarker-based Medicines, Aug 2020

Notes: U.S. oncology medicines that recommend or require pharmacogenomic testing on their prescribing labels prior to use. The list includes oncology therapies that gained approval for indications outside of the initial regulatory approval that subsequently recommended or required a biomarker test prior to use.

Cell and Gene Therapies

There were 804 cell and gene therapies registered in Phase 1 studies in 2021, with more than 40% of these being investigated in cancer. Almost 100 of these therapies were chimeric antigen receptor T (CAR-T) and natural killer (NK) cell therapies. Genome-based testing, including more high-throughput comprehensive genomic profiling, will play an essential role in the appropriate use and implementation of these new therapies, many of which promise to cure disease.

Figure 4: *Cell and gene therapies registered in regulatory filings, 2011 to 2021[7]*

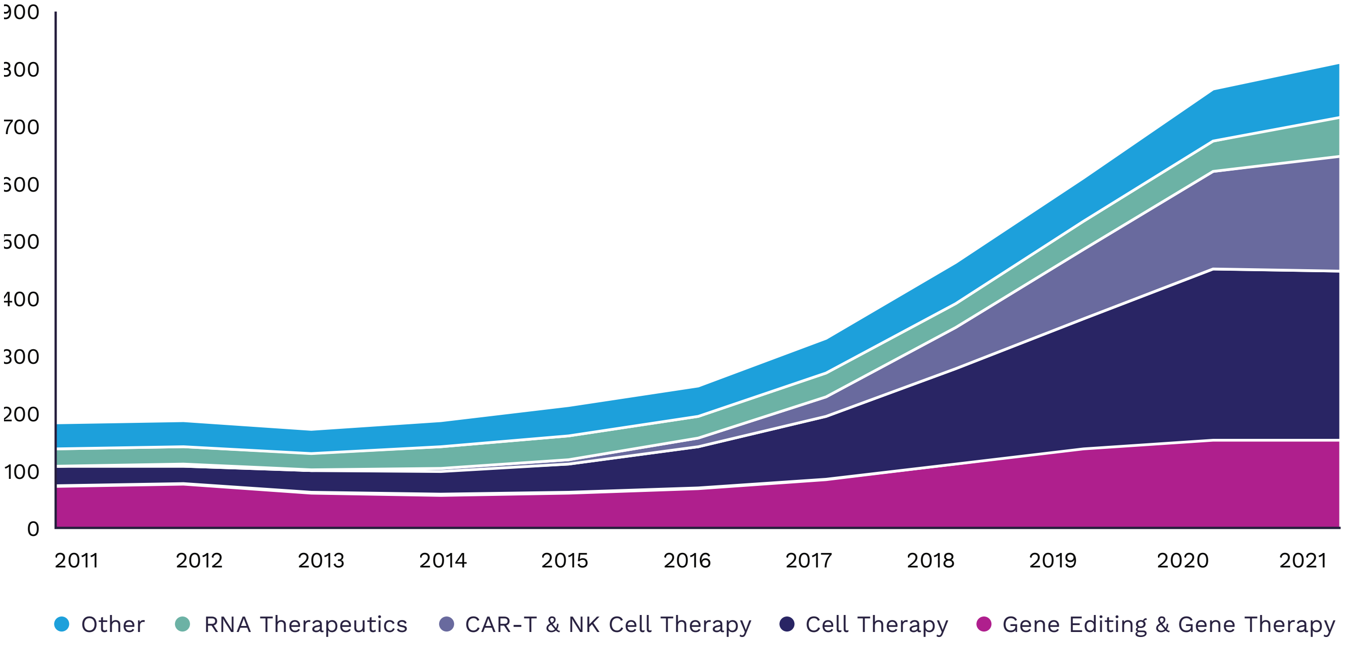

Source: IQvia Pipeline Intelligence, Dec 2021; IQVIA Institute, Jan 2022.

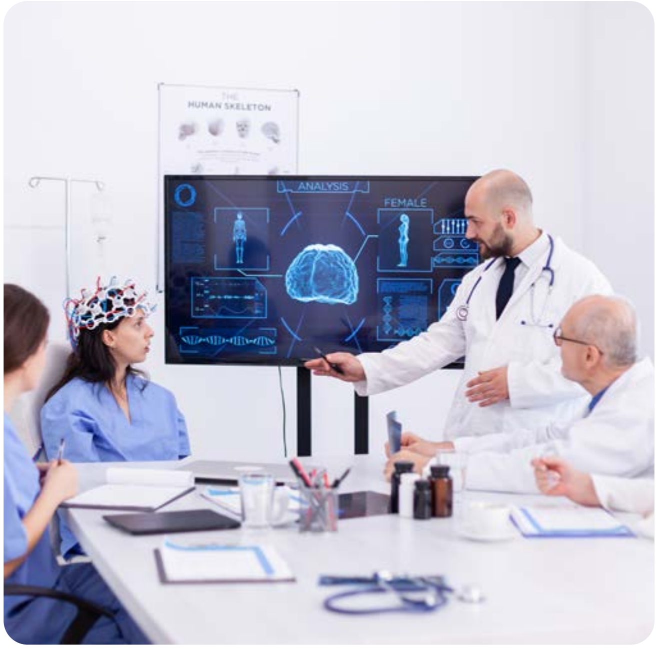

Other Therapeutic Areas

Genome-based testing is expected to anticipate other therapeutic areas that significantly affect patients and patient care. This includes autoimmune diseases (such as rheumatoid arthritis[8], multiple sclerosis[9], systemic lupus erythematous[10], and Crohn's disease[11]), ophthalmologic conditions[12], lung diseases (asthma[13], chronic obstructive lung diseases[14], interstitial lung diseases[15]), infectious disease[16], and neurological conditions[17]. Many of these represent considerable healthcare expenditure today.

Improving Health and Care Experiences for Patients and Care Providers

The most compelling need to act sooner rather than later is to improve patient experiences, reduce patient harm, increase patient benefit, improve equity of access and reduce delays in uptake of valuable tests:

Creating Opportunities for Improving Health and Experiences for Patients and Providers

Lung cancer is the leading cause of cancer-related death in Canada leading to 1 in 4 of all cancer-related deaths. Patients with non-small cell lung cancer (85% of all lung cancer) have a poor prognosis for survival, with 1 in 4 patients surviving past 5 years. Newly diagnosed patients may undergo a tumour biopsy so that tumour tissue can be tested for genetic mutation. The biopsy procedure can result in excessive waiting for patients (for procedure related resources such as recovery beds), or direct harm to patients[18] or may be more difficult to perform depending on the location of the tumour.

In addition, up to 10% of patients geting biopsy procedures have insufficient tissue for traditional approaches to testing (e.g. single-gene or multigene testing). More than 25% of those with adequate tissue may still lack sufficient detectable DNA in their samples or see their tissue samples exhausted after one or two tests. While this means the patient will require an additional biopsy, it may not be possible to then take additional samples because of time or resource constraints.

Newer testing modalities have now been developed that can spare lung tissue.[19] There are also emerging tissue- and blood-based point-of-care tests for single genes, that can be administered by an oncologist and yield within 3 hours.[20] The availability of these new tests means the ability to identify many more patients (and more quickly) who would benefit from less-toxic, targeted therapies. A Canada-specific analysis projected an additional 346 patients with lung cancer in Canada could benefit from this approach in the next 3 years, resulting in a additional 1.6 years of life (on average) for each patient.

Reducing Healthcare Costs

Unlike traditional testing, genome-based testing requires specialized personnel dedicated to bioinformatics, enhanced laboratory procedures, and genetic counselling. However, it can greatly reduce the need for other specialty personnel, including genetic subspecialists, radiologists, pathologists, and others. In some cases, this may increase diagnostic yield, reduce time and patient appointments.

The impact of providing whole-exome sequencing (WES) as an early, routine clinical test for infants with suspected monogenic disorders, based on real-world experience in Australia, provides a telling example. While testing and the need for additional health human resources (genetic counsellors) introduces additional costs, there are also a number of services not required. These changes not only result in more effective disease diagnoses (25 versus 7 diagnoses) and a patient journey that involves fewer appointments and referrals, they also reduce average per-patient costs.

Costs for 40 patients, $AUD

Assessments and tests	Standard diagnostic care	Whole-exome sequencing first line
Genetics appointments	$ 22,239.24	
Subspecialist appointments	$ 9,187.83	$0.00
Pathology		
▪ Anatomic pathology	$ 14,409.32	
▪ Basic biochemistry	$ 4,289.12	
▪ Complex biochemistry	$ 9,437.04	
▪ Serology/immunology	$ 1,520.72	
▪ Other		
Diagnostic imaging	$ 50,165.45	
Electrophysiology	$ 22,027.97	
Genetic testing		
▪ SNP microarray	$ 23,880.00	$ 23,880.00
▪ Other genetic tests	$ 25,151.79	
Other		
▪ Medical photography	$ 809.62	
▪ DNA extraction and shipping	$ 2,541.00	
OT/anaesthesia costs	$ 3,693.53	
Geneticist appointment (new or review)	-	$ 18,303.60
Geneticist appointment review	-	$ 14,642.80
Genetic counselor appointment	-	$ 7,358.40
Genetic counselor appointment review	-	$ 5,886.80
WES (sequencing, analysis, reporting)	-	$ 80,000.00
TOTAL costs	$ 189,352.53	$ 150,071.60
TOTAL number of diagnoses per 40 patients	7	25
Incremental cost per diagnosis (95% CI)		-2,182.27 (-5,855.02,129.92)

Table 1 *Cost savings (avoidance) associated with whole-exome sequencing as an early routine test for infants with suspected genetic disease, adapted from [21]*

Cost Savings from Test Procurement or Provision

Testing within Canada can also significantly lower costs per test, and represent significant cost savings to the healthcare system. An analysis of whole exome sequencing used as a second-tier test (after chromosomal testing fails to produce a diagnosis) in Ontario was projected to have an annual budget impact (savings) of $CAD -3.45 million, in part due to the avoidance of other testing, but largely due to a 23% price reduction in the cost of providing the test ($3,444 vs. $4,589) through a centralized provider within Ontario.[22] Similar analyses of cost savings from test "repatriation" have been conducted in Quebec. For example, the estimated 3-year cost savings from conducting all tests for hereditary cardiovascular disease using massive parallel (i.e., 'next-generation') sequencing approaches in Quebec is projected to be $1.2 million, with a plausible range between $750,000 and $1.8 million.[23]

Creating Opportunities for Innovation

Many provinces continue with a legacy scheme of not funding tests if they are not clinically actionable, i.e., there are no currently funded targeted treatments that strictly rely on the results of the test. However, patients who have exhausted all of their options are regularly enrolled into research programs including clinical trials. In some cases, they require testing information to qualify them for their enrollment.

While patients may pay out-of-pocket for unfunded tests, this will not be an option for many. Patients who are able to pay for testing may receive the benefits of future innovation through clinical trials or compassionate access programs. However, the healthcare system may not be able to further learn from their testing information if it is sold privately to patients. Despite this disconnect, increasing enrollment in clinical trials and encouraging innovation through genetic testing are active policy priorities in Canada.

Equity of Access to Testing

While the introduction of tests is regulated by Health Canada, not all provinces and territories have the same capacity for delivering genetic testing or reimburse the same types of tests:

- There is no service coordination across provinces, and each province has different test availability. Each province has different processes for considering what types of tests to fund and different performance and quality standards.
    - Larger provinces (ON, QC, AB, BC) often have more than one facility that can deliver testing. The level of coordination and standardization varies by province. This has created a need to establish dedicated programs to coordinate the implementation of testing. Some larger provinces lack regional analytic standards, so test performance (and its ability to benefit patients) varies across institutions within the province.
    - Smaller provinces typically rely on an individual institution to make decisions in consultation with government. This reduces variation in test performance within the province and, in some cases, makes providing access more straightforward.
    - However, unlike programs in larger provinces, processes governing testing are less transparent to the public.
- All provinces additionally pay for out-of-province testing, either due to capacity constraints or when the quality of testing is dependent on an adequate number of referrals. They may use different out-of-province providers, and in some cases refer to other provinces.
- There are no test directories, or standards for coding and tracking the use of tests country-wide. Most provinces use different approaches to tracking for internal use. The lack of publicly accessible test lists and varying levels of capacity and ability to track what tests are being performed , creates challenges for patient and provider care navigation as well as innovators of new tests.

These factors together have resulted in testing availability according to postal code, and with a limited understanding of their use, costs or real-world performance.

New Policy Approaches and Models of Service Delivery are Emerging

A significant challenge with integrating genome-based testing into the healthcare environment is the need for revisiting the role of traditional laboratory services and care pathways. Delivering effective and efficient testing also requires system-wide coordination that considers the potential value of new tests, coupled with appropriate coordination of care, quality assurance standards, and a system that recognizes clinical and translational research opportunities outside of regular care. All of these challenges combined speak to a need to revisit historical models of technology management, including the governance, administration, and financing of laboratory services.

Some countries, such as Australia and the United Kingdom, are moving toward more comprehensive approaches for biomarker testing including whole-genome sequencing for every cancer patient's tumor to guide treatment. These are being supported by large programs, such as the NHS England Genomic Medicine Service, which have been built on a commitment of consistent and equitable care, common national standards, a single national test directory, giving patients opportunities to participate in research, and building a genomic knowledge base to inform innovation.[24]

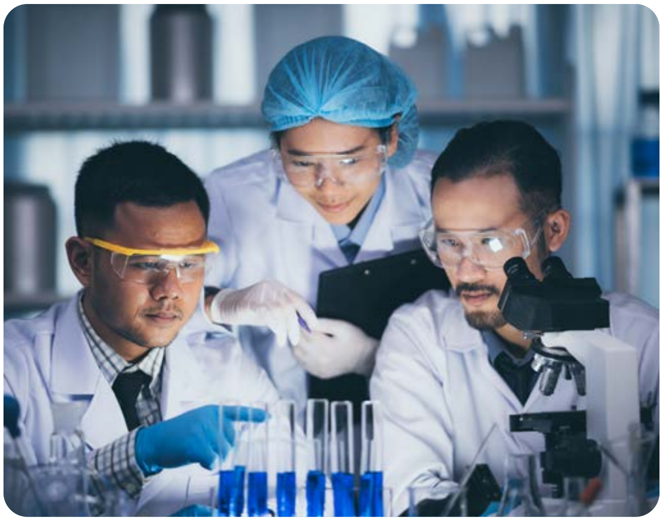

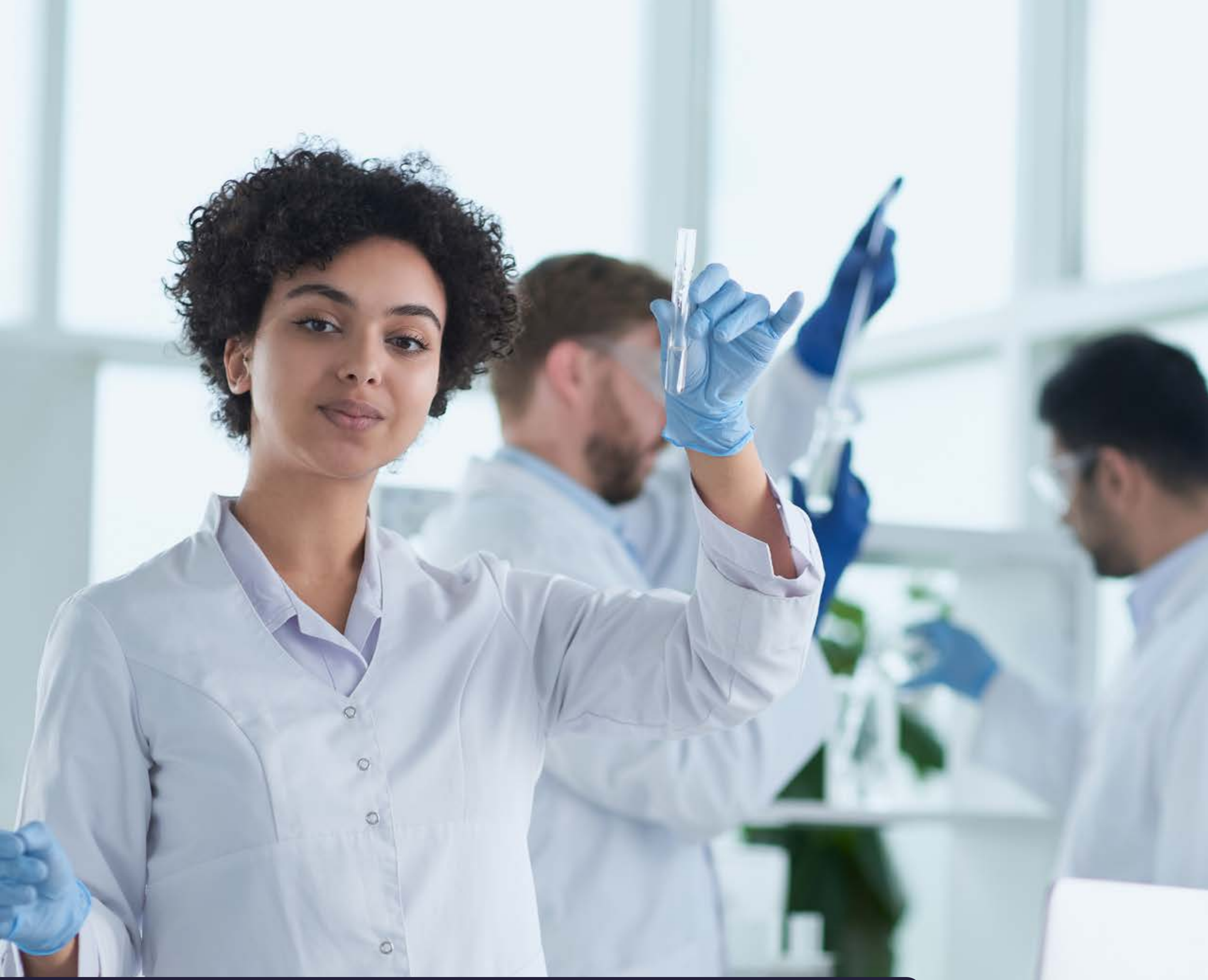

Purpose of this Report

Patients, policymakers and care providers that benefit from genomic medicine, are interested in developing a comprehensive understanding of barriers to its broader dissemination in Canada and potential solutions to remedy these.

As a starting point, and recognizing that readiness does not happen overnight and requires concerted strategic efforts over time by healthcare systems, a "State of Readiness" Progress Report has been developed for advanced diagnostic testing as a way of objectively communicating the state of readiness of Canadian jurisdictions to appropriately deliver advanced testing services.

References

1. Califf RM. Biomarker definitions and their applications. *Exp Biol Med (Maywood)* 2018;**243**:213–21 doi:10.1177/1535370217750088
2. Tran DT, Akpinar I, Jacobs P. The Costs of Industry-Sponsored Drug Trials in Canada. *PharmacoEconomics Open* 2020;**4**:353–9. doi:10.1007/s41669-019-0161-0
3. Polisena J, Castaldo R, Ciani O, *et al.* HEALTH TECHNOLOGY ASSESSMENT METHODS GUIDELINES FOR MEDICAL DEVICES: HOW CAN WE ADDRESS THE GAPS? THE INTERNATIONAL FEDERATION OF MEDICAL AND BIOLOGICAL ENGINEERING PERSPECTIVE. *International Journal of Technology Assessment in Health Care* 2018;**34**:276–89. doi:10.1017/S0266462318000314
4. Borle K, Kopac N, Dragojlovic N, *et al.* Where is genetic medicine headed? Exploring the perspectives of Canadian genetic professionals on future trends using the Delphi method. *Eur J Hum Gene*t 2022;**30**:496–504. doi:10.1038/s41431-021-01017-2
5. Johnson K, Saylor KW, Guynn I, *et al.* A systematic review of the methodological quality of economic evaluations in genetic screening and testing for monogenic disorders. *Genet Med* 2022;**24**:262–88. doi:10.1016/j.gim.2021.10.008
6. Campbell PJ, Getz G, Korbel JO, *et al.* Pan-cancer analysis of whole genomes. *Nature* 2020;**578**:82–93. doi:10.1038/s41586-020-1969-6
7. iqvia-institute-global-trends-in-randd-to-2021.pdf. https://www.iqvia.com/-/media/iqvia/pdfs/institute-reports/global-trends-in-r-and-d-2022/iqvia-institute-global-trends-in-randd-to-2021.pdf (accessed 31 Aug 2022).
8. Gavan S, Harrison M, Iglesias C, *et al.* Economics of stratified medicine in rheumatoid arthritis. *Curr Rheumatol Rep* 2014;**16**:468. doi:10.1007/s11926-014-0468-x
9. Caprariello AV, Adams DJ. The landscape of targets and lead molecules for remyelination. *Nat Chem Biol* 2022;**18**:925–33. doi:10.1038/s41589-022-01115-2
10. Bossini-Castillo L, López-Isac E, Martín J. Immunogenetics of systemic sclerosis: Defining heritability, functional variants and shared-autoimmunity pathways. *J Autoimmun* 2015;**64**:53–65. doi:10.1016/j.jaut.2015.07.005
11. Liu Z, Shen B. Overcoming difficulty in diagnosis and differential diagnosis of Crohn's disease: the potential role of serological and genetic tests. *Expert Rev Mol Diagn* 2015;**15**:1133–41. doi:10.1586/14737159.2015.1068121
12. Burdon KP. The utility of genomic testing in the ophthalmology clinic: A review. *Clin Exp Ophthalmol* 2021;**49**:615–25. doi:10.1111/ceo.13970
13. Bansal M, Garg M, Agrawal A. Advances in asthma genetics. *Adv Genet* 2021;**107**:1–32. doi:10.1016/bs.adgen.2020.11.001
14. Melro H, Gomes J, Moura G, *et al.* Genetic profile and patient-reported outcomes in chronic obstructive pulmonary disease: A systematic review. *PLoS One* 2018;**13**:e0198920. doi:10.1371/journal.pone.0198920
15. Kheir F, Uribe Becerra JP, Bissell B, *et al.* Use of a Genomic Classifier in Patients with Interstitial Lung Disease: A Systematic Review and Meta-Analysis. *Ann Am Thorac* Soc 2022;**19**:827–32. doi:10.1513/AnnalsATS.202102-197OC
16. Zella D, Giovanetti M, Cella E, *et al.* The importance of genomic analysis in cracking the coronavirus pandemic. *Expert Rev Mol Diagn* 2021;**21**:547–62. doi:10.1080/14737159.2021.1917998
17. Kadlubowska MK, Schrauwen I. Methods to Improve Molecular Diagnosis in Genomic Cold Cases in Pediatric Neurology. *Genes (Basel)* 2022;**13**:333. doi:10.3390/genes13020333
18. Lang D, Reinelt V, Horner A, *et al.* Complications of CT-guided transthoracic lung biopsy. *Wien Klin Wochenschr* 2018;**130**:288–92. doi:10.1007/s00508-018-1317-0
19. Cell-Free Circulating Tumour DNA Blood Testing to Detect EGFR T790M Mutation in People With Advanced Non–Small Cell Lung Cancer - Health Quality Ontario (HQO). https://www.hqontario.ca/evidence-to-improve-care/health-technology-assessment/reviews-and-recommendations/cell-free-circulating-tumour-dna-blood-testing-to-detect-egfr-t790m-mutation-in-people-with-advanced-nonsmall-cell-lung-cancer (accessed 3 Oct 2020).
20. Idylla Oncology Assays | Biocartis. https://www.biocartis.com/en-US/meet-idylla/idylla-oncology-assays (accessed 3 Oct 2020).
21. Stark Z, Schofield D, Alam K, *et al.* Prospective comparison of the cost-effectiveness of clinical whole-exome sequencing with that of usual care overwhelmingly supports early use and reimbursement. *Genetics in Medicine* 2017;**19**:867–74. doi:10.1038/gim.2016.221
22. Ontario Health (Quality). Genome-Wide Sequencing for Unexplained Developmental Disabilities or Multiple Congenital Anomalies: A Health Technology Assessment. *Ont Health Technol Assess Ser* 2020;**20**:1–178.
23. Summary - Hereditary Cardiovascular diseases NGS Panel. ;:6.
24. NHS England » NHS Genomic Medicine Service Alliances to help embed genomics into patient care pathways. https://www.england.nhs.uk/blog/nhs-genomic-medicine-service-alliances-to-help-embed-genomics-into-patient-care-pathways/ (accessed 23 Jun 2022).

Chapter 2.
Overview of the Progress Report

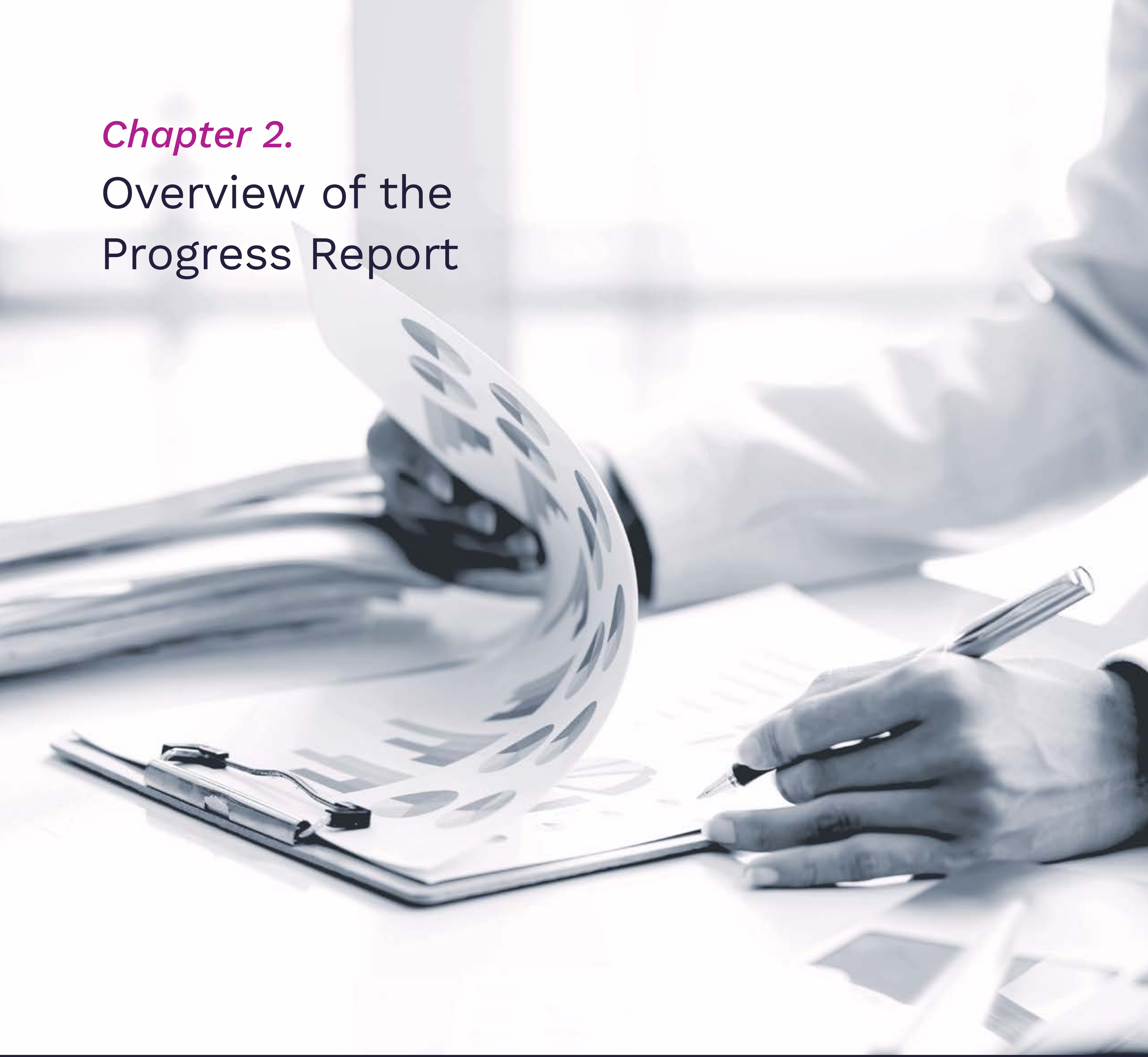

CHAPTER SUMMARY

- The National State of Readiness Progress Report was developed by first creating a list of conditions required for delivering state-of-the-art genomic medicine in any jurisdiction through literature review and consultation and peer-review by national and International subject experts. (Section 3)
- The Progress Report (Section 4) has been validated with regional experts; is intended to provide a snapshot of the current state of readiness, and to inform further discussion on opportunities to provide equitable access to genomic medicine across Canada.
- The potential humanistic, economic and broader societal impact of improving Canada’s state of readiness for genomic medicine is described in Section 5.

A schematic depicting the approach to this research is seen in Figure 6

Information Gathering

The key steps to developing the National State of Readiness Progress Report follow a mixed methods approach. First, a steering committee consisting of sponsor representatives was created to inform the scope and validate findings from the report. All sponsors contributed equally to the project.

Second, a narrative literature review was conducted based on a purposive sample of commercially published and grey literature. Replicable searches (Appendix A) were performed by a medical librarian specialist and relevant information was identified by a single reviewer (DH), and a background document describing conditions necessary for testing and good practices was prepared.

In parallel, three international experts were identified as well as two to four key informants (from each of Canada's five regions (Ontario, Quebec, British Columbia, Alberta, and Nova Scotia). The five regions chosen in this report were informed by population, geographic size and regional representativeness.

A semi-structured interview guide was developed (Appendix B). The stated purpose of the interview was to: 1) identify current challenges with the uptake and routine delivery of advanced diagnostic testing; and 2) explore what conditions are necessary and desirable for creating robust systems of advanced diagnostic testing (region-specific for Canadian informants, or generally, for international experts). Key informants, sponsors and sponsor delegates consulted are listed in the Acknowledgements section of the report.

Create and Validate List of Conditions and Good Practices

A preliminary list describing key enabling conditions and good practices was then developed and pared down through subsequent interview and consultation with expert informants. The list and good practices were then subject to peer-review (in an academic journal). These conditions appear in Section 3.

Develop Progress Report

Good practice items were then compared to current practice in Canadian jurisdictions and validated by original and additional key informants to create the Progress Report (Section 4). The draft report combined Sections 3 and 4 with additional sections on the unmet need (Section 1) and impact (Section 5) of genome-based testing within the Canadian healthcare and innovation ecosystem based on a narrative review. This draft report was then provided to the steering committee and regional informants for a merit review (i.e., to discuss the usefulness of how the findings were communicated) prior to finalizing the report. None of the sponsors or key informants played role in drafting, revising or approving the content of this research.

Figure 6 *Schematic of approach taken to develop Progress Report*

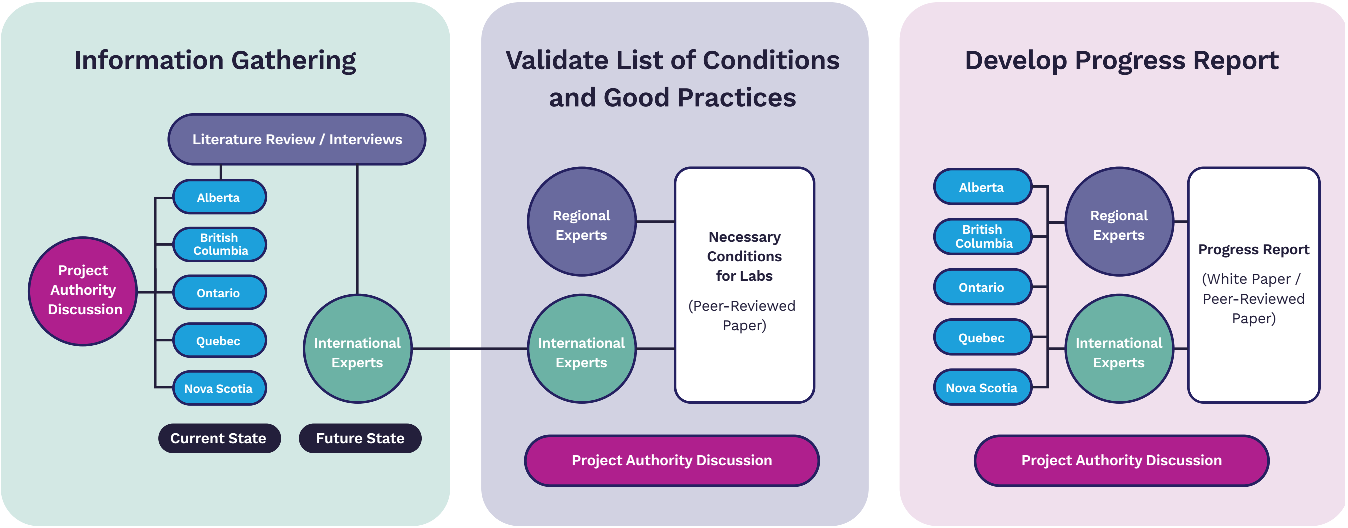

Organization of this Report

The report is organized as follows:

- **Section 1** is background information to help readers understand the purpose of this report
- **Section 2** describes the approach taken to develop the state of readiness Progress Report
- **Section 3** describes current best practices with examples from other major jurisdictions
- **Section 4** describes the current Canadian environment and state of readiness (The Progress Report) for Canada
- **Section 5** describes the humanistic, economic and other broader societal impacts of testing
- **Section 6** provides policy implications and concluding remarks.

Chapter 3.
Best Practices

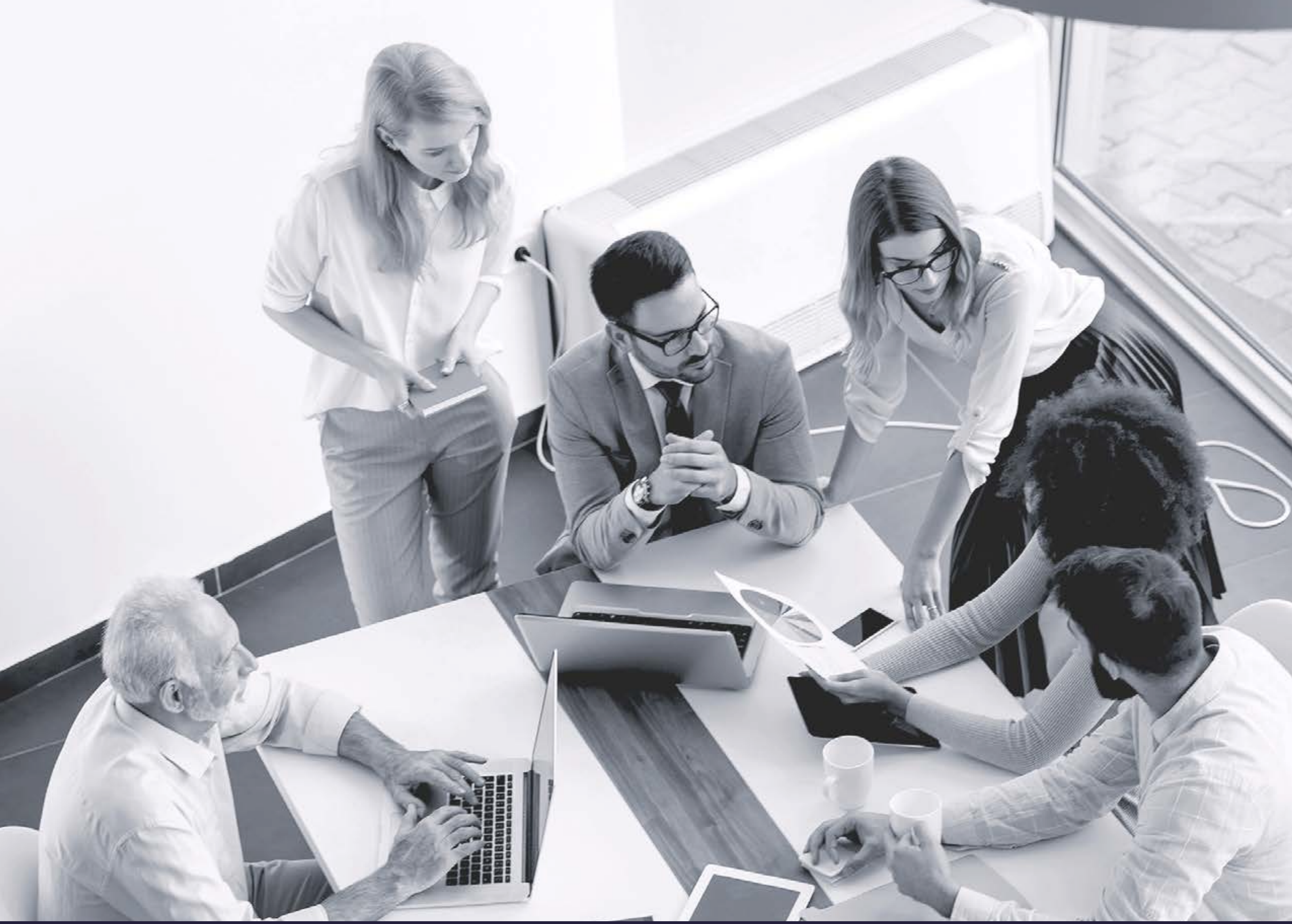

CHAPTER SUMMARY

- Policy conditions for testing have been characterized as being part of three focus area: infrastructure; operations; or health care environment, in general.
- There are good practices within each of the focus areas that reflect the need for regional standards and connectivity; transparent and efficient processes for implementing new technology in a coordinated and equitable fashion with a focus on care navigation.
- Best practices may also require a different approach to the health technology management of new diagnostics including integration of investigational and more established technology; more nimble finance approaches; and regional standards for quality (education, test performance, lab service quality) and data privacy and security.

Introduction

Given the potential for exponential growth of new tests and test approaches, and the complexity of introducing them, health system planners preparing for a future of genomic-based biomarker testing need to grapple with how these services can be delivered effectively and efficiently. As the structure, remit and organization of healthcare systems (and the laboratory functions within them) vary, there are likely to be no one-size-fits-all solution; however , some necessary conditions will be required to manage these technologies in a way that benefits patients and is sustainable

The purpose of this Section is to describe the conditions necessary for policymakers and health system planners to enable a state-of-the-art testing service that includes genome-based testing for acquired and heritable diseases. The list of conditions we propose was developed with an emphasis on describing conditions that would be applicable to any healthcare system, regardless of capacity, organizational structure, financing, population characteristics, standardization of care processes, or underlying culture.[4]

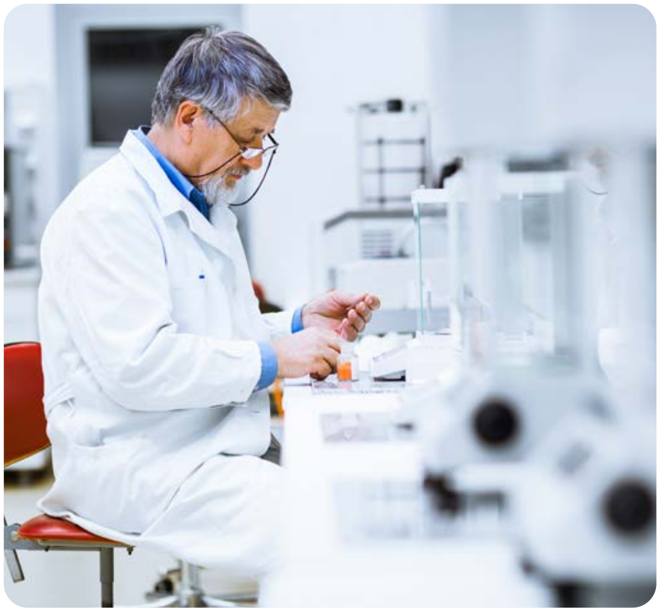

Materials and Methods

The conditions identified in this report were developed throug a mixed methods approach. A narrative literature review was conducted based on a purposive sample of commercially published and grey literature. Searches (See Supplementary Appendix A for search strategy) were performed by a medical librarian specialist and relevant information was identified by a single reviewer (DH) In parallel, conditions were identified using a conventional content approach and based on semi-structured interviews (n = 18; 30–60 min) with key informants and performed from a constructivist point of view. All interviews were conducted by DH with a purposive sample of experts including several of the authors (VM, DMT, DSS, CI, MM, BSS) as well as representatives from pharmaceutical (n = 6) and diagnostic (n = 1) companies. Informants were chosen based on differing expertise and geographic location with some (n = 4) having previously worked with the author.

Interviews were conducted via a recorded video conference call using an interview guide, and all participants approached agreed to be interviewed. Summary notes from transcripts were shared back with participants for verification (member checking). An informal identification of concepts was conducted by one author (DH), and categorized into themes/conditions.

A preliminary list of conditions identified through interview and the literature review was then circulated back to all authors for feedback and a moderated face-to-face discussion. "Effective and efficient" delivery of genome-based diagnostics from testing was defined as one that would most satisfy the "quadruple aim" of reducing per capita costs while improving population health outcomes, patient and care-giver experiences, and provider experiences [6]. These conditions are explained, and grouped according to these aims, and with examples in the next section.

Table 1. *Interviewee characteristics.*

**Participants (n = 18)**	**N, (%)**
Female	6 (33)
Primary role	
Physician/lab leader	4 (22)
Health care administrator	2 (11)
Health services expert/health economist	4 (22)
Patient representative	1 (6)
Private sector representative	7 (39) Work environment
Work Environment	
Public sector	11(61)
Private sector	7 (39)
Location	
Canada	14 (78)
United States	2 (11)
Europe	1 (6)
Other	1 (6)

Results

Interviewees (Table 1) described a number of challenges in achiev-ing the quadruple aim of healthcare within current approached to the implementation and management of genomic testing. These largely related to care interruptions or wait times due to a number of under-lying factors (resources and finance planning; education; informa - ics; and an unclear process for onboarding tests). Other challenges included inappropriate identification of patients and family members; inequitable care delivery; uncoordinated, inconsistent, inappropriate or duplicative care; and inefficient, low-value care. These are grouped by theme in Table 2.

Table 2. *Thematic analysis of challenges identified along with corresponding enabling conditions.*

Quadruple Aim Domain *	Challenge/Theme Identified	Key Informant Quote	Potential Solution/Condition(s) to Help Address
**Work life of care providers**	Care interruptions and wait times	"It is a challenge to connect different streams of planning. A nimble lab service is highly dependent on integrated lab systems and capital planning. Workforce planning and education is also critical."<br><br>"There is a need to triage the urgency based on the test application and clarity about the prerequisite level of evidence to apply."	• Resource planning<br>• Financing approach<br>• Education and training<br>• Informatics<br>• Evaluative function<br>• Entry/exit point for innovation
**Patient and caregiver experiences**	Inappropriate identification of patients and family members	"There is a need for standards around governance, security and patient consent, what we can use the data for etc. Rule around commercial interests in data need to be in place [as well as] some consideration of investing in the laboratory function independently of therapeutic application"	• Service models<br>• Awareness and care navigation
	Inequitable care delivery	"Legislation plays an important role as well. The Acts give different provinces different levels of influence over care coordination	• Regulation
**Health of populations**	Uncoordinated, inconsistent, inappropriate or duplicative care	"There is a need to have clear understanding of what the care pathway is and an aligned community of practice"	• Integration of innovation and healthcare delivery<br>• Creating communities of practice and healthcare system networks
**Per capita costs of healthcare**	Inefficient low-value care	"So then we invest in standards, outcome measures, quality measures etc. along with a process. You can't be too prescriptive because of the wide utility of testing"<br><br>"Information is also valuable and must be valued. Currently information is generated for medico-legal purposes and yet it could be generated to generate revenue and lower care costs"	• Integration of innovation and healthcare delivery

From the challenges identified, an initial list of 14 conditions were identified and pared down to 12. These conditions for testing have been characterized as being part of infrastructure and planning; operations; or the general, health care environment. The sections below provide further elaboration of these, with examples. A summary of conditions, along with a description of issues they are intended to address, and the goals and description of good practice are in Table 3.

Table 3. *Enabling conditions for state-of-the-art delivery of genome-based testing.*

	Issue	Goal	Description of good practice	Policy Example
**Infrastructure**				
**Creating Communities of Practice and Healthcare System Networks**	*Inequitable care delivery*	• Broad stakeholder agreement on appropriate use<br>• Equitable care	• Engagement across all stakeholders	The Australian Genomics Health Alliance, for example, is an attempt to accelerate and evaluate the application of genomic testing in healthcare. It is a "collaborative research partnership across more than 80 diagnostic laboratories, clinical genetics services, and research and academic institutions."[11]
**Resource Planning**	*Care interruptions, wait times or unsustainable care*	• Sustainable care delivery	• Frequent (e.g., 1-3 years) reassessment<br>• Available to all healthcare stakeholders	The US Government Accountability Office conducted a study forecasting a future shortfall of genetic counsellors and medical geneticists in general, and by geographic region.[12]
**Informatics**	*Uncoordinated or duplicative care, inconsistent test development, poor information for evaluation*	• Care coordination<br>• Scientific insight –clinical discovery and health system performance	• Across-region integration<br>• Lab information integrated with electronic health record and healthcare evaluation function	The UK Department of Health & Social Care committed "£4 billion over a five-year period (2016-21) in digital technology, systems and infrastructure, to provide the health and care system with the digital capability and capacity it needs ...."[13]
**Operations**				
**Entry/Exit point for Innovation**	*Technology creep and poorly-performing legacy technology*	• Appropriate health technology management	• Open application and evaluation process<br>• Proposals accepted from all stakeholders<br>• Explicit timelines<br>• Reassessment process	The NHS England, has announced its decision to revisit tests annually, and considering "the co-ordinated replacement of older tests with new and emerging approaches, including considering where evidence still needs to be collected to validate the benefit o moving to [whole-genome sequencing], and identifying where alternative genomic diagnostics, such as gene panels or microarrays, will continue to be needed.[13]
**Evaluative Function**	*Avoid low value care*	• Legitimacy in decision-making<br>• Clear signal for innovators	• Adherence to key principles in health technology assessment including transparency, timeliness and stakeholder engagement[14]<br>• Consistent evaluative framework	An evaluative framework for genetic testing developed for the US Department of Defense recognized the practical need to triage adoption decisions based level on urgency through the use of rapid review and real-world evaluation of new tests.[15]
**Service Models**	*Inequitable and inefficient care*	• Care coordination	• Across-region coordination	NHS England Genomic Laboratory Hubs[16] and US Department of Veteran's Affairs dedicated service centres for testing.[17]
**Awareness and Care navigation**	*Confusion or lack of information regarding test availability*	• Access to care	• Available, up-to-date information of test availability and how to access<br>• Additional supports for care navigation	In France, where testing is more variable across regions, lists of different laboratory sites with contact information are provided.[18]

Table 3. *Cont.*

	Issue	Goal	Description of good practice	Policy Example
**Healthcare Environment**				
**Integration of Innovation and Healthcare Delivery**	***Care lagging behind pace of care innovation and scientific advances***	• Maximize care value	• Private public sector partnerships, and/or<br>• Integration of investigational and established technology	UK and Australian private-public-sector partnerships. [19-20] In Ontario, Canada, reflex testing for newly diagnosed cases of NSCLC (adenocarcinoma/ non-squamous) uses a panel consisting of established and investigational biomarkers[21]
**Financing Approach**	***Care interruptions, wait times or unsustainable care***	• Maximize care value<br>• Access to care<br>• Sustainable care delivery	• Funds available once adoption decision made<br>• Clear value-based, funding formula, amenable to reassessment<br>• Funding for test development, additional human resource costs considered	The US Centers for Medicare and Medicaid Services (CMS) have attempted to incentivize molecular diagnostic innovation by enabling manufacturer-set free pricing for FDA-cleared or approved tests under certain conditions.[22]
**Education and Training**	***Inappropriate care; medical error; care lagging behind pace of care innovation***	• High quality workforce and care delivery	• Training that addresses continuing professional development, knowledge transfer and quality improvement<br>• Across-region educational standards	The Genomics Education Programme (GEP) in England. plans to develop "genomic competencies for specialty training", human resource planning, and providing supports for "curricula development and medical revalidation."[13]
**Regulation**	***Substandard care, negligence and legal liability***	• Minimize preventable harm to individuals from poor test quality	• Regulation that addresses human resource qualifications and training, documentation of records, quality control processes, and proficiency testing.[23-25]<br>• Across-region analytic standards	Regulation is typically addressed through accreditation processes that conform with the International Organization for Standardization (ISO) including ISO 15189 Medical Laboratories. Examples include regulation of clinical genetic testing through CLIA in the US and Canada
**Data privacy and Security**	***Inappropriate identification of patients and family members***	• Minimize preventable harm to individuals and families from testing	• Framework that addresses privacy and security concerns from genetic testing<br>• Across-region privacy standards	The Global Alliance for Genomics & Health, has created a Framework and "Core Elements for Responsible Data Sharing".[26]

Infrastructure and Planning

Creating Communities of Practice and Healthcare System Networks

A well-established network can serve as a basis for deliberation about what tests must have priority, how tests may be valued, what care standards should be in place, what resources will be necessary, how care can be monitored, and other necessary collective judgements that may vary geographically and over time. There will also need to be broad agreement on the use of shared resources, such as biobanks and reporting standards. In regions with more dispersed delivery of care, efforts to create networks may require tiering: first, there is a need to establish across-region consortia to establish wider care standards, shared informational resources, educational standards, and ensure the equitable delivery of services [23]; secondly, there is a need to create strong intraregional consortia for fully integrated delivery of services (i.e., local communities of practice or "collaborative communities" [24]).

Testing is a complex intervention [25] that relies on the timing, expertise, and behaviour across multiple stakeholders for its effective delivery. At its core is a community of practice that includes laboratory leaders and healthcare providers who will have the greatest impact on multidisciplinary decisions in regards to testing, including how and under what conditions a test should be delivered [26]. Broader members of the community are those who will be impacted by the consideration and implementation of new testing. These include the patients, administrators, IT professionals, implementation and genome scientists, public and private sector innovators and others (scientists, legal and ethics experts, professional organizations, bioethicists, regulators). As these groups may not be connected through an organizational structure, strong networks with clear communication between individuals and programs are required for effective implementation and good decision-making [24].

Many international jurisdictions have already established networks through translational research initiatives [27]. In pluralistic or federated health systems such as Sweden and Canada there has been an emphasis on "bottom-up" approaches to creating regional capacity that foster the building up of self-selected organizations aligned with a core set of goals. Some federated jurisdictions, such as Australia, have taken a further "top-down" approach to creating networks after mapping jurisdiction-wide capacity. The Australian Genomics Health Alliance, for example, is an attempt to accelerate and evaluate the application of genomic testing in healthcare. It is a "collaborative research partnership across more than 80 diagnostic laboratories, clinical genetics services, and research and academic institutions" [7].

In more centralized healthcare systems such as England's National Health Service (NHS England), where higher level coordination already exists, the emphasis has been on regional care coordination. Collaboration in England has been facilitated by creating the NHS England Genomic Medicine Service Alliance, an effort to bring together Genomic Laboratory Hubs together with "clinical genetic services inclusive of genomic counsellors, provider organisations across the care continiuum [sic] and with Primary Care Networks, Cancer Alliances, research and academia and patients and public representatives" [28]. In parallel, a consortium of academic researchers (a community of approved researchers with access to the Genomics England Research Environment) was also created as a mechanism of reaping benefits from scientific spillovers from genomic information.

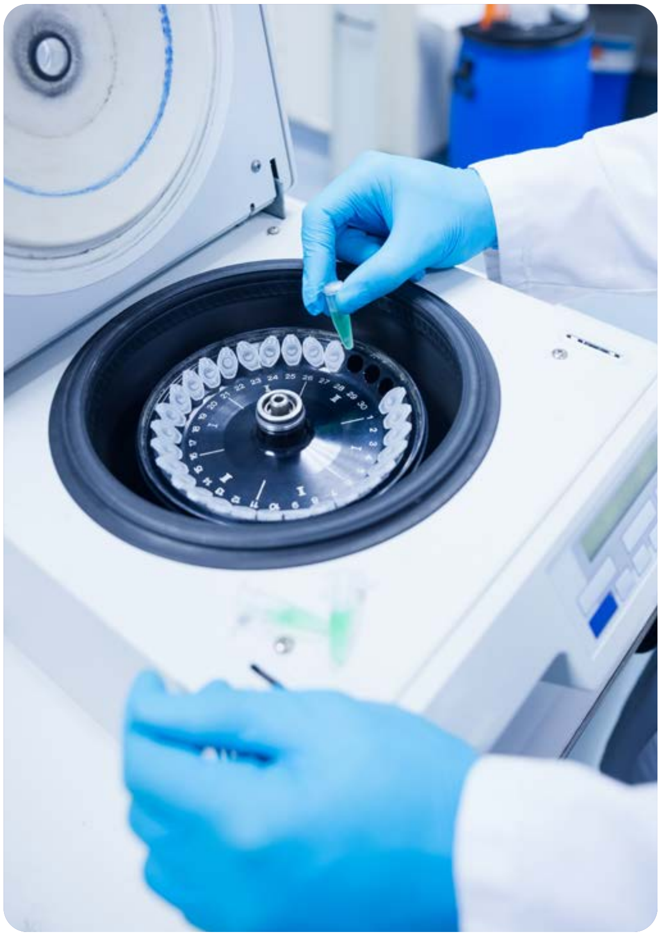

Resource Planning

Resource management and planning for expected impacts on time, people, facilities, equipment, supplies, and information technology is an essential activity in any health system. However, the rapid rate of change of underlying technology and the need for specialized human resources including those involved in tissue sampling (e.g., biopsy), analysis (laboratory technologists/technicians, bioinformatic) and post-test counselling (counsellors and other specialized training) necessitates long-term capital and human resource planning. Workforce planning will need to consider the training and credentialling of highly specialized resources involved with testing. It may also need to consider a plausible range of scenarios of what services are required and the roles and responsibilities of those involved [29,30]. For example, the US Government Accountability Office conducted a study forecasting a future shortfall of genetic counsellors and medical geneticists in general, and by geographic region [8]. Advances in searchable genomic databases to support clinical management, alternative models of service delivery, and centralized delivery of services could greatly reduce the need for these highly specialized human resource requirements in coming years [23,31,32].

Resource planning for the coming era of genomic medicine will require health system planners to revisit traditional funding formulas. Laboratory funding based on volume or a "per-test" approach may not incent its use as it ignores efficiencies that could be realized with changes in approach to testing type (e.g., multigene assay versus single gene approaches [33]), modality (e.g., reflex testing or upfront testing versus ordered testing or sequential testing [34]) or test timing [35,36]. Additional bioinformatics and technologist/technician resources also require consideration. In the UK, for example, the Department of Health & Social Care committed "£4 billion over a five-year period (2016–21) in digital technolog , systems and infrastructure, to provide the health and care system with the digital capability and capacity it needs" [9].

Informatics

Traditionally, laboratory information management emphasizes informatics as a tool for sample tracking and communicating results. For genome-based testing, informatics is also essential for test development, interpretation, and clinical decision support [37,38]. Ensuring adequate integration of test results into electronic health records will also provide a key resource for real-world monitoring, disease management, quality assessment and assurance, and financing [39]. Integration of laboratory information systems with electronic health record systems is also needed to reduce duplication of testing and as a basis for care coordination across health professionals or organizations. An increasingly important aspect of genome-based testing is the ability not only to share information within a healthcare system, but also to share and access data from other informational sources, including external databases, peer-reviewed literature and other healthcare systems.

Histopathology represents a major portion of laboratory medicine and involves images interpreted by human physicians. This area has existed for over a century as an unquantifiable practice within medicine. In the current digital era, digitization of stained images represents a major advance in the practice of personalized medicine. As informatics capability continues to expand, health systems may plan for the integration of digital histomorphologic data and its ongoing analysis into genomics and personalized medicine [40].

Operations

Entry/Exit Point for Innovation

The rate of proposals for new tests and testing modalities necessitates a clear process for the managed adoption and obsolescence of tests [41]. A single point of entry for considering new tests using an application procedure coupled with an evaluation process and formulary is one increasingly used approach that allows multiple stakeholders to engage with the healthcare system [42]. It can also reduce unnecessary testing while providing a strong signal to public and private sector innovators regarding when and under what conditions tests will be adopted [43]. This approach is used in the Canadian provinces of Alberta and Quebec, where an intake form requesting a new test (councils, strategic clinical networks, physicians, patients, innovators or the public) will lead to an evidence-assessment and recommendation before a test is placed on a public formulary.

Given the rate of technological development, healthcare systems will also need to grapple with timeliness, i.e., how long to adopt tests and when tests should be reassessed. New test adoption is a healthcare challenge, as many tests must be considered in the context of other interventions, such as their use as companion diagnostics for new drugs. Any decision to replace or revise an existing test, such as expanding a multigene panel, must consider the balance between patient and healthcare provider unmet need with the inevitable disruption to care protocols, and the speed at which new tests can be replaced. NHS England, for example, has announced its decision to revisit tests annually, and considering the coordinated replacement of older tests with new and emerging approaches, including considering where evidence still needs to be collected to validate the benefit of moving to [whole-genome sequencing], and identifying where alternative genomic diagnostics, such as gene panels or microarrays, will continue to be needed [9].

> “ *The rate of proposals for new tests and testing modalities necessitates a clear process for the managed adoption and obsolescence of tests.* ”

Evaluative Function

Many health systems worldwide have adopted evaluation frameworks for testing based on the analytic validity, clinical validity, clinical utility, and ethical, legal, and social implications (ACCE) approach [44]. While this can be seen as a starting point for evaluation of tests from a clinical standpoint, the ACCE approach does not consider “context-related evaluation dimensions (delivery models, economic evaluation, and organizational aspects)” that will be of interest to policymaking and are a standard part of health technology assessment (HTA) processes [45]. Even using the ACCE framework, it has been argued that ‘clinical utility’ is poorly defined; definitions “may focus on a test’s ability to produce a diagnosis, broader definitions of clinical utility consider health and non-health related, familial and societal outcomes”. Expanded notions of utility, that consider the wider impact of a test result on not only the individual patient, but their families and broader society, may be required to capture benefits to society [46,47].

Many issues related to testing, including availability of evidence and context-driven performance are part of a broader suite of limitations faced by administrators when evaluating diagnostic tests and medical devices [48]. As such, the timing and complexity of traditional approaches to HTA must be balanced against patient need, in an environment where test utility and cost is dynamic. Literature-based approaches to estimating test utility may be limited by enhancements to technology, learning curves [49] or implementation characteristics that affect performance [50]. This strongly suggests the recommended use of both pre-market and post-market data to capture impact of learning curve on outcomes [4] as well as a consideration of costs of implementation when assessing value [51]. Furthermore, and in keeping with key principles for HTA [10], decisions regarding access to testing must be made in a timely manner. Both of these issues are addressed in an evaluative framework for genetic testing developed for the US Department of Defense which recognized the practical need to triage adoption decisions based level on urgency through the use of rapid review and real-world evaluation of new tests [11].

Traditional assumptions and approaches underlying the economic evaluation of decisions for drugs are also challenging to apply [52]. These include the constancy of marginal benefits and costs, and the divisibility of tests provided [53,54]. Payers should expect the marginal costs of adding new tests to a panel or going to a whole exome or genome approach to be quite small relative to other factors such as patient selection, level and type of implementation (education of providers, equipment, geographic distribution) as well as downstream costs (e.g., use of targeted therapies) [55].

Service Models

Like many other forms of production, good practices in organizing health services needs to consider the degree to which the config - ration of delivery is concentrated or dispersed. Dispersed arrangements are more attractive when unit costs do not benefit from eco - omies of scale—such as with primary care and community pharmacy services— and coupled with the need for geographic reach [30,32]. In contrast, economies of scale from advanced testing, coupled with the need for a high degree of standardization and accountability suggest genome-based testing will benefit from a more concentrated model of service delivery.

A further consideration will be to what degree a more concentrated model can be delivered. Service models must first consider care pathways and requisition authority for testing. Models include requisitions by geneticists, primary care practitioners, medical specialists, program-based (such as newborn screening) or direct-to-patient and will depend on the purpose of testing [56]. Genetic testing may be required by independent healthcare programs that are uniquely organized, such as prenatal, pediatric, infectious disease, psychiatry, primary care, and oncology. In some cases, a single test (e.g., BRCA) may be used to assess future risk of disease, prognosis of disease, or predict response to treatment could have different clinical applications, referral pathways, and healthcare system value. Point-of-care testing (POC) technology is also increasingly available, which can expedite decision-making but also challenges a more centralized model of care, and the standardization and accountability that comes with it. POC devices do not negate the need for quality control, external quality assessment, provider training, and data sharing associated with testing. POC tests also further highlight the need for technology adoption decisions that consider an entire community of practice — differences in speed of test results and analytic characteristics will have a downstream impact on patient and healthcare provider experiences.

Care coordination may be greatly facilitated by already-centralized healthcare environments. The NHS England was able to reorganize its existing capacity in 2018, creating a Genomic Medicine Service through its Genomic Laboratory Hubs, each hosted by an acute NHS trust and designated a geographic region for coverage [12]. Similarly, the US Department of Veteran's Affairs has leveraged its existing capacity to deliver genetic testing through its oncology program and dedicated service centres across the US toward non-oncologic indications for testing [13].

In pluralistic or market-based healthcare systems, coordination of care across disparate organizations is facilitated through the use of care standards linked to incentives. Israel, for example, has decided to create regional capacity to deliver comprehensive genomic profiling for non-small cell lung cancer by allowing it separate health management organizations (Kupot Holim) to use their own validated testing approaches, in accordance with specific conditions. Spain, has similarly provided overarching guidance to its autonomous health regions regarding the principles that underly the delivery of genetic tests [57]. Even when designed with best intentions, these approaches may still lead to regional variation and concerns regarding inequity of access. The Spanish Minister of Health has recently announced further efforts will be made to make country-wide access to testing more consistent [58].

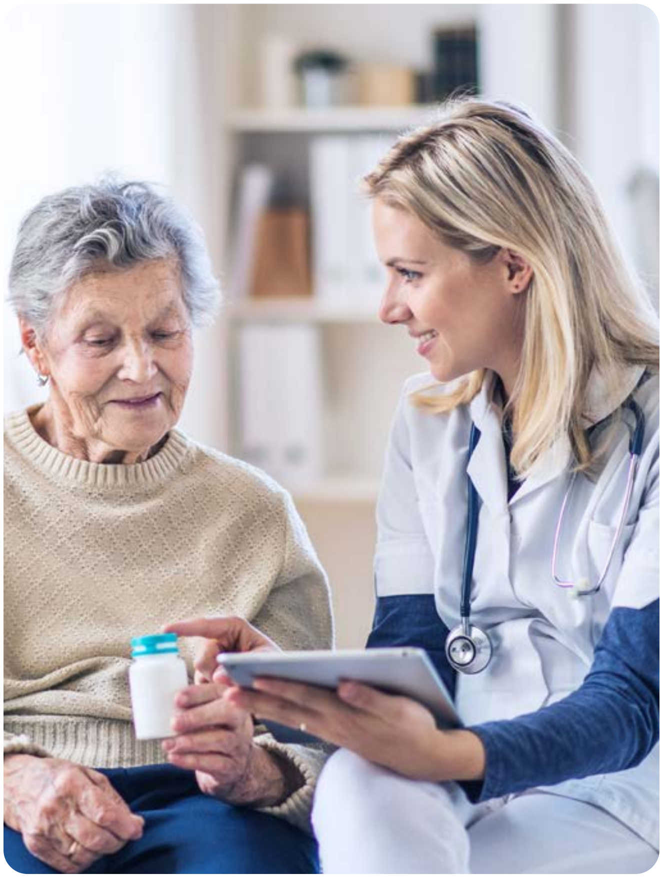

Awareness and Care Navigation

Even with necessary test infrastructure and accessibility, healthcare systems must consider how to communicate to patients and healthcare providers what tests are available and to who and how these are paid for. Published test formularies are a starting point for communicating what tests are available and how they can be accessed. In the Canadian province of Alberta, the addition of tests to a formulary had a stated goal of "streamlining processes by reducing variation in testing and improving healthcare provider and patient access to appropriate, equitable and sustainable laboratory test information [59]". In countries with pluralistic healthcare systems and lacking a common directory, other information can be provided to help care navigation. In France, where testing is more variable across regions, lists of different laboratory sites with contact information are provided [14]. Similarly, the US NIH has developed a test registry, which "contains information about laboratories and the tests they offer but does not contain or gather information on genetic test results" [60].

Healthcare Environment

Integration of Innovation and Healthcare Delivery Functions

Given the rapid future pace of the introduction of new tests, coupled with the potential research benefits associated with testing, healthcare systems will need to consider how the delivery of testing or [/and] scientific discovery alongside testing for healthcare decision-making will be coordinated. This is an inherent challenge with exome- or genome-wide sequencing, which will invariably reveal genetic variants of strong therapeutic, prognostic, or diagnostic significance alongside those lacking evidence [61]. Investigational tests can play an important role in patient care: qualifying patients for clinical trial enrollment, as well as other research endeavors that further understanding of disease. European guidelines have addressed this by suggesting the distinction is clear when reporting results [26]. In Ontario, Canada, for example, reflex testing for newly diagnosed cases of NSCLC (adenocarcinoma/non-squamous) is performed using a panel consisting of more well- established biomarkers. Targeted treatments are currently available for some of the genes tested (e.g., EGFR, ALK, KRAS), but not for others (e.g., FGFR1, SMARCA4, PIK3CA) [17].

Funding tests for "targetable" and "non-targetable" genes together is a pragmatic solution, and is also facilitated by massive parallel ("next-generation") sequencing where additional tests can be added to an assay at negligible cost. It can also allow health systems to revisit testing regimes with less frequency, and avoid significant change management costs. In practice, however, funding both "medically necessary" and "investigational" testing can create a significant conflict for existing insurance frameworks that use evidence and clinical consensus to determine what biomarkers should be funded [62,63]. This challenge led one commentator to ask: "do we redefine [testing] to fit the coverage a evidence framework, or do we redefine the coverage and evidence framework to fit [testing] [63]?

Proposals to change coverage frameworks have been well described and are intended to address payer risk through performance-based payment or coverage with evidence development [63,64]. While a step forward, these solutions may still be difficult to implement in practice, given the inherent limitations of using real-world data to establish the clinical utility of testing [65]. Approaches to circumventing evidence challenges include the use of standardized outcome measures, cascade testing and data sharing through international consortia [66].

A separate solution is to create translational research programs that work in parallel with health systems, or ideally are fully embedded within learning healthcare systems [66]. Many of these already exist today, often facilitated through public-private sector partner- ships, the majority intended to investigate normal genomic variation by sequencing healthy participants (i.e., biobanking) [27]. Some also have stated aims of drug discovery and integrating well-established and emerging tests into regular healthcare system delivery. The Australian government, for example, has created unique partnerships between government, industry, and academia to conduct clinical trials to establish the clinical utility of comprehensive genomic profiling in lung cancer [15], as well as a more recent announcement for rare disease [16].

Financing Approach

The anticipated rate of entry of new tests also requires a nimble financing approach, allowing funds to be released for new tests once decisions to reimburse are made. This may require a shift in thinking for many insurers, who have historically allocated funding for laboratory services on an annual basis based on test volumes [64]. Unlike traditional tests, funding formulas for genetic testing must consider the need for additional human resources associated with development and proficiency testing [67]. Payment models for care may, in turn, drive laboratory utilization and require re-thinking [64,68].

In the US, the Centers for Medicare and Medicaid Services (CMS) have attempted to incentivize molecular diagnostic innovation by enabling manufacturer-set free pricing on the Medicare fee schedule for tests that meet specific Advanced Diagnostic Laboratory est (ADLT) criteria. Qualifying tests must be covered under Medicare, provided by a single laboratory, and either (1) be FDA-cleared or approved, and/or (2) meet three specific criteria ensuring molecular diagnostic innovation [18] Some insurers have also established funding for genetic biomarkers predicated on a "companion" diagnostic paradigm, releasing funds only when companion drugs are approved. Genome-based biomarkers, however, are increasingly used for multiple drugs or therapeutic decisions, including decisions not to use older drugs, and to shorten diagnostic odysseys for conditions with no specific drug therapies [69–72]. In some cases, jurisdictions have additionally relied on pharmaceutical companies or public sector research grants to fund these one-drug, one-test dyads. For public insurers, this inevitably creates a situation where public sector actors are dependent on the private sector (or others) for the delivery of public services, and yet public actors remain accountable to the public at large. This "private finance initiative" type of problem means testing health system priorities are dictated by who is paying, rather than unmet need, equity, or efficiency [73]. In addition to creating structural inefficiency, these arrangements may be disruptive if funds are quickly withdrawn: research grant funding may cease or move away with an investigator; or, a drug company may change its external funding policies. The same company may also reasonably not want to pay for tests that aid competition.

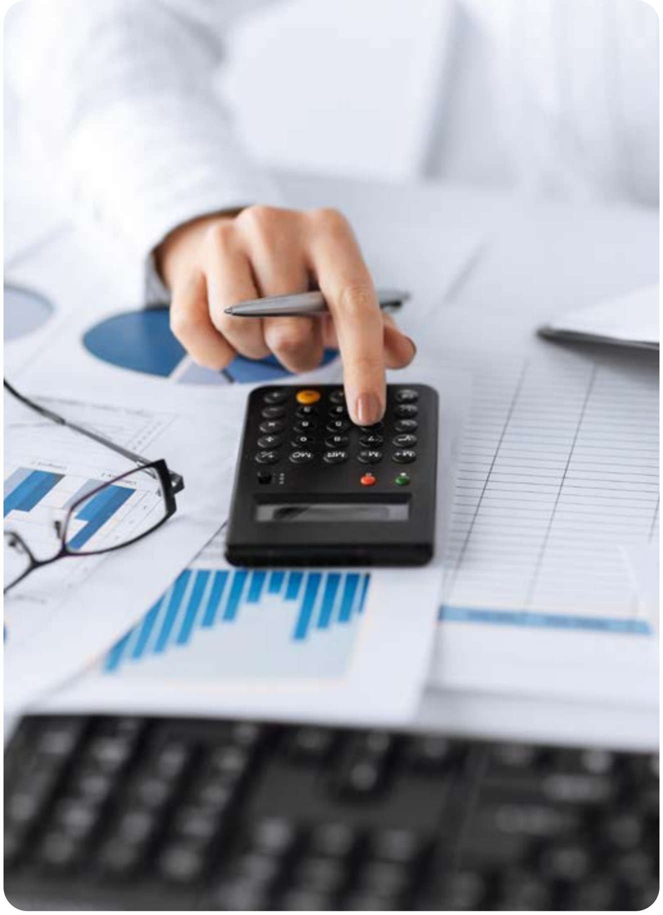

Education and Training

Genetic testing through interprofessional teams distributed across centres and programs introduces new challenges for educating healthcare professionals when creating system-wide changes. Implementation [of] new genome-based tests will change workflow, and necessitates training at the intersection of continuing professional development, knowledge transfer and quality improvement [32,49,74]. This in turn may require new approaches to teaching including work-place-based assessment and in situ simulation that address the many contextual requirements of testing that can ultimately affect test performance, including "coordination of care, tissue procurement and handover, requisition and report design, clear workflow within and between services, automatic information exchange between electronic health systems, and improved communication, with fast feedback loops between health care practitioners [75]".

The need for a significant level of education caused by a significant disruption to organization of services is reflected in the approach proposed by the Genomics Education Programme (GEP) in England. The Programme "routinely engages with the Medical Royal Colleges and actively participates in the NHS England and Academy of Medical Royal Colleges (AoMRC) Genomic Champions Group". Among other areas of focus, the GEP plans to develop "genomic competencies for specialty training", human resource planning, and providing supports for "curricula development and medical revalidation [9]". Funding for each of the Genome Laboratory Hubs also considers the need for education and training. Some jurisdictions have even funded programs aimed at improving genomic knowledge in school-aged children [76].

Regulation

While some health product regulators, such as the FDA, have begun to test claims of clinical validity for commercially available tests, these do not address the multitude of factors that ultimately contribute to test utility and cost-effectiveness. This has heightened the need for effective systems of regulation to address the numerous factors that contribute to test quality, including human resource qualifications and training, documentation of records, quality control processes, and proficiency testing [19–21]. Additional consideration must be given to the training, licensure, registration, and certification of genetic counsellors [77].

Most jurisdictions recognize that advancing the quality of testing requires regulatory standards that involve multiple stakeholders, as it is widely recognized unwanted variation in test performance is largely driven by steps taken before and after analysis [20]. Regulation is typically addressed through accreditation processes that conform with the International Organization for Standardization (ISO) including ISO 15,189 Medical Laboratories. Examples include regulation of clinical genetic testing through CLIA in the US and Canada, and through the National Association of Testing Authorities and the Royal College of Pathologists of Australia (NATA/RCPA) in Australia. In Europe, the CF Network, ERNDIM, GenQA (formally CEQA) and EMQN have more recently harmonized accreditation standards.

Data Privacy and Security

The proper interpretation of disease-gene relationships, particularly for rare variants, require significant amounts of information including family histories and shared information across laboratories, both locally and internationally. The availability of partial or complete genomic information, however, may allow individuals to be identified. Testing also raises ethical issues, such as the duty to warn first-degree relatives who may have a high chance of carrying a disease-causing gene [61]. As such, data requirements associated with genetic testing raise privacy and security concerns that may require revisiting of historical legislation or policies.

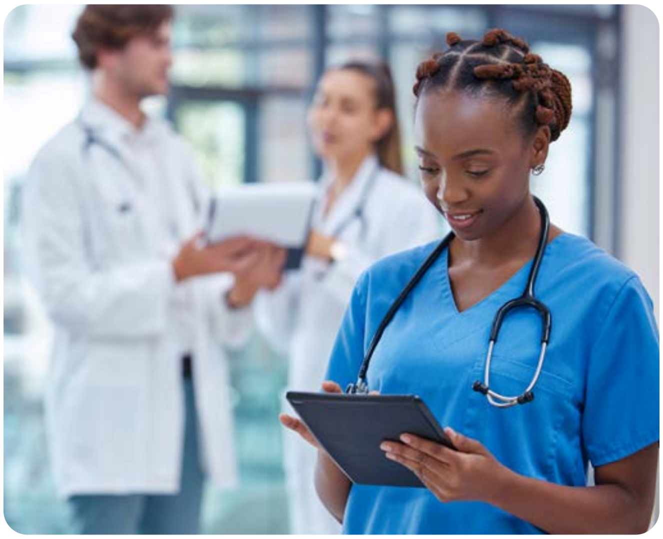

Some of these challenges may be overcome through the adoption of technical solutions and data standards. Technical solutions include privacy-preserving solutions used in information systems, such as blockchain, to help avoid de-identification. Data sharing to community resources, such as the US National Library of Medicine's ClinVar, is also strongly encouraged in international guidelines. Frameworks for data sharing have been bolstered by international efforts, such as the Global Alliance for Genomics & Health, which has created a Framework and "Core Elements for Responsible Data Sharing" [22]. The framework emphasizes maximizing data accessibility of data while minimizing harm to patients and others through a transparent and accountable system.

Data privacy and security concerns may also be addressed through education and training (see Section 3.3.3). A core competency framework developed by NHS Health Education England, for example, identified six areas of proficiency for those responsible for communicating test results. A part of the framework addresses appropriate communication of genomic results, including understanding "the implications of genomic testing for insurance, including the UK Code on Genetic Testing and Insurance" [9].

Some genetic tests may also require outsourcing, due to rarity or health system capacity. Genetic testing opens up the possibility of an output of unprocessed genetic data that requires interpretation from out-of-country providers. In these situations, health system administrators will also need to consider what information can or should lawfully be shared across borders.

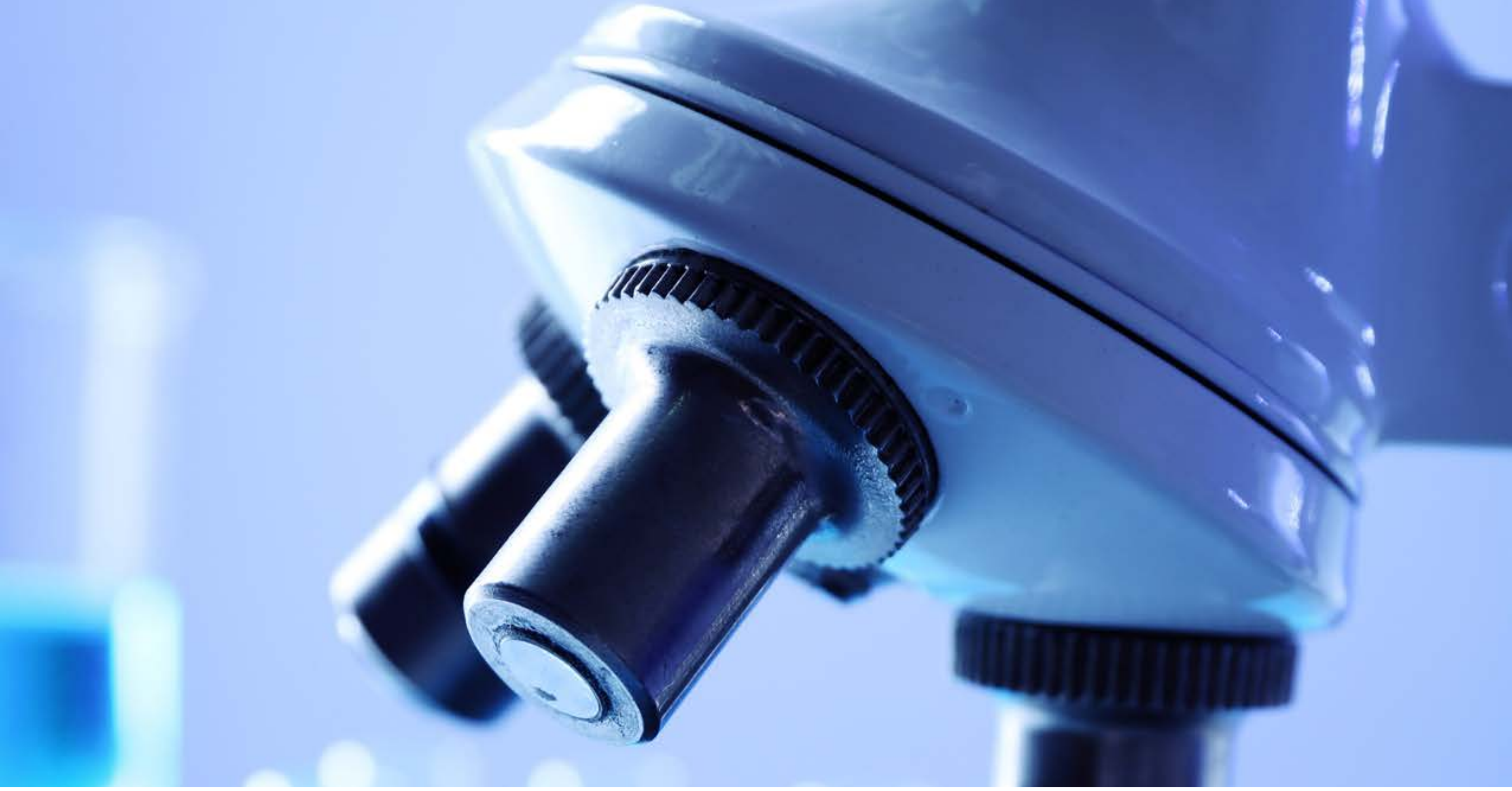

Discussion

Our effort to capture necessary conditions for state-of-the-art genome-based diagnostics service is intended to aid those who must design policies and processes intended to capture the value of genome-based testing. While there is much focus on health technology assessment (HTA) and economic evaluation as a policy response to new technology, we would suggest appropriate management of health technology goes much further than HTA [78]. The conditions listed here reflect broader conditions of high performing health systems that have been previously described [79–82]; these include the need to consider accreditation, regulation, provider training, care coordination, health information technology, evidence-based policy, and financing as a means to reduce inequit , improve care recipient and provider satisfaction, while moderating the rate of expenditure.

While many of the conditions for optimal delivery of care could be applied to other disruptive technologies, the key findings of our review suggest there are some conditions and good practices that will be strongly emphasized in a high-performing genome-based testing service. These include across-region informatics associated with testing, a framework that addresses privacy and security concerns from genetic testing, as well as integration of an innovation and healthcare delivery function through private public sector partnerships or the sanctioned use of investigational technology in mainstream healthcare. Improvements in these areas are significant challenges but necessary ones for a future of learning health systems [83].

Conclusions

We have identified 12 necessary conditions required for policymakers and health care system planners to achieve optimal experiences for care providers, patients and caregiver while achieving better outcomes and minimizing per capita health care costs in the coming era of genomic medicine. As these conditions have been identified through a comprehensive literature review and key informant interviews with international experts, they should be applicable to any healthcare system, regardless of capacity, organizational structure, financing, population characteristics, standardization of care processes, or underlying culture.

These conditions also reflect the multifaceted nature of laboratory technology management as well as the need for additional considerations beyond traditional laboratory technology. As genome-based testing becomes more prevalent in coming years, we hope these conditions and accompanying examples of good practice internationally provide some initial guidance for those who will need to redesign healthcare systems to optimize care.

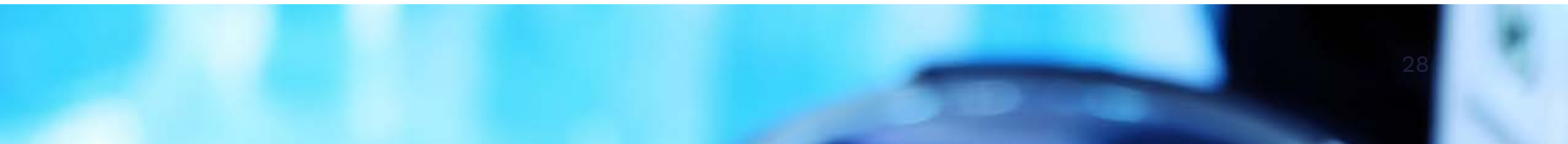

References

1. Califf, R.M. Biomarker definitions and their applications. *Exp. Biol. Med.* **2018**, 243, 213–221. [CrossRef] [PubMed]
2. Lionel, A.C.; Costain, G.; Monfared, N.; Walker, S.; Reuter, M.S.; Hosseini, S.M.; Thiruvahindrapuram, B.; Merico, D.; Jobling, R.; Nalpathamkalam, T.; et al. Improved Diagnostic Yield Compared with Targeted Gene Sequencing Panels Suggests a Role for Whole-Genome Sequencing as a First-Tier Genetic Test. *Genet. Med*. **2018**, 20, 435–443.
3. Tran, D.T.; Akpinar, I.; Jacobs, P. The Costs of Industry-Sponsored Drug Trials in Canada. *PharmacoEconomics* **2020,** *4*, 353–359. [CrossRef]
4. Polisena, J.; Castaldo, R.; Ciani, O.; Federici, C.; Borsci, S.; Ritrovato, M.; Clark, D.; Pecchia, L. Health Technology Assessment Methods Guidelines For Medical Devices: How Can We Address The Gaps? The International Federation Of Medical And Biological Engineering Perspective. *Int. J. Technol. Assess. Health Care* **2018**, *34*, 276–289.
5. Piña, I.L.; Cohen, P.D.; Larson, D.B.; Marion, L.N.; Sills, M.R.; Solberg, L.I.; Zerzan, J. A Framework for Describing Health Care Delivery Organizations and Systems. *Am. J. Public Health* **2015**, *105*, 670–679.
6. Sikka, R.; Morath, J.M.; Leape, L. The Quadruple Aim: Care, Health, Cost and Meaning in Work. *BMJ Qual. Saf.* **2015**, *24*, 608–610.
7. Stark, Z.; Boughtwood, T.; Phillips, P.; Christodoulou, J.; Hansen, D.P.; Braithwaite, J.; Newson, A.J.; Gaff, C.L.; Sinclair, A.H.; North, K.N. Australian Genomics: A Federated Model for Integrating Genomics into Healthcare. *Am. J. Hum. Genet.* **2019**, *105*, 7–14.
8. U.S. Government Accountability Office. Genetic Services: Information on Genetic Counselor and Medical Geneticist orkforces. Available online: https://www.gao.gov/products/gao-20-593 (accessed on 5 August 2022).
9. Department of Health & Social Care. *Government Response to the House of Commons Science and Technology Committee Third Report of Session 2017-19: "Genomics and Genome Editing in the NHS";* Department of Health & Social Care: London, UK, 2018; ISBN 978-1-5286-0631-8.
10. Drummond, M.F.; Schwartz, J.S.; Jönsson, B.; Luce, B.R.; Neumann, P.J.; Siebert, U.; Sullivan, S.D. Key Principles for the Improved Conduct of Health Technology Assessments for Resource Allocation Decisions. *Int. J. Technol. Assess. Health Care* **2008,** *24*, 244–258.
11. National Academies of Sciences, Engineering, and Medicine; Health and Medicine Division; Board on Health Care Services; Board on the Health of Select Populations; Committee on the Evidence Base for Genetic Testing. *An Evidence Framework for Genetic Testing*; National Academies Press (US): Washington, DC, USA, 2017; ISBN 978-0-309-45329-5.
12. Barwell, J.; Snape, K.; Wedderburn, S. The New Genomic Medicine Service and Implications for Patients. *Clin. Med.* **2019,** *19*, 273–277. [CrossRef]
13. Dong, O.M.; Bates, J.; Chanfreau-Coffinie , C.; Naglich, M.; Kelley, M.J.; Meyer, L.J.; Icardi, M.; Vassy, J.L.; Sriram, P.; Heise, C.W.; et al. Veterans Affairs Pharmacogenomic Testing for Veterans (PHASER) Clinical Program. *Pharmacogenomics* **2021,** *22*, 137–144.
14. Le Dispositif National d'oncogénétique—Oncogénétique et Plateformes de Génétique Moléculaire. Available online: https://www.e-cancer.fr/Professionnels-de-sante/L-organisation-de-l-offre-de-soins/Oncogenetique-et-plateformes-de-genetique-moleculaire/Le-dispositif-national-d-oncogenetique#toc-organisation (accessed on 27 June 2022).
15. Australian Clinical Trials. Available online: https://www.australianclinicaltrials.gov.au/anzctr/trial/ACTRN12621000221853 (accessed on 27 June 2022).
16. Garvan Institute of Medical Research. $185 Million Investment to Fast-Track Treatments for Rare and 'Untreatable' Cancers. Available online: https://www.garvan.org.au/news-events/news/185-million-investment-to-fast-track-treatments-for-rare- and-2018untreatable2019-cancers (accessed on 27 June 2022).
17. Canadian Cancer Survivor Network. Comprehensive Cancer Testing in Ontario—Wave 1. Available online: https://survivornet. ca/news/comprehensive-cancer-testing-in-ontario-wave-1/ (accessed on 27 June 2022).
18. CMS. Medicare Part B Clinical Laboratory Fee Schedule Guidance for Laboratories on Advanced Diagnostic Laboratory Tests. 2018. Available online: https://www.cms.gov/Medicare/Medicare-Fee-for-Service-Payment/ClinicalLabFeeSched/Downloads/Guidance-for-Labora-tories-on-ADLTs.pdf (accessed on 27 June 2022).
19. Nelson, B. Ensuring Quality in Genomic Medicine: Amid the Rise in Complex Laboratory-Developed Tests, Regulatory Officials Are Seeking the Right Balance on Quality Assurance. *Cancer Cytopathol.* **2014**, *122*, 855–856.
20. Meier, F.A.; Badrick, T.C.; Sikaris, K.A. What's to Be Done About Laboratory Quality? Process Indicators, Laboratory Stewardship, the Out-comes Problem, Risk Assessment, and Economic Value: Responding to Contemporary Global Challenges. *Am. J. Clin. Pathol.* **2018**, *149*, 186–196.
21. Evans, B.J.; Javitt, G.; Hall, R.; Robertson, M.; Ossorio, P.; Wolf, S.M.; Morgan, T.; Clayton, E.W. How Can Law and Policy Advance Quality in Genomic Analysis and Interpretation for Clinical Care? *J. Law Med. Ethics* **2020**, *48*, 44–68.
22. Framework for Responsible Sharing of Genomic and Health-Related Data. Available online: https://www.ga4gh.org/genomic- data-toolkit/regulatory-ethics-toolkit/framework-for-responsible-sharing-of-genomic-and-health-related-data/ (accessed on 15 June 2022).
23. Huser, V.; Sincan, M.; Cimino, J.J. Developing Genomic Knowledge Bases and Databases to Support Clinical Management: Current Perspectives. *Pharmgenomics Pers. Med*. **2014**, *7*, 275–283.
24. FDA. Collaborative Communities: Addressing Health Care Challenges Together. 2021. Available online: https://www.fda.gov/about-fda/cdrh-strategic-priorities-and-updates/collaborative-communities-addressing-health-care-challenges-together (accessed on 23 June 2022).
25. Skivington, K.; Matthews, L.; Simpson, S.A.; Craig, P.; Baird, J.; Blazeby, J.M.; Boyd, K.A.; Craig, N.; French, D.P.; McIntosh, E.; et al. A New Framework for Developing and Evaluating Complex Interventions: Update of Medical Research Council Guidance. *BMJ* **2021**, *374*, n2061.
26. Matthijs, G.; Souche, E.; Alders, M.; Corveleyn, A.; Eck, S.; Feenstra, I.; Race, V.; Sistermans, E.; Sturm, M.; Weiss, M.; et al. Guidelines for Diagnostic Next-Generation Sequencing. *Eur. J. Hum. Genet***.** **2016**, *24,* 2–5.
27. Kovanda, A.; Zimani, A.N.; Peterlin, B. How to Design a National Genomic Project-A Systematic Review of Active Projects. *Hum. Genom.* **2021**, *15*, 20.
28. NHS England. NHS Genomic Medicine Service Alliances to Help Embed Genomics into Patient Care Pathways. Available online: https://www.england.nhs.uk/blog/nhs-genomic-medicine-service-alliances-to-help-embed-genomics-into-patient-care-pathways/ (accessed on 16 June 2022).
29. Houwink, E.J.F. Comment on Future Trends in Clinical Genetic and Genomic Services by Borle et Al. *Eur. J. Hum. Genet.* **2022,** *30*, 505–506.
30. Raspa, M.; Moultrie, R.; Toth, D.; Haque, S.N. Barriers and Facilitators to Genetic Service Delivery Models: Scoping Review. *Interact. J. Med. Res*. **2021**, *10*, e23523.
31. Stoll, K.; Kubendran, S.; Cohen, S.A. The Past, Present and Future of Service Delivery in Genetic Counseling: Keeping up in the Era of Precision Medicine. Am. J. Med. Genet. Part C Semin. Med. Genet. 2018, 178, 24–37

32. Chou, A.F.; Duncan, A.R.; Hallford, G.; Kelley, D.M.; Dean, L.W. Barriers and Strategies to Integrate Medical Genetics and Primary Care in Underserved Populations: A Scoping Review. J. Community Genet. 2021, 12, 291–309.
33. Steuten, L.; Goulart, B.; Meropol, N.J.; Pritchard, D.; Ramsey, S.D. Cost Effectiveness of Multigene Panel Sequencing for Patients With Advanced Non-Small-Cell Lung Cancer. *JCO Clin. Cancer Inform.* **2019**, *3*, 1–10.
34. VandenBussche, C.J.; Cimino-Mathews, A.; Park, B.H.; Emens, L.A.; Tsangaris, T.N.; Argani, P. Reflex Estrogen Recep- tor/Progesterone Rece - tor/Human Epidermal Growth Factor Receptor 2 (ER/PR/Her2) Analysis of Breast Cancers in Needle Core Biopsy Specimens Dramatically Increases Health Care Costs. *Am. J. Surg. Pathol.* **2015**, *39*, 939–947.
35. El Karak, F.; Rassy, E.; Bassil, J.; Awali, M.; Hanna, C.; Saroufim, A.; Nas , F.; Farhat, F.; Kattan, J.; Ghosn, M.; et al. Impact and Cost-Effectiveness of Oncotype DX for Guiding Adjuvant Chemotherapy Decisions in Early Breast Cancer. *Gulf. J. Oncol.* **2021**, *1*, 27–35.
36. Snowsill, T.M.; Ryan, N.A.J.; Crosbie, E.J.; Frayling, I.M.; Evans, D.G.; Hyde, C.J. Cost-Effectiveness Analysis of Reflex esting for Lynch Syndrome in Women with Endometrial Cancer in the UK Setting. *PLoS ONE* **2019**, *14,* e0221419. [CrossRef] [PubMed]
37. Louie, B.; Mork, P.; Martin-Sanchez, F.; Halevy, A.; Tarczy-Hornoch, P. Data Integration and Genomic Medicine. *J. Biomed. Inform.* **2007**, *40*, 5–16.
38. Shah, S. Better Bioinformatics Will Help Labs Manage Genetic Testing. *MLO Med. Lab. Obs.* **2017**, *49,* 31.
39. Warner, J.L.; Jain, S.K.; Levy, M.A. Integrating Cancer Genomic Data into Electronic Health Records. *Genome Med*. **2016**, *8*, 113.
40. Cohen, S.; Levenson, R.; Pantanowitz, L. Artificial Intelligence in Patholog . *Am. J. Pathol.* **2021**, *191*, 1670–1672.
41. Kirwin, E.; Round, J.; Bond, K.; McCabe, C. A Conceptual Framework for Life-Cycle Health Technology Assessment. *Value Health* **2022**, *25*, 1116–1123.
42. Jackson, B.R. Laboratory Formularies. Clin. Chim. *Acta* **2014**, *427*, 151–153.
43. Warren, J.S. Laboratory Test Utilization Program: Structure and Impact in a Large Academic Medical Center. *Am. J. Clin. Pathol.* **2013**, *139*, 289–297.
44. Pitini, E.; De Vito, C.; Marzuillo, C.; D'Andrea, E.; Rosso, A.; Federici, A.; Di Maria, E.; Villari, P. How Is Genetic Testing Evaluated? A Systematic Review of the Literature. *Eur. J. Hum. Genet.* **2018**, *26*, 605–615.
45. Kristensen, F.B.; Husereau, D.; Huic´, M.; Drummond, M.; Berger, M.L.; Bond, K.; Augustovski, F.; Booth, A.; Bridges, J.F.P.; Grimshaw, J.; et al. Identifying the Need for Good Practices in Health Technology Assessment: Summary of the ISPOR HTA Council Working Group Report on Good Practices in HTA. *Value Health* **2019**, *22*, 13–20.
46. ACMG Board of Directors. Clinical Utility of Genetic and Genomic Services: A Position Statement of the American College of Medical Genetics and Genomics. *Genet. Med*. 2**015**, *17*, 505–507.
47. Wurcel, V.; Cicchetti, A.; Garrison, L.; Kip, M.M.A.; Koffijberg, H.; Kolbe, A.; Leeflang, M.M.G.; Merlin, .; Mestre-Ferrandiz, J.; Oortwijn, W.; et al. The Value of Diagnostic Information in Personalised Healthcare: A Comprehensive Concept to Facilitate Bringing This Technology into Healthcare Systems. *Public Health Genom.* **2019,** *22,* 8–15.
48. Bouttell, J.; Heggie, R.; Oien, K.; Romaniuk, A.; VanSteenhouse, H.; von Delft, S.; Hawkins, N. Economic Evaluation of Genomic/Genetic Tests: A Review and Future Directions. *Int. J. Technol. Assess. Health Care* **2022**, *38,* e67.
49. Corominas, J.; Smeekens, S.P.; Nelen, M.R.; Yntema, H.G.; Kamsteeg, E.-J.; Pfundt, R.; Gilissen, C. Clinical Exome Sequencing- Mistakes and Caveats. *Hum. Mutat*. **2022,** *43*, 1041–1055.
50. Pollard, S.; Weymann, D.; Chan, B.; Ehman, M.; Wordsworth, S.; Buchanan, J.; Hanna, T.P.; Ho, C.; Lim, H.J.; Lorgelly, P.K.; et al. Defining a Core Data Set for the Economic Evaluation of Precision Oncology. *Value Health* **2022**, *25,* 1371–1380.
51. Hoomans, T.; Fenwick, E.A.L.; Palmer, S.; Claxton, K. Value of Information and Value of Implementation: Application of an Analytic Framework to Inform Resource Allocation Decisions in Metastatic Hormone-Refractory Prostate Cancer. *Value Health* **2009**, *12*, 315–324.
52. Tarride, J.-E.; Gould, T.; Thomas, D.M. Challenges of Conducting Value Assessment for Comprehensive Genomic Profiling *Int. J. Technol. Assess. Health Care* **2022**, *38*, e57.
53. Antonanzas, F.; Juarez-Castello, C.A.; Rodriguez-Ibeas, R. Some Economics on Personalized and Predictive Medicine. *Eur. J. Health Econ.* **2015**, *16*, 985–994.
54. Wright, S.J.; Paulden, M.; Payne, K. Implementing Interventions with Varying Marginal Cost-Effectiveness: An Application in Precision Medicine. *Med. Decis. Mak.* **2020**, *40*, 924–938.
55. Sabatini, L.M.; Mathews, C.; Ptak, D.; Doshi, S.; Tynan, K.; Hegde, M.R.; Burke, T.L.; Bossler, A.D. Genomic Sequencing Procedure Microcosting Analysis and Health Economic Cost-Impact Analysis: A Report of the Association for Molecular Pathology. *J. Mol. Diagn.* **2016**, *18*, 319–328.
56. Unim, B.; Pitini, E.; Lagerberg, T.; Adamo, G.; De Vito, C.; Marzuillo, C.; Villari, P. Current Genetic Service Delivery Models for the Provision of Genetic Testing in Europe: A Systematic Review of the Literature. *Front. Genet.* **2019**, *10*, 552.
57. Spain Ministry of Health. [Common Portfolio Of Services: Genetics]. Available online: https://www.sanidad.gob.es/ca/profesionales/prestaciones-Sanitarias/CarteraDeServicios/docs/Genetica.pdf (accessed on 24 June 2022).
58. La Moncloa. Sanidad Avanza Hacia la Homogenización del Acceso a las Pruebas Genéticas en Todo el Territorio para el Diagnóstico, Tratamiento y Seguimiento de las Enfermedades Rara [Prensa/Actualidad/Sanidad]. 12 May 2022. Available online: https://www.lamoncloa.gob.es/serviciosde-prensa/notasprensa/sanidad14/Paginas/2022/120522-darias-enfermedades-raras.aspx (accessed on 24 June 2022).
59. Alberta Precision Laboratories. Lab Formulary Intake Request. Available online: https://www.albertaprecisionlabs.ca/tc/Page13931.aspx (accessed on 27 June 2022).
60. Rubinstein, W.S.; Maglott, D.R.; Lee, J.M.; Kattman, B.L.; Malheiro, A.J.; Ovetsky, M.; Hem, V.; Gorelenkov, V.; Song, G.; Wallin, C.; et al. The NIH Genetic Testing Registry: A New, Centralized Database of Genetic Tests to Enable Access to Comprehensive Information and Improve Transparency. *Nucleic Acids Res.* **2013**, *41*, D925–D935.
61. Manolio, T.A.; Rowley, R.; Williams, M.S.; Roden, D.; Ginsburg, G.S.; Bult, C.; Chisholm, R.L.; Deverka, P.A.; McLeod, H.L.; Mensah, G.A.; et al. Opportunities, Resources, and Techniques for Implementing Genomics in Clinical Care. *Lancet* **2019**, *394*, 511–520.
62. Trosman, J.R.; Weldon, C.B.; Gradishar, W.J.; Benson, A.B.; Cristofanilli, M.; Kurian, A.W.; Ford, J.M.; Balch, A.; Watkins, J.; Phillips, K.A. From the Past to the Present: Insurer Coverage Frameworks for Next-Generation Tumor Sequencing. *Value Health* **2018**, *21*, 1062–1068.
63. Trosman, J.R.; Weldon, C.B.; Kelley, R.K.; Phillips, K.A. Challenges of Coverage Policy Development for Next-Generation Tumor Sequencing Pan-els: Experts and Payers Weigh In. J. Natl. Compr. Cancer Netw. 2015, 13, 311–318.

64. Koleva-Kolarova, R.; Buchanan, J.; Vellekoop, H.; Huygens, S.; Versteegh, M.; Mölken, M.R.; Szilberhorn, L.; Zelei, T.; Nagy, B.; Wordsworth, S.; et al. Financing and Reimbursement Models for Personalised Medicine: A Systematic Review to Identify Current Models and Future Options. *Appl. Health Econ. Health Policy* **2022**, *20,* 501–524.
65. Donoghue, M.T.A.; Schram, A.M.; Hyman, D.M.; Taylor, B.S. Discovery through Clinical Sequencing in Oncology. *Nat Cancer* **2020**, *1*, 774–783.
66. Peterson, J.F.; Roden, D.M.; Orlando, L.A.; Ramirez, A.H.; Mensah, G.A.; Williams, M.S. Building Evidence and Measuring Clinical Outcomes for Genomic Medicine. *Lancet* **2019**, *394*, 604–610.
67. Christensen, K.D.; Phillips, K.A.; Green, R.C.; Dukhovny, D. Cost Analyses of Genomic Sequencing—Lessons Learned from the MedSeq Project. *Value Health* **2018**, *21*, 1054–1061.
68. Chami, N.; Sweetman, A. Payment Models in Primary Health Care: A Driver of the Quantity and Quality of Medical Laboratory Utilization. *Health Econ.* **2019**, *28,* 1166–1178.
69. Aberuyi, N.; Rahgozar, S.; Ghodousi, E.S.; Ghaedi, K. Drug Resistance Biomarkers and Their Clinical Applications in Childhood Acute Lymphoblastic Leukemia. *Front. Oncol*. **2020**, *9*, 1496.
70. Guo, C.; Song, C.; Zhang, J.; Gao, Y.; Qi, Y.; Zhao, Z.; Yuan, C. Revisiting Chemoresistance in Ovarian Cancer: Mechanism, Biomarkers, and Precision Medicine. *Genes Dis*. **2022**, *9*, 668–681.
71. Mao, C.-X.; Li, M.; Zhang, W.; Zhou, H.-H.; Yin, J.-Y.; Liu, Z.-Q. Pharmacogenomics for the Efficacy of Platinum-Based Chemotherapy: Old Drugs, New Integrated Perspective. *Biomed. Pharmacother*. **2020**, *126*, 110057.
72. Pénzváltó, Z.; Lánczky, A.; Lénárt, J.; Meggyesházi, N.; Krenács, T.; Szoboszlai, N.; Denkert, C.; Pete, I.; Gyo˝rffy, B. MEK1 Is Associated with Carboplatin Resistance and Is a Prognostic Biomarker in Epithelial Ovarian Cancer. *BMC Cancer* **2014**, *14*, 837.
73. Greer, S.L.; Wismar, M.; Figueras, J. (Eds.) *Strengthening Health System Governance: Better Policies, Stronger Performance;* European Observatory on Health Systems and Policies Series; Open University Press: Maidenhead, UK, 2016; ISBN 978-0-335-26134-5.
74. Kitto, S. Opening up the CPD Imagination. *J. Contin. Educ. Health Prof.* **2019**, *39*, 159–160.
75. Cheema, P.K.; Gomes, M.; Banerji, S.; Joubert, P.; Leighl, N.B.; Melosky, B.; Sheffield, B.S.; Stockle , T.; Ionescu, D.N. Consensus Recommendations for Optimizing Biomarker Testing to Identify and Treat Advanced EGFR-Mutated Non-Small-Cell Lung Cancer. *Curr. Oncol.* **2020**, *27*, 321–329.
76. Mitropoulos, K.; Cooper, D.N.; Mitropoulou, C.; Agathos, S.; Reichardt, J.K.V.; Al-Maskari, F.; Chantratita, W.; Wonkam, A.; Dandara, C.; Katsila, T.; et al. Genomic Medicine Without Borders: Which Strategies Should Developing Countries Employ to Invest in Precision Medicine? A New "Fast-Second Winner" Strategy. *OMICS J. Integr. Biol*. **2017**, *21*, 647–657.
77. Abacan, M.; Alsubaie, L.; Barlow-Stewart, K.; Caanen, B.; Cordier, C.; Courtney, E.; Davoine, E.; Edwards, J.; Elackatt, N.J.; Gardiner, K.; et al. The Global State of the Genetic Counseling Profession. *Eur. J. Hum. Genet*. **2019**, *27*, 183–197.
78. Fulop, N.; Allen, P.; Clarke, A.; Black, N. From Health Technology Assessment to Research on the Organisation and Delivery of Health Services: Addressing the Balance. *Health Policy* **2003**, *63*, 155–165.
79. Pursuing Perfection: Raising the Bar for Health Care Performance. Available online: http://www.rwjf.org/en/library/research/2011/12/pursuing-perfection.html (accessed on 10 August 2022).
80. Organizing the, U.S. Health Care Delivery System for High Performance. Available online: http://www.commonwealthfund.org/publications/fund-reports/2008/aug/organizing-us-health-care-delivery-system-high-performance (accessed on 10 Au- gust 2022).
81. Docteur, E. Towards High-Performing Health Systems: The OECD Health Project; Organisation for Economic Co-Operation and Development: Paris, France, 2004. Available online: https://www.oecd.org/els/health-systems/31785551.pdf (accessed on 27 June 2022).
82. Ahluwalia, S.C.; Damberg, C.L.; Silverman, M.; Motala, A.; Shekelle, P.G. What Defines a High-Performing Health Care Delivery System: A Sy - tematic Review. Jt. Comm. *J. Qual. Patient Saf.* **2017**, *43,* 450–459.
83. Campbell, D. Reforms as Experiments. *Am. Psychol.* **1969**, *24*, 409–429.

Chapter 4.
Current Canadian Environment & State of Readiness in Canada

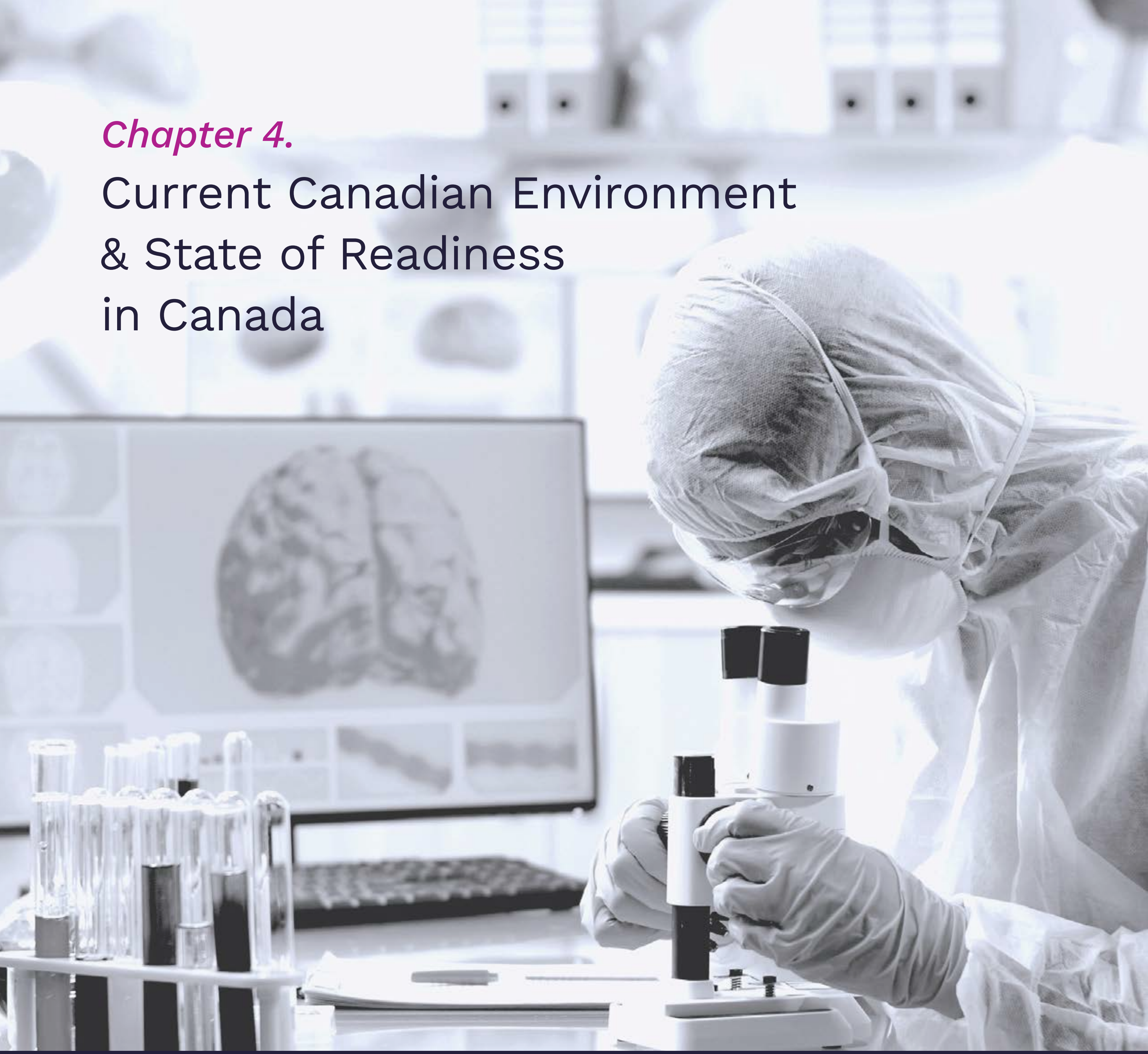

CHAPTER SUMMARY

- Canada's State of Readiness Progress Report for genome-based testing examines the state of readiness for testing across 5 healthcare regions in Canada: Ontario, Quebec, British Columbia, Alberta, and Nova Scotia.
- The Progress Report was developed by comparing optimal conditions for a genome-based service (Section 3) with current practice in each province.
- Overall, Canada is partially ready for a future of genomic medicine but making progress. Canada's largest healthcare regions are in varying states of readiness for genomic testing; Alberta and Quebec appear to be most established but each province has identifiable needs for improvement.

The State of Readiness Progress Report uses a purposive sample of 5 healthcare regions in Canada: Ontario, Quebec, British Columbia, Alberta, and Nova Scotia. The first four regions were chosen as they are the most populous regions in Canada, and represent ~75% of the Canadian population. While Nova Scotia is Canada's 7th most populous region, it was chosen as it has the largest population in Eastern Canada. Each province is compared against the enabling conditions presented in Chapter 3, with the exception of data privacy conditions.

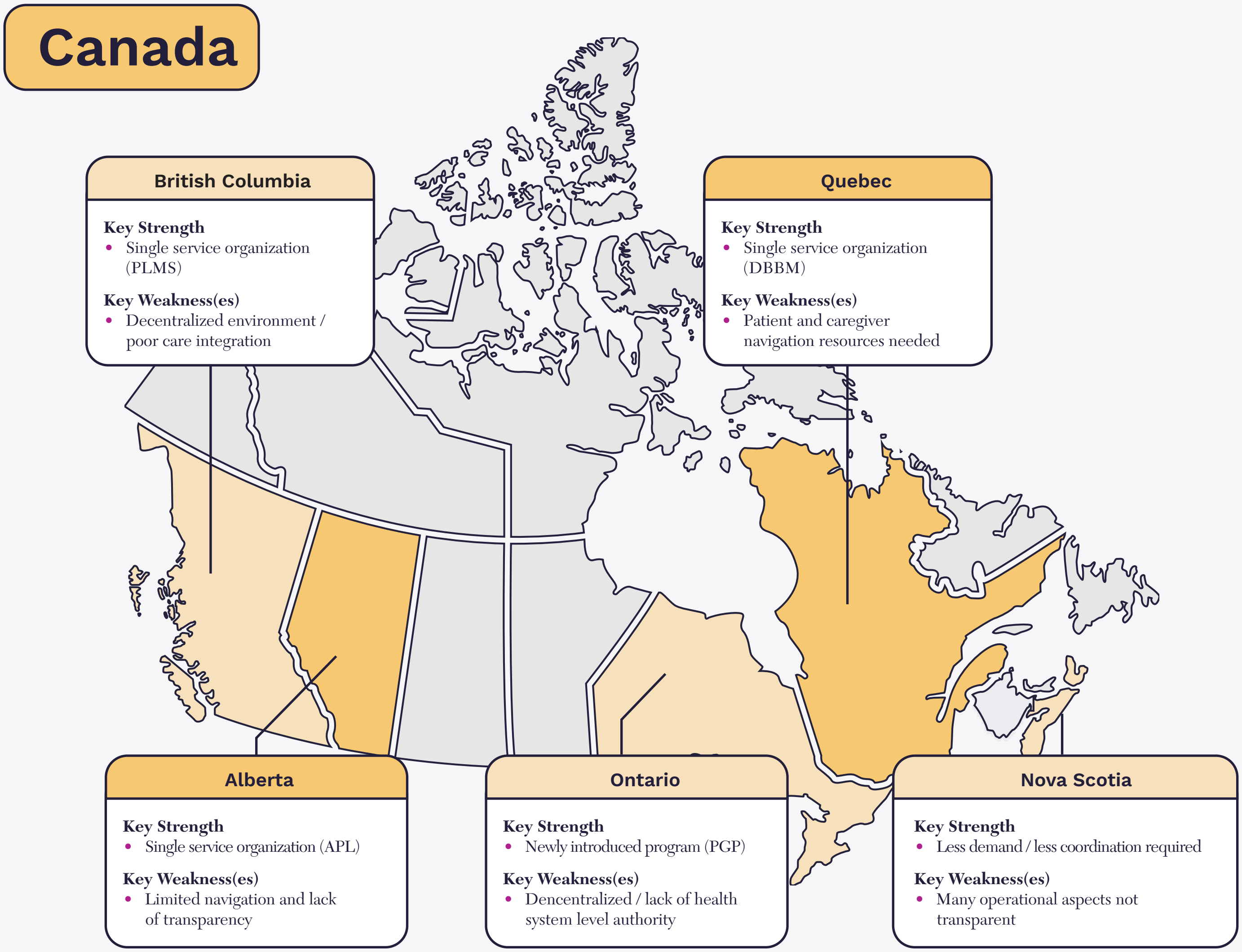

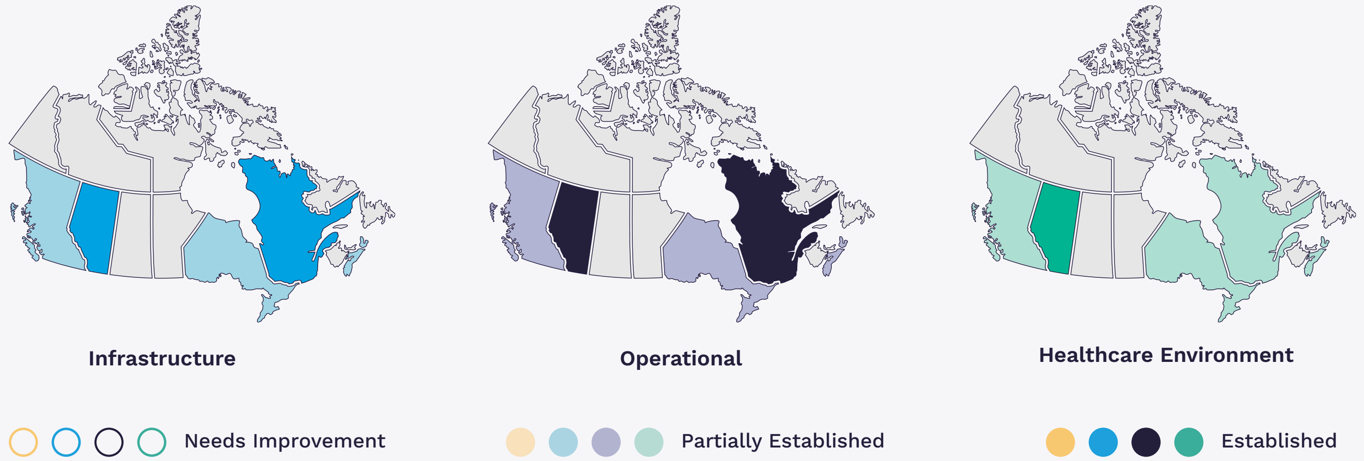

Overall, Canada is partially ready for a future of genomic medicine but making progress. The Canadian provinces in a better state of readiness for genomic medicine are Alberta, Quebec and British Columbia. This is largely in part to the earlier establishment of single, laboratory service organizations and programs that provide the necessary infrastructure for coordination and planning as well as necessary operational conditions.

In Nova Scotia, a higher level of coordination and planning is achieved due to lower levels of service demand and the ability of the government to work directly with the individual teaching hospitals which provide province-wide testing. However, many of the operational and evaluative processes are informal, and not public facing. The opposite is true in Ontario, which is challenged with much higher levels of demand for service, a complex web of formal evaluative processes, and until recently, a highly decentralized health system. Ontario has established a program dedicated to genetic testing in 2021, much later than Alberta and Quebec.

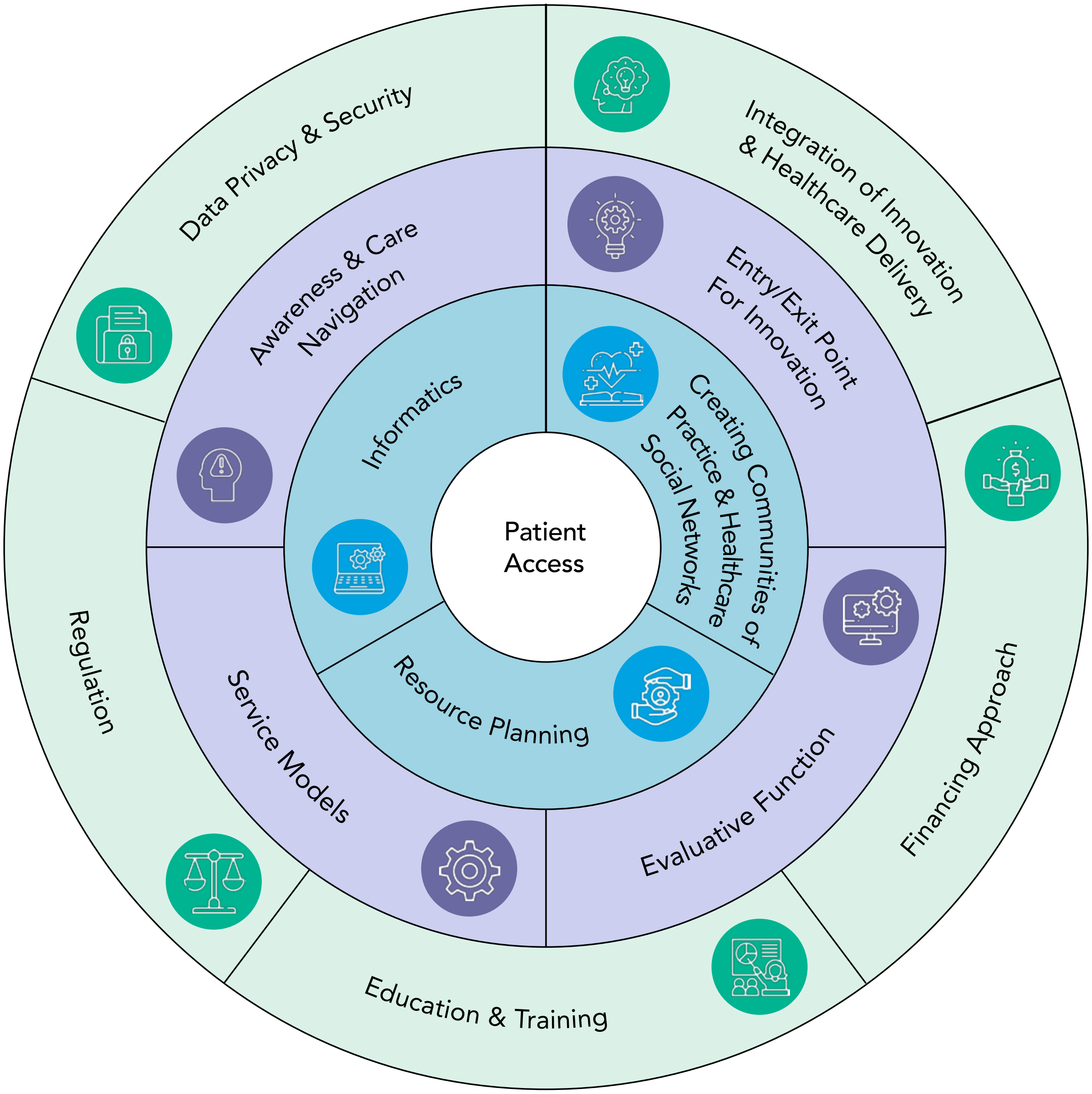

A further breakdown of conditions appears in the next section.

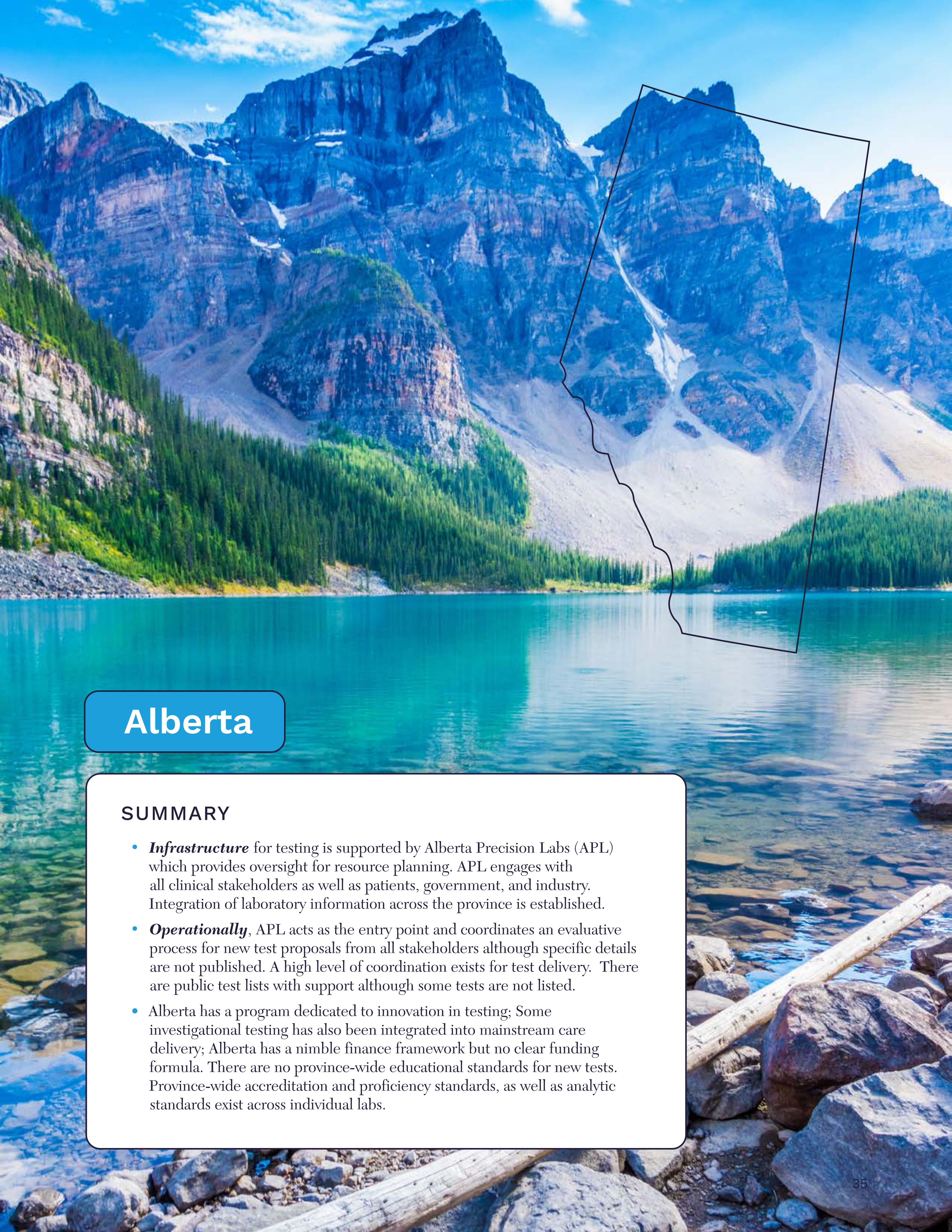

Alberta

SUMMARY

- ***Infrastructure*** for testing is supported by Alberta Precision Labs (APL) which provides oversight for resource planning. APL engages with all clinical stakeholders as well as patients, government, and industry. Integration of laboratory information across the province is established.
- ***Operationally***, APL acts as the entry point and coordinates an evaluative process for new test proposals from all stakeholders although specific details are not published. A high level of coordination exists for test delivery. There are public test lists with support although some tests are not listed.
- Alberta has a program dedicated to innovation in testing; Some investigational testing has also been integrated into mainstream care delivery; Alberta has a nimble finance framework but no clear funding formula. There are no province-wide educational standards for new tests. Province-wide accreditation and proficiency standards, as well as analytic standards exist across individual labs.

Alberta is Canada's fourth largest province by size and by population (approx. 4.2 million). *Responsibility for testing is provided by a single organization, Alberta Precision Laboratories (APL), which is a wholly-owned subsidiary of Alberta's single health authority, Alberta Health Services (AHS). Highly specialized genomic/genetic testing is delivered as a provincial program within APL directly reporting to executive medical and operational leadership. APL develops a provincial test menu and delegates testing to hospitals within Alberta's largest centres (Edmonton Zone and Calgary Zone) depending on program of care, including the University of Alberta, Alberta Children's Hospital, Stollery Children's Hospital, and University of Calgary, Foothills Medical Centre. Testing is also referred by APL to out-of-province providers for rarer conditions.*

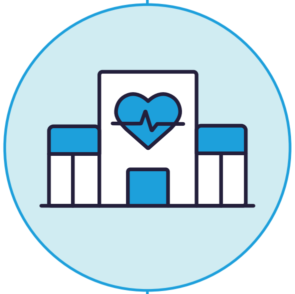

Infrastructure

APL provides an oversight function for the implementation of new tests on behalf of the AHS, through established networks across care providers. Resource planning is conducted/coordinated by APL. Alberta has worked toward substantial integration of its electronic medical record systems across the province, creating a single integrated laboratory information system for testing.

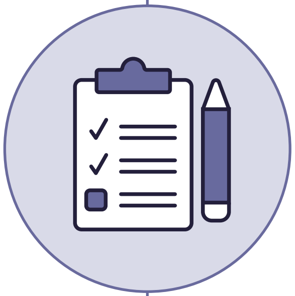

Operations

APL hosts a single-entry point for new testing. An intake form can be filled out by anyone (physicians, patients, innovators or the public). The review process results in recommendations and advice to AHS regarding funding. The test review process, timelines and criteria used are not publicly available. The review process may also look at decommissioning of tests. A new review process is under development. APL works with the AHS to provide provinical coordination (e.g., for referral and sampling) for testing. A test directory including navigational and supportive information is publicly available although it does not provide information on all available testing (e.g., such as rare, genetic testing).

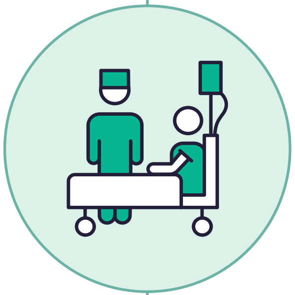

Environment

Alberta hosts a unique translational research program, called the Health Innovation Platform Partnerships, aimed at small to medium sized enterprises. This has, in turn, led to the development of the Alberta Diagnostic Ecosystem Platform for Translation (ADEPT) hosted at the University of Alberta, to allow innovators access to clinical samples and related data to test, validate and scale their technologies. Alberta does also fund both well-established and investigational genetic tests for some conditions. Funds can be released by AHS/APL for tests once an adoption decision is made, although tests with a large budgetary impact will require further consideration by the AHS. The funding formula for tests is not publicly available, but is made after examining the costs associated with testing. Analytic standards are developed by discipline councils that work with individual hospitals and laboratories who are given responsibility for testing through APL. Alberta uses its own province-wide accreditation and proficiency standards based on ISO 15189 standards.

Alberta's Strengths

- ***Infrastructure for coordination and planning*** - Alberta's single service organization, Alberta Precision Laboratories, provides oversight and resource planning and is able to leverage its position within Alberta Health Services to provide intraregional care coordination. It additionally acts as a single point of entry for proposals for new innovation, including proposals from innovators outside the health system.
- ***Advanced informatics*** – Integration of laboratory information systems across the province is established. There are also projects underway to fully integrate this with other electronic health data to give care providers a comprehensive picture of each patient.
- ***Innovative testing*** –Alberta hosts dedicated translational research programs for public and private sector innovators and incorporates investigational testing into mainstream healthcare. This practice makes Alberta prepared for the rapid future pace of the introduction of new tests, coupled with the potential research benefits associated with testing.

Alberta's Weaknesses

- ***Creating opportunities for innovation*** – the process and timelines for managing and deciding test priorities is not publicly available . There are also limited opportunities for exchange with innovators from outside of the healthcare system. This makes it difficult for innovators to plan their own development portfolios, understand the value of innovation to the Alberta health care system, or provide useful information that might benefit healthcare planning and priority setting
- ***Finance approach*** - Unlike traditional tests, funding formulas for genetic testing must consider the need for additional human resources associated with development and proficiency testing. It is not clear whether these resource requirements are adequately considered. Alberta's reliance on research funding to develop new tests means testing health system priorities are dictated by who is paying, rather than societal need, equity, or efficiency .
- ***Education and training*** – While there is education and training of care providers in the use of genetic testing, Alberta may benefit from province wide standards, to ensure consistency in the quality of testing service.

Takeaway: ***Alberta has many of the necessary conditions and is currently leading Canada in its state of readiness for genomic medicine. It requires a more transparent approach to create opportunities for innovation.***

Its current state of readiness has earned Alberta a grade of B+

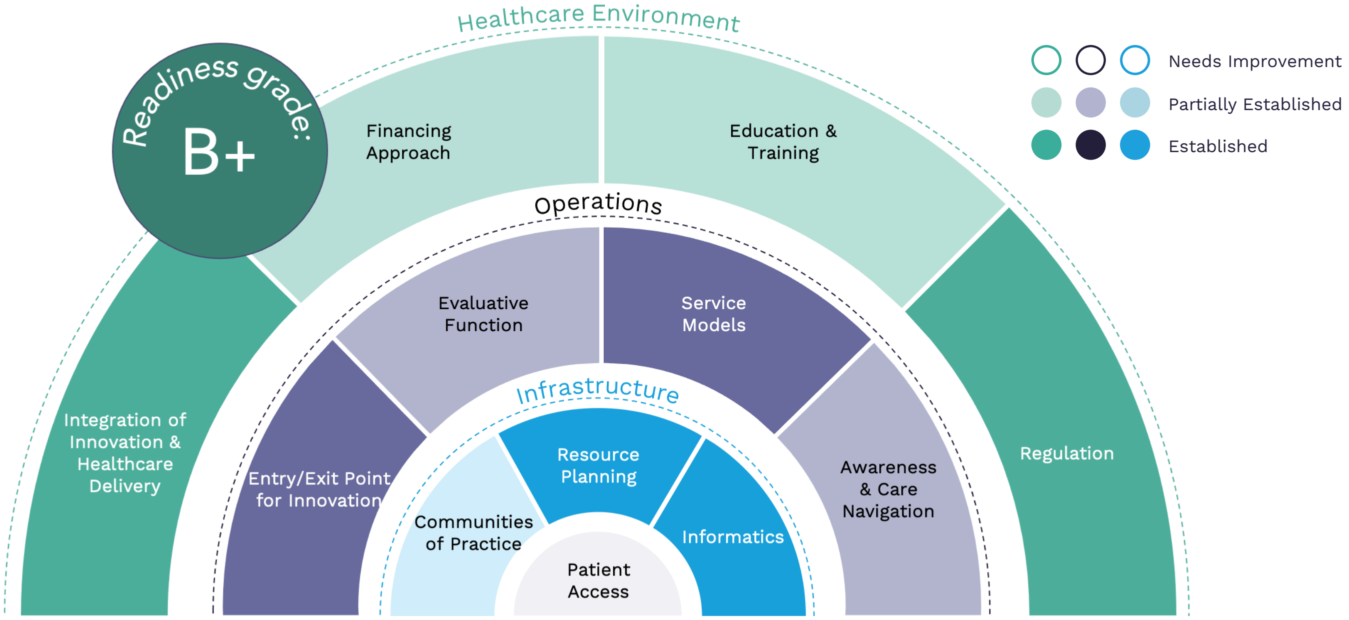

	Topic	Established	Partially Established	Need for Improvement
Infrastructure	**Creating communities of practice and healthcare system networks**	• The APL/AHS is responsible for intraregional care coordination		• Processes for engagement with external stakeholders lacking
Infrastructure	**Personnel, equipment, and resource planning**	• Systematic oversight for resource planning through the APL		
Infrastructure	**Informatics**	• Integrated LIS	• Projects underway to integrate laboratory and clinical data	
Operations	**Entry/exit point for innovation**	• Single point of entry through APL Test Review Process<br>• Open application process<br>• Some reassessment		• No explicit timelines for consideration
Operations	**Evaluative function**	• Clinical stakeholder engagement through Laboratory Test Formulary committee and Strategic Clinical Networks		• External stakeholder engagement lacking<br>• Evaluative criteria and process not made public
Operations	**Service models**	• Service coordination across providers		
Operations	**Awareness and care navigation**		• Test directory and ongoing communication to providers but not all tests (e.g., oncology) listed	
Environment	**Integration of innovation and healthcare delivery**	• Investigational testing funded as part of larger multigene panels<br>• Translational research through Genome AB, and the HIPP/ADEPT program		
Environment	**Financing approach**	• APL has flexibility to release additional funds for testing on a per-case basis		• Funding formula not clear<br>• No funding for test development
Environment	**Education and training**		• Training occurs but no province-wide standards for education and training	
Environment	**Regulation**	• ISO 15189-based province-wide accreditation standards<br>• Councils for creating analytic standards		

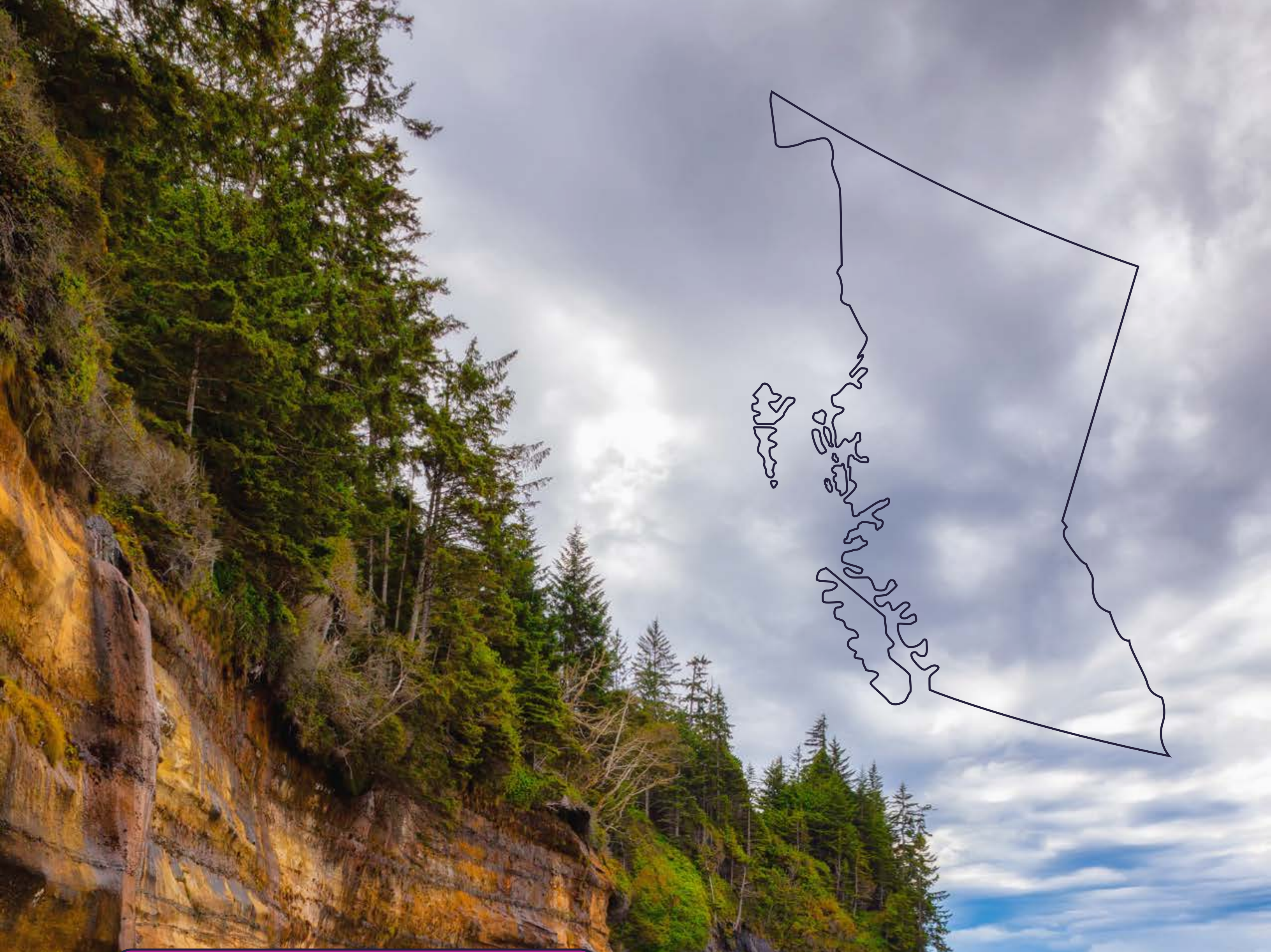

British Columbia

SUMMARY

- ***Infrastructure*** for testing is supported by BC Provincial Laboratory Medicine Services (PLMS) which provides oversight for resource planning. Integration of laboratory information is lacking but currently under development.
- ***Operationally,*** BC has an entry point for new test proposals with a consistent evaluative framework although process details and stakeholder engagement are lacking. A high level of coordination exists for test delivery although some intra-regional coordination is limited. There are no public test lists and limited navigational support.
- Some investigational testing has been integrated into mainstream care delivery; BC lacks a nimble or flexible finance framework or province-wide educational standards for new tests. Province-wide accreditation and proficiency standards exist and province-wide analytic standards are developed by individual labs.

Canada's second largest province by size and third largest by population (approx. 5 million) *has leveraged its single health authority dedicated to highly specialized services (the Provincial Health Services Authority, PHSA) to coordinate the delivery of genetic testing. Highly specialized testing is delegated to larger teaching hospitals (Vancouver General Hospital; St. Paul's Hospital; Royal Columbian Hospital; BC Children's Hospital) depending on type of test or therapeutic program. Testing is also referred to out-of-province providers for rarer conditions. The BC Provincial Laboratory Medicine Services (PLMS, formerly the BC Agency for Pathology and Laboratory Medicine, BCAPLM) is the Provincial Program under the PHSA which is responsible for the administration and provision of insured laboratory benefits to British Columbians.*

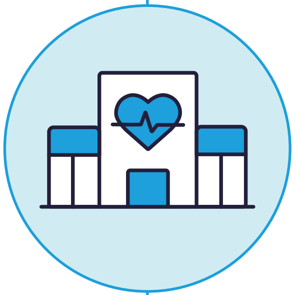

Infrastructure

The PHSA/PLMS provides an oversight function for the implementation of new tests on behalf of the BC Ministry of Health. The PLMS has established networks on its own and through the PHSA needed to understand testing priorities and logistics of implementation. Resource planning is conducted/coordinated by the PLMS along with the PHSA. A unique challenge in BC is that individual regions and hospitals have separate governance structures and do not have a single, integrated laboratory information system.

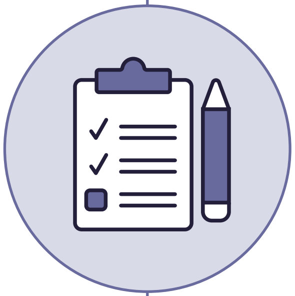

Operations

A single-entry point for new testing is provided through the PLMS test review process. The process is open only to employees and/or authorized agent of a laboratory provider, a health authority, the BC's Agency for Pathology and Laboratory Medicine or Ministry of Health and can have support from a co-applying physician. The review process results in recommendations / advice to the Ministry of Health regarding funding. While criteria for the test review process have been made available, the review process and rationale for test recommendations are not. Service coordination for testing is provided by the PLMS although regional coordination (e.g., for referral and sampling) is carried out by individual health authorities. There is currently no published test lists or protocols for care providers and navigation for access to testing is limited to private communication to specialist providers/centres and the use of health care navigators for some care programs.

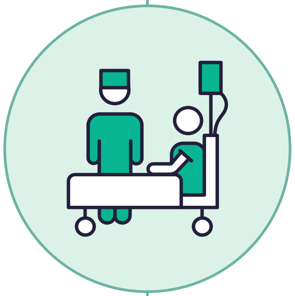

Environment

BC has a strong translational research environment with BC Michael Smith Genome Science Centre (GSC) receiving funding for sequencers and acting as a research arm of PHSA through its accredited laboratory. BC does fund both well-established and investigational genetic tests for some conditions. Funds can be released for tests once an adoption decision is made; for companion diagnostics in cancer, funding is provided through the systemic chemotherapy budget. The funding formula for tests is based on traditional community-based testing and requires revision. Additional funding for more complex testing can be released at the Ministry's discretion. There are no province-wide standards for education and training related to testing. Accreditation and proficiency is governed through a province-wide accreditation standard similar to CAP (the Diagnostic Accreditation Program [DAP] ISO 15189) Analytic standards are developed by individual hospitals and laboratories who are given responsibility for testing through the PLMS.

British Columbia’s Strengths

- ***Infrastructure for coordination and planning*** – British Columbia’s single service organization, British Columbia (BC) Provincial Laboratory Medicine Services (PLMS), supports resource planning and has been able to create a community of practice to facilitate service coordination. The PLMS also acts as a single point of entry for proposals for new innovation.
- ***Advanced informatics*** – BC has incorporated some investigational testing into mainstream healthcare. They have also developed translational research programs through Genome BC.

British Columbia’s Weaknesses

- ***Informatics*** – The integration of laboratory information systems needed to fully support the coming era of genomic medicine is lacking in BC. While there are projects underway to create a federated data commons, its current information structure could create barriers to implementing advanced testing.
- ***Creating opportunities for innovation*** – while BC has created communities of practice (intra-regional networks) to inform health system priorities, it lacks opportunities for exchange with private sector innovators external to the healthcare system. Its single point-of-entry for proposing valuable innovation is similarly closed to the private sector. This makes it difficult for innovators to plan their own development portfolios, understand the value of innovation to the BC health care system, or provide useful information that might benefit healthcare planning and priority setting.
- ***Navigation and tools to support patient care*** – Although the BC Ministry has adopted health service navigators which can support patient and care provider access to genome-based testing, it lacks test directories or protocols which may further support timely and effective care. There are similarly no province-wide standards for education and training of care providers that exist or are being developed.
- ***Finance approach*** – Although the BC Ministry has flexibility to release funds for testing on a case-by-case basis, it has used a volume-based formula for financing tests historically designed for community-based testing. Unlike traditional tests, funding formulas for genetic testing must consider the need for additional human resources associated with development and proficiency testing. BCs reliance on research funding to develop new tests means testing health system priorities are dictated by who is paying, rather than societal need, equity, or efficiency .

Takeaway: ***British Columbia is taking the necessary steps to advance its system readiness for genomic medicine. Some challenges relate to its decentralized health system structure (informatics, navigation, province-wide standards) while others may be more easily remedied.***

Its current state of readiness has earned British Columbia a grade of C

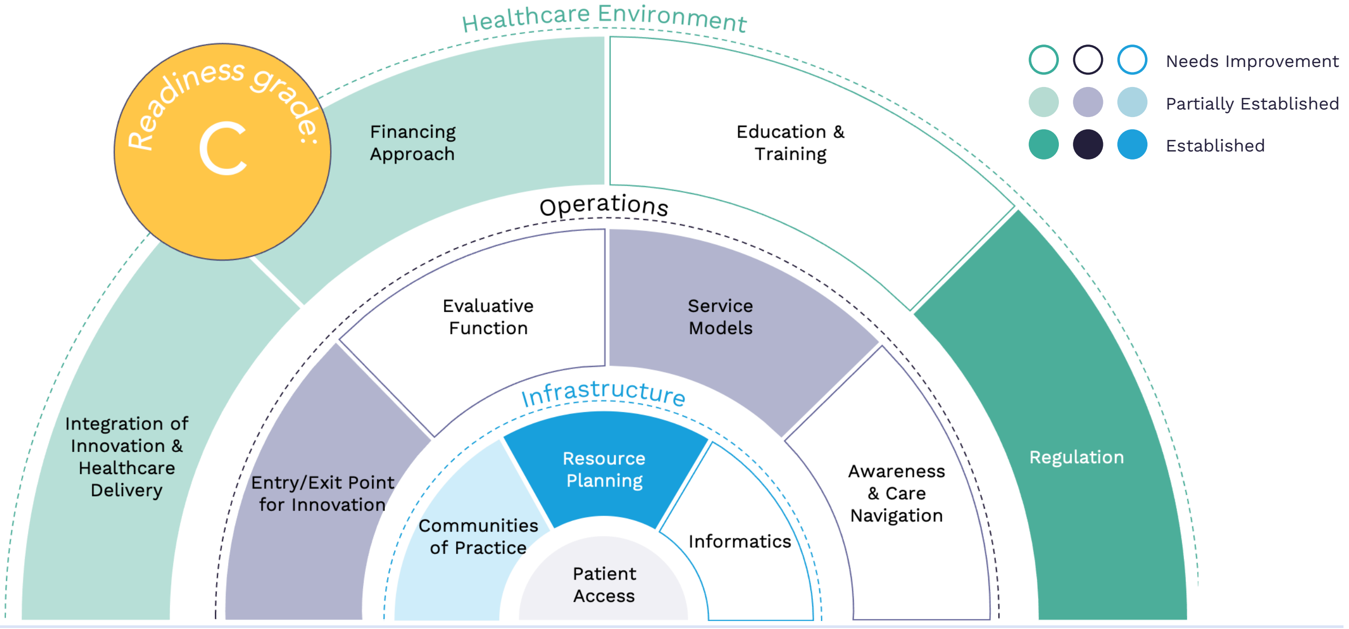

	Topic	Established	Partially Established	Need for Improvement
**Infrastructure**	**Creating communities of practice and healthcare system networks**	• The PHSA/PLMS is responsible for intraregional networks		• Engagement with industry stakeholders is lacking
	**Personnel, equipment, and resource planning**	• Systematic oversight for resource planning through the PLMS and strategic plan		
	**Informatics**		• Projects underway to create federated data commons	• Lack of integration of laboratory information across centres
**Operations**	**Entry/exit point for innovation**	• Single point of entry through PLMS Test Review Process<br>• Explicit timelines for consideration	• A genetics and genomics discipline committee also influences test introduction	• Closed application process<br>• No reassessment processes
	**Evaluative Function**		• Criteria published although scoring algorithm and rationale for recommendations not available	• No broad stakeholder engagement
	**Service models**	• Service coordination across providers through the PLMS	• Further coordination in health authorities	
	**Awareness and care navigation**		• Navigation for care providers and patients lacking, although BC does have nurse navigators	• No test directory or protocol but ongoing communication to providers
**Environment**	**Integration of innovation and healthcare delivery**	• Some investigational testing funded as part of larger multigene panels	• Translational research through Genome BC, and GSC	
	**Financing approach**		• Ministry has flexibility to release additional funds for testing on a per-case basis	• Funding formula designed for community-based testing<br>• No funding for test development
	**Education and Training**			• No province-wide standards for education and training in development
	**Regulation**	• DAP ISO 15189 province-wide accreditation standards<br>• Standards for analytic parameters or test proficiency are developed by individual centres with the PLMS	• No province-wide analytic standards although care often delivered through a single lab	

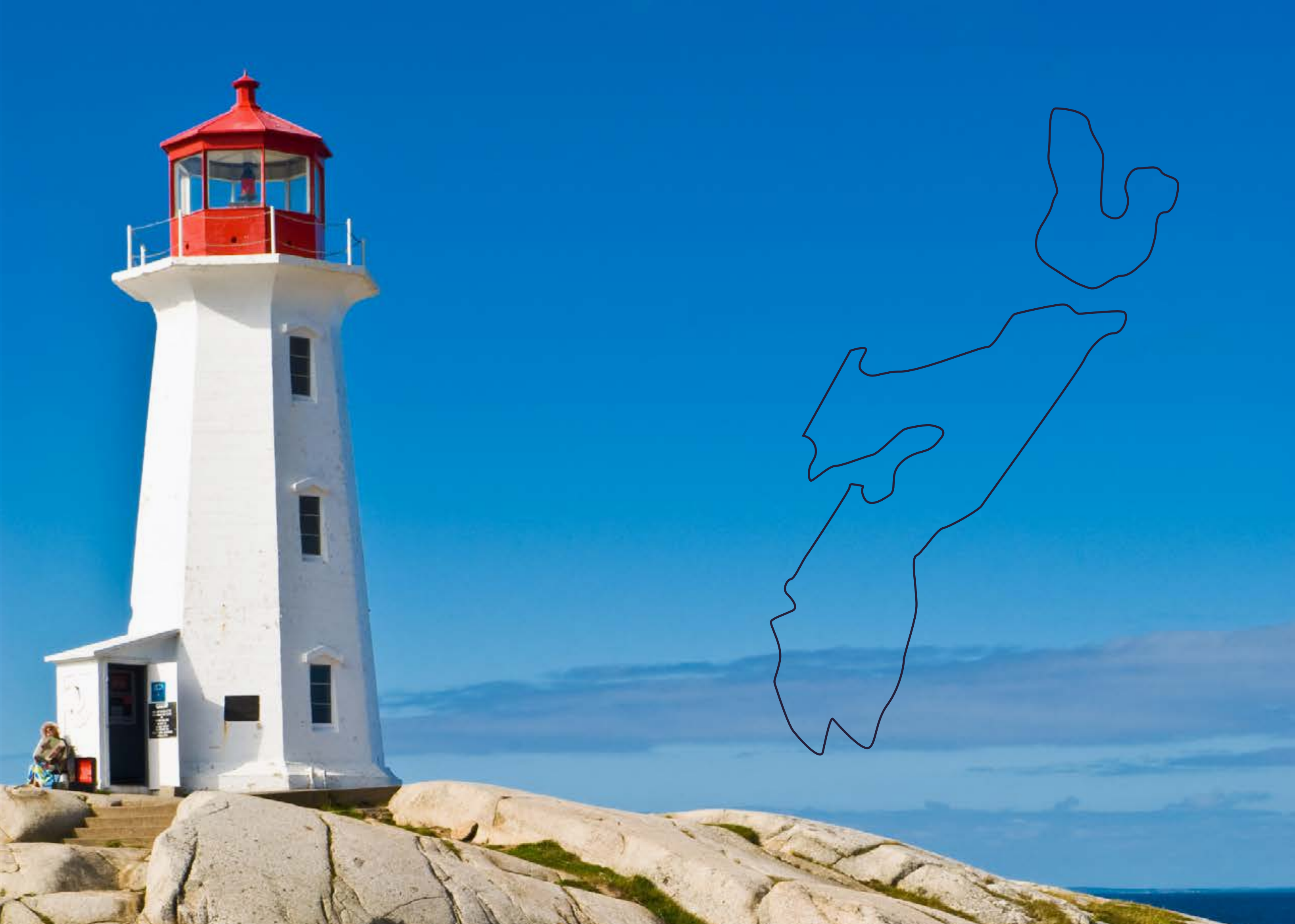

Nova Scotia

SUMMARY

- ***Infrastructure*** for testing is supported by the Nova Scotia health Authority (NSHA) which provides oversight for resource planning although does not broadly engage stakeholders. Integration of laboratory information is lacking.
- ***Operationally***, NS does not have an entry point for new test proposals or a consistent evaluative framework. A high level of coordination exists for test delivery. While NS has a test directory it is not comprehensive. Nova Scotia does communicate about available tests regularly with care providers.
- Investigational testing has been integrated into mainstream care delivery; NS has a flexible finance framework but the funding formula for testing is not clear. There are no or province-wide educational standards. There are similarly no analytic standards although tests are typically delivered by a single laboratory.

While Nova Scotia has a population of less than 1 million (less than 3% of Canada's population), *it is the most populous province in Canada's Atlantic region. Testing occurs within two major hospitals (Queen Elizabeth II Health Sciences Centre and IWK Health Centre) that deliver specialized care programs. Nova Scotia also uses out-of-province providers. Oversight for these programs is provided by the Nova Scotia Health Authority (NSHA) through its Pathology and Laboratory Medicine Program (PLMP).*

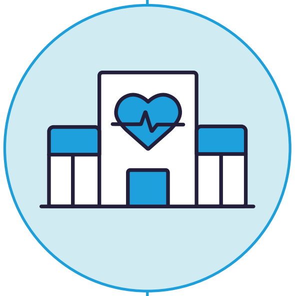

Infrastructure

The small population size of Nova Scotia has likely negated the need for large-scale healthcare networks. Communities of practice are established within specialized care programs as well as the Pathology and Laboratory Medicine Program. The oversight function for the implementation of new tests is the responsibility of the individual hospitals, who are in turn given a budget to deliver specialized programs of care. Analytic standards are developed by these individual hospitals as well as resource planning. A single, integrated laboratory information system for testing does not exist.

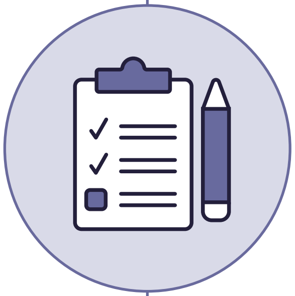

Operations

There is no single-entry point for new testing. Test review is done through a provincial advisory committee but the process, timelines and criteria used are not publicly available. A Laboratory Test Catalogue including navigational and supportive information is publicly available.

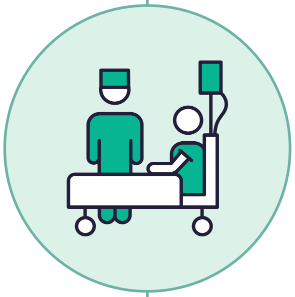

Environment

Nova Scotia provides access to both well-established and investigational genetic tests for some conditions. Funding mechanisms are hindered by reliance on hospital budgets and annual budget cycles. The funding formula for tests is not clear. There are no province-wide standards for education and training related to testing. Nova Scotia uses Canadian Counsel on Health Service Accreditation (CCHSA) province-wide accreditation standards based on ISO 15189 standards. Proficiency testing is voluntary .

Nova Scotia's Strengths

- ***Infrastructure for coordination and planning*** – Oversight, resource planning, and service coordination for testing is facilitated by Nova Scotia's single health care system, the Nova Scotia Health Authority through its through its Pathology and Laboratory Medicine Program (PLMP). Its smaller size relative to other provinces has allowed it to have a nimbler approach to the adoption and coordination for new testing.
- ***Innovative Testing*** – Nova Scotia has incorporated investigational testing into mainstream healthcare and was one of the first to do so in Canada. This in turn led to the earlier adoption of necessary tests that qualify patients for targeted cancer therapies.

Nova Scotia's Weaknesses

- ***Creating opportunities for innovation*** – Nova Scotia still lacks a single point of entry for onboarding new tests. Proposals for new testing are restricted to NHSA personnel through an undefined process of priority setting. Coupled with limited opportunities for engagement with innovators outside of the health system, these conditions make Nova Scotia less receptive to innovation.
- ***Informatics*** – Nova Scotia lacks geographic integration of laboratory information across its key service delivery centres.
- ***Navigation and tools to support patient care*** – Although Nova Scotia has developed some tools to support patient and care provider navigation, there are no province-wide standards for education and training of care providers that exist or are being developed.

Takeaway: ***Nova Scotia state of readiness for genomic medicine is aided by its size and established teaching hospitals. However, many of its processes are unclear. Nova Scotia would benefit from further integration and engagement with the broader innovation community.***

Its current state of readiness has earned Nova Scotia a grade of C-

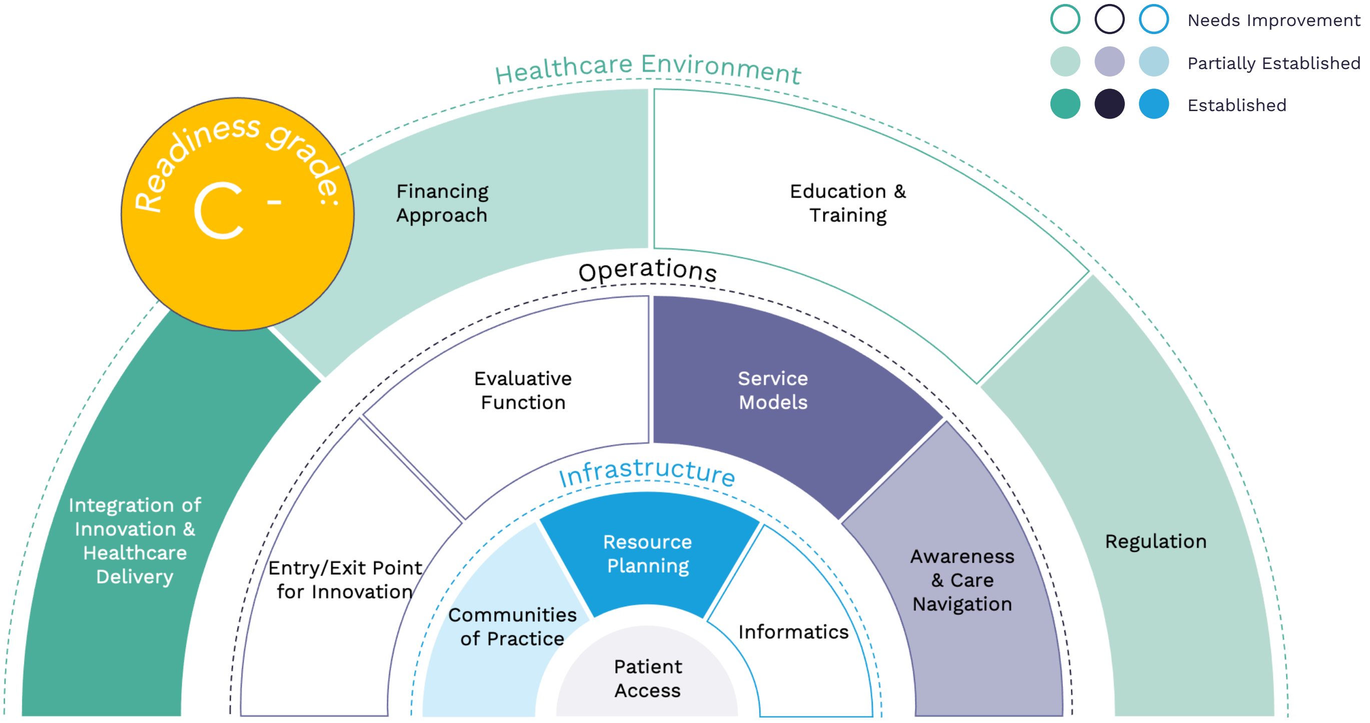

	Topic	Established	Partially Established	Need for Improvement
Infrastructure	**Creating communities of practice and healthcare system networks**	• Communities of practice exist within specialized programs and PLMP.		• Processes for broad stakeholder engagement lacking
	**Personnel, equipment, and resource planning**	• Systematic oversight for resource planning through the PLMP		
	**Informatics**			• Lack of integration of laboratory information across centres
Operations	**Entry/exit point for innovation**			• No single entry point<br>• Proposals from NSHA personnel<br>• No explicit timelines or reassessment process
	**Evaluative Function**			• No broad stakeholder engagement<br>• Evaluative criteria and process not made public
	**Service models**	• Service coordination across providers		
	**Awareness and care navigation**		• Test directory and ongoing communication to providers but not all tests (e.g., oncology) listed	
Environment	**Integration of innovation and healthcare delivery**	• Investigational testing funded as part of larger multigene panels		
	**Financing approach**	• NSHA has flexibility to release funds for testing on a per-case basis		• Funding formula not clear
	**Education and Training**			• No province-wide standards for education and training in development
	**Regulation**	• ISO 15189-based province-wide laboratory accreditation standards	• No province-wide analytic standards although tests often delivered through a single lab<br>• Proficiency testing voluntary	

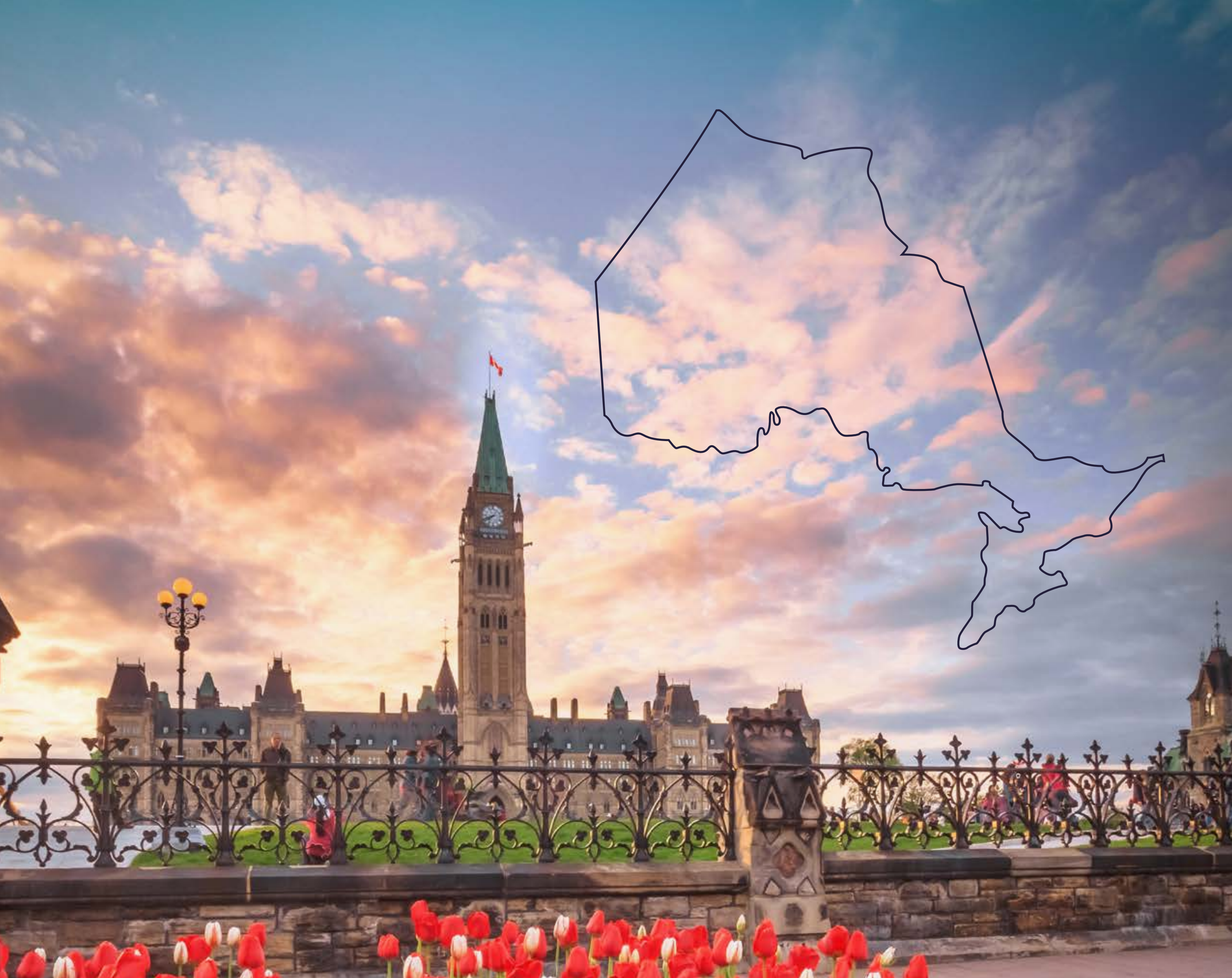

Ontario

SUMMARY

- Ontario's ***infrastructure*** for testing is still in early development. A new Ontario Health Provincial Genetics Program (PGP) was created in 2021 which will develop necessary conditions related to operations and infrastructure.
- Ontario's does not have a clear and consistent entry point or evaluative framework for considering new tests. It also does not engage innovation stakeholders outside of its healthcare environment. Some level of coordination and navigational support exists.
- Ontario has some integration of investigational and established testing; it lacks a nimble finance framework or province-wide analytic and education standards for new tests. Province-wide accreditation standards exist.

***Ontario is the largest of Canada's 13 provinces and territories by population (approx. 14.8 million**[1], with the vast majority of the province's inhabitants located in its southernmost regions) and third-largest by size. Capacity for genetic testing largely resides in its hospitals with testing for hereditary disease largely occurring in Ontario's two children's hospitals. Some testing is commissioned to out-of-province providers as well. Somatic testing is conducted across 11 centres of varying sizes.*

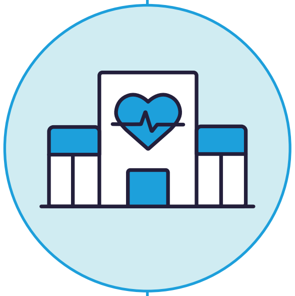

Infrastructure

Networks of genetic testing providers were originally established through the Regional Genetics Program, the Ontario Molecular Pathology Research Network, Tumour Site groups and other Clinical Programs, such as the Pathology & Laboratory Medicine Program (PLMP). Overarching coordination has now become the responsibility of the newly (2021) established Ontario Health Provincial Genetics Program (PGP) which may utilize these networks or create new ones. Resource planning for genetics is still conducted on a hospital- or regional level through regional bodies of Ontario Health as Ontario moves toward a more centralized model of care delivery. Working Groups were established in 2017 to examine the health human resources required for clinical genetic services, and this, along with data and digital systems is now a focus of the newly established Provincial Genetics Program.

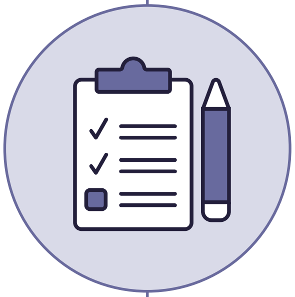

Operations

There is no single entry point for new testing. The current Ontario Health (Quality) process allows manufacturers of commercial innovation and translational researchers to apply for assessment, however priorities for what ultimately gets assessed is assigned to OHQ. Beyond this, proposals for new testing rely on practicing clinicians and other internal stakeholders (such as Pharmacy Services). Tests may be evaluated through several routes including the Program in Evidence-Based Care / Tumor Site Groups, Ontario (Quality) / Ontario Genetics Advisory Committee, Ontario Public Drug Programs / The Ontario Steering Committee for Cancer Drugs (OSCCD), and Newborn Screening Ontario / The Newborn Screening Ontario Advisory Council (NSO-AC) which use different evaluative frameworks. Coordination across institutions is done through the PGP for hereditary and somatic testing. There is a defined test list for both hereditary and somatic testing; navigation is largely provided by genetic testing centres, specialty clinics or patient organizations and the PGP is planning to consolidate these navigational resources for patients and providers.

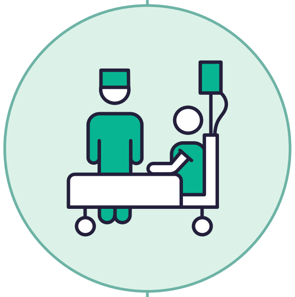

Environment

In May 2021, Ontario committed to implementing comprehensive cancer testing including genetic panels that include both established and investigational tests. There are also implementation pilot projects such as the Genome-wide Sequencing Ontario (GSO) aimed at providing genome-wide sequencing to 2000 Ontario families. Ontario still largely relies on annual budget cycles and Minstry decisions to release funding for new tests. Accreditation and proficiency is based on the ISO15189

Ontario's Strengths

- ***Infrastructure for coordination and planning*** – Ontario has recently created a single-service organization, the Provincial Genetics Program (PGP), which is intended to coordinate service delivery, support resource planning, and develop awareness and navigational tools.
- ***Quality assurance*** – Ontario has adopted clear standards for accreditation of laboratories and proficiency testing, although there are as yet no province-wide analytic validation standards.

Ontario's Weaknesses

- ***Finance approach*** **–** The current finance approach for new testing in Ontario lacks the ability to release funds at the pace of genomic innovation. This has led to delays which have impacted patient care. In turn, Ontario has relied on private and public research funding to develop new tests. This means testing priorities are dictated by who is paying, rather than societal need, equity, or efficiency . Similarly, Ontario's funding formula for new tests does not consider the need for additional human resources associated with development and proficiency testing, which may make it unsustainable.
- ***Informatics*** **–** Ontario is still a highly decentralized healthcare environment, and this has led to a lack of integration of laboratory information systems needed to fully support the coming era of genomic medicine. Integration of laboratory information along with health information has become a recent area of focus for development.
- ***Creating opportunities for innovation*** **–** Ontario relies on a number of existing and overlapping communities of practice but has limited exchange with private and public sector innovators external to the healthcare system. There is no single point-of-entry or single evaluative framework for proposing valuable innovation. Only one entry point is open to commercial innovators (OHQ) and it has a limited capacity to evaluate proposals. This makes it difficult for innovators to plan their own development portfolios, understand the value of innovation to the Ontario health care system, or provide useful information that might benefit healthcare planning and priority setting. Similarly, Ontario's program to attract investment in clinical trials is not linked to improving its testing function.
- ***Navigation and tools to support patient care-*** Ontario has recently published an updated test list for cancer and has plans to create a consolidated resource centre for care providers for all testing. There are currently no province-wide standards for education and training of care providers.

Takeaway: ***Ontario has only recently taken necessary steps to improve its state of readiness for genomic medicine. It currently lacks many of the necessary conditions to be prepared.***

Its current state of readiness has earned Ontario a grade of D

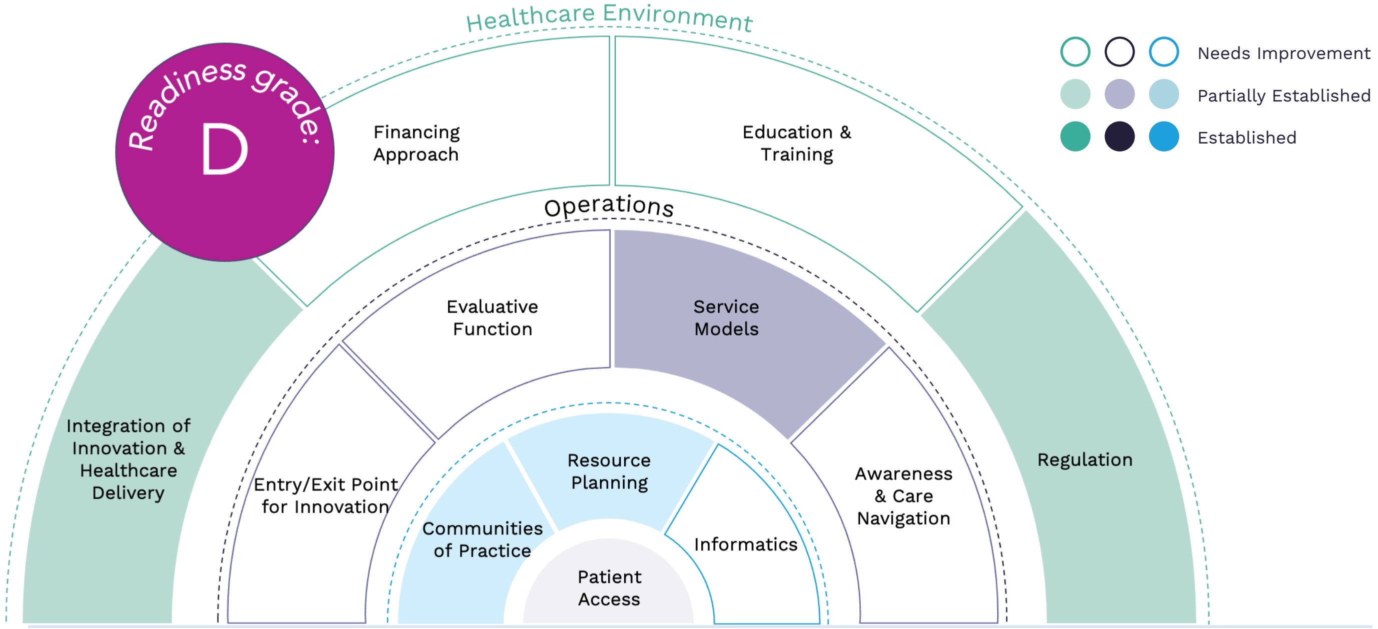

	Topic	Established	Partially Established	Need for Improvement
Infrastructure	**Creating communities of practice and healthcare system networks**		• Laboratories work independently coordination through PGP/PLMP<br>• Networks being developed through PLMP and PGP-led clinical leadership forum	• Processes for engagement with commercial innovators lacking
	**Personnel, equipment, and resource planning**		• Working group report published in 2018<br>• New PGP area of focus – completed for cancer genetics	
	**Informatics**		• Some integration of EHR with laboratory information<br>• Linking laboratory information systems new area of focus	• No across-province integration of laboratory information
Operations	**Entry/exit point for innovation**		• Open proposal process newborn and prenatal testing; OGAC reviews	• No single point of entry<br>• No explicit timelines<br>• No reassessment process
	**Evaluative Function**		• Some evaluation trans-parent (e.g., OGAC) with some stakeholder engagement<br>• Ongoing evaluation through PGP-led expert and advisory groups	• No consistent evaluative framework<br>• Multiple evaluation frameworks with some not timely
	**Service models**		• Partially established through PGP	
	**Awareness and care navigation**		• Test navigation resource in development<br>• Test lists available	
Environment	**Integration of innovation and healthcare delivery**		• Some testing regimes combine established and investigational testing<br>• Some large-scale implementation projects with industry partners	
	**Financing approach**			• Funds available on annual budget cycles or sometimes not available at time of adoption<br>• Funding formula not clear<br>• No funding for test development, additional human resource costs
	**Education and Training**			• No province-wide standards for education and training in development
	**Regulation**	• Accreditation and proficiency are based on the ISO15189		• No province-wide analytic validation standards

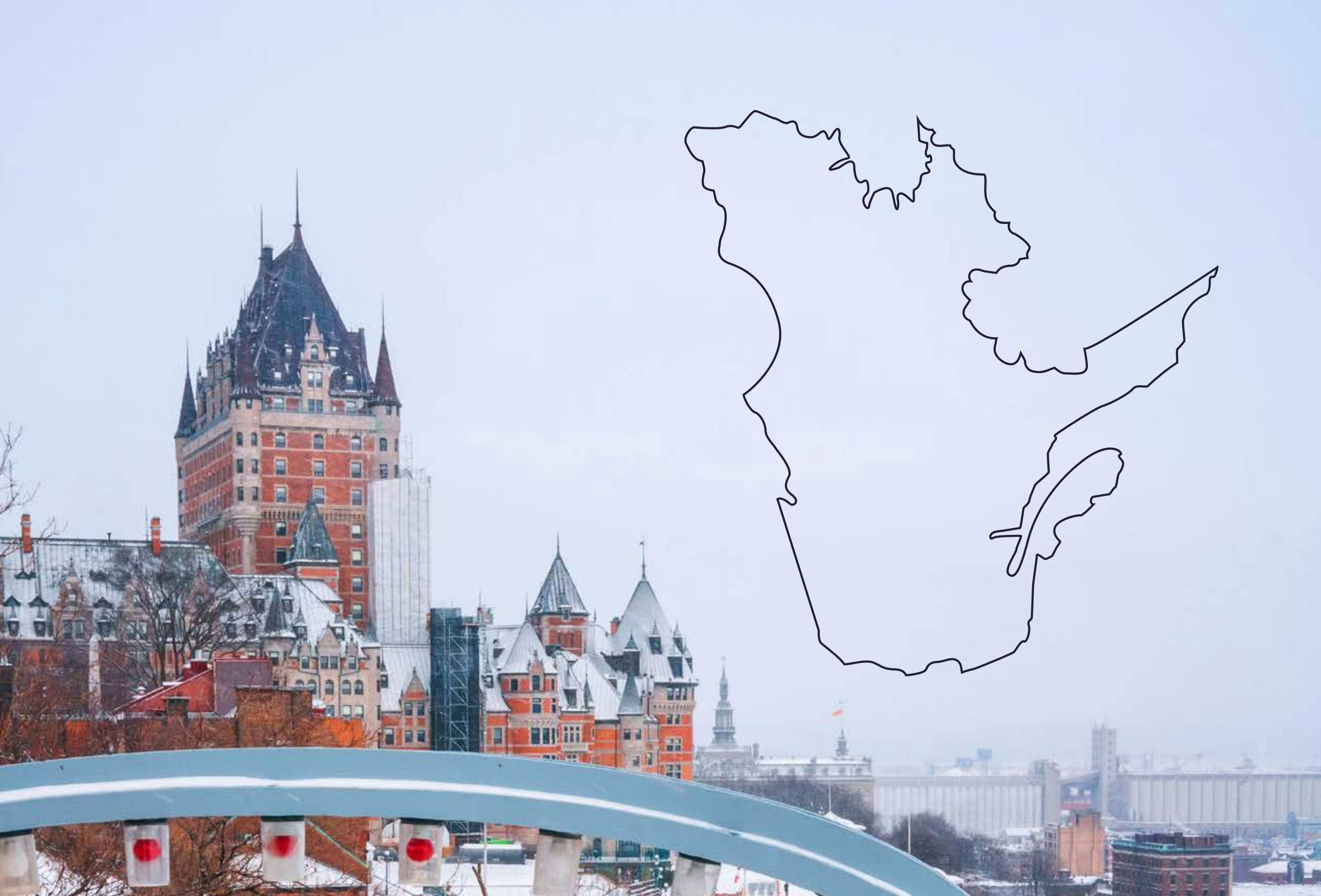

Quebec

SUMMARY

- Planning, development and funding falls under the Direction de la biovigilance et de la biologie médicale (DBBM) which is part of the Direction générale des affaires universitaires, médicales, infirmières et pharmaceutiques (DGAUMIP) within Quebec's Ministry of Health. The Réseau Québécois de Diagnostic Moléculaire (RQDM) acts as the supporting network for genetic testing.
- ***Operationally,*** Quebec offers a single point of entry for testing although this is closed to industry. It has a well-defined and consistent evaluation function for testing with limited stakeholder engagement. There is some service coordination for testing although more is in development. Navigational supports are lacking and a current development priority.
- Quebec has limited integration of investigational and established testing. It has a nimble financing approach and offers resources to support test validation. Province-wide standards for education are being developed. Province-wide accreditation and proficiency standards exist although there are province-wide analytic standards.

***Canada's largest province by size and second largest by population (approx. 8.5 million)** began reform on its system of laboratory governance in 2011. Molecular diagnostics including low- to medium-throughput sequencing is delivered across five "clusters" operating seven supra-regional laboratories (Capitale-Nationale [CHU de Québec – Université Laval]; Estrie [CHUS – Hôpital Fleurimont]; Montréal – CHUM [CHUM and Hôpital Maisonneuve-Rosemont]; Montréal – CUSM [CUSM and Hôpital général Juif]; Montréal – CHU Sainte-Justine [CHU Sainte-Justine]) as well as the Montreal Heart Institute (MHI). The Centre québécois de génomique clinique (CQGC) in 2018, physically situated at the Centre hospitalier universitaire Sainte-Justine (CHU Sainte-Justine), was established to conduct high-throughput (exome, transcriptome or whole-genome) sequencing. Testing is also referred to out-of-province providers for rarer conditions. The Direction de la Biovigilance et de la Biologie Médicale (DBBM) is the Ministry Program that has been tasked with coordinating the implementation of molecular diagnostic testing across all of these centres/clusters.*

Infrastructure

Under the DBBM, the Réseau Québécois de Diagnostic Moléculaire RQDM acts as the supra-regional network and advisory function for the DBBM on behalf of the Quebec Ministry of Health. Resource planning for genetics is conducted by the seven centres/clusters (five regions, MHI and CQGC) and is coordinated by the DBBM. Further coordination across oncology centres (through the Quebec cancer program [PQC]) is now underway, given a recognized lack of coordination in cancer. A laboratory information system is being established across this network.

Operations

The DBBM acts as an entry point for new testing. The DBBM works with Institut national d'excellence en santé et en services sociaux (INESSS) to provide advice to the Ministry of Health regarding funding. Only public laboratories can submit requests to DBBM for evaluation by INESSS. In the case of companion diagnostic tests, drug manufacturers must submit the diagnostic test evaluation request with the drug evaluation request. Tests are evaluated through a single evaluative framework and recommendations to the Ministry are made public, although there is limited engagement with stakeholders with this evaluation process. While there is a test formulary (the Répertoire québécois et système de mesure des procédures de biologie médicale), it may not always be clear how and where a test can be made available to patients. The RQDM is currently working on additional navigational support.

Environment

Quebec has continued with a policy of only funding medically necessary tests and will not pursue the reporting of investigational tests that are associated with unfunded targeted therapies. Translational research projects are conducted through the CQGC and Genome Quebec is asked to participate in the development and valida-tion of standard operating procedures for high-throughput testing. Funds can be released by the Ministry once an adoption decision is made, and funding for test development, and additional human resource costs has more recently (2021) been provided. It is unclear whether the funding formula is value-based or amenable to reassessment. The DBBM/RQDM has committed to developing province-wide standards for education and training. Accreditation and proficiency are regulated through a province-wide accreditatio standard (ISO 15189). There are no province-wide analytic standards.

Quebec's Strengths

- ***Infrastructure for coordination and planning*** – Systematic oversight and resource planning for testing in Quebec is provided through its Ministry-led Direction de la Biovigilance et de la Biologie Médicale (DBBM), which in turn has created a single supraregional network (Réseau Québécois de Diagnostic Moléculaire (RQDM)). It additionally acts as a single point of entry for proposals for new innovation from within the health system.
- ***Evaluative approach*** – The DBBM utilizes the already established health technology assessment function (INESSS), which has a transparent, well-defined and consistent process for evaluation.
- ***Finance approach*** – Quebec has a unique finance approach among Canadian provinces, and provides funding for test development and additional human resources associated with development and proficiency testing. Funds can be made available quickly at the time of adoption — conditions needed for readiness. Quebec's unique approach means it is able to more comprehensively manage its testing service.
- ***Advanced informatics*** – Integration of laboratory information systems across the province is established. There are also projects underway to fully integrate this with other electronic health data to give care providers a comprehensive picture of each patient.

Quebec's Weaknesses

- ***Tools to support timely and appropriate use*** – While Quebec does publish a test list ('repertoire'), it still lacks information and other supportive tools regarding available tests or access to tests. Province-wide standards for education and training in development.
- ***Creating opportunities for innovation*** – Quebec has limited its opportunities for innovation to a translational research program through the CQGC; it still does not engage private sector innovators more broadly or accept applications for proposals of innovation. Quebec has also limited the use or reporting of investigational tests (for which there are no immediate healthcare decisions to be made) in mainstream healthcare which reduces opportunities for wider clinical research and attracting clinical trials. In sum, these policies make it difficult for innovators to plan their own development portfolios, understand the value of innovation to the Quebec health care system, or provide useful information that might benefit healthcare planning and priority setting.

Takeaway: ***Quebec began taking necessary steps to reform its approach to genome-based testing over a decade ago. There are still opportunities to improve the optimal use of testing in Quebec.***

Its current state of readiness has earned Quebec a grade of B -

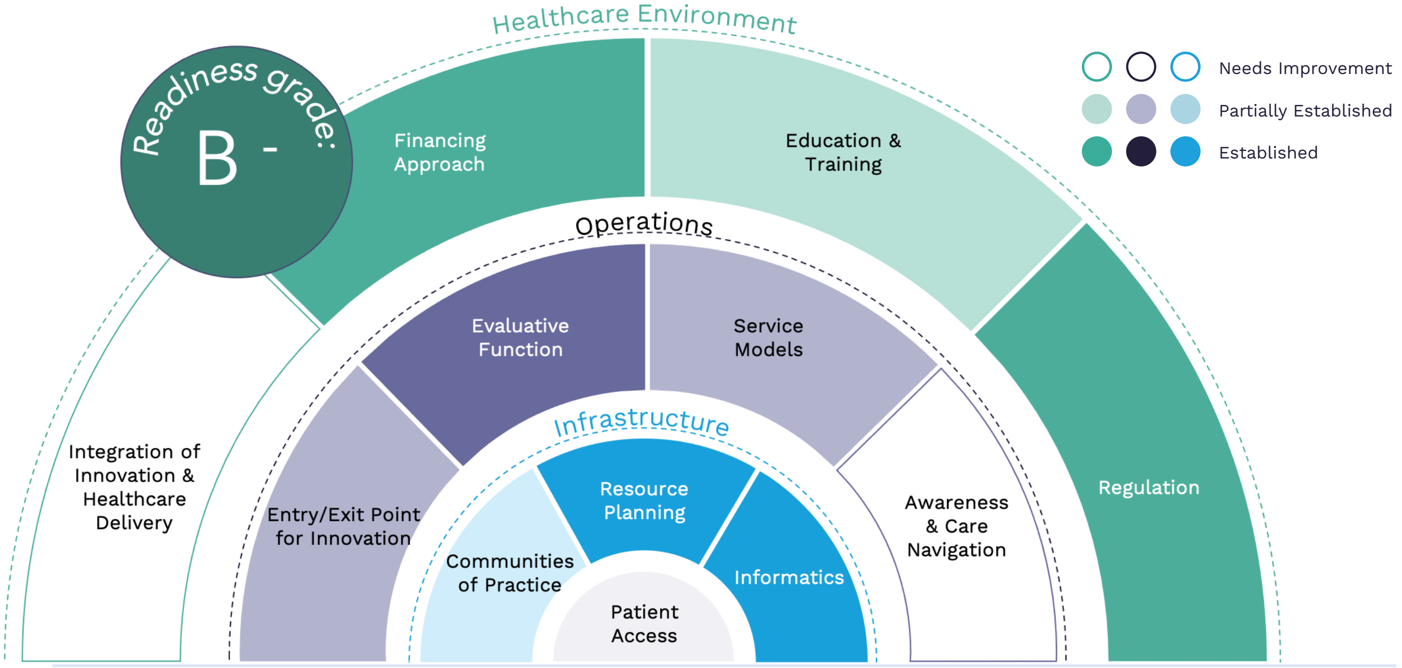

	Topic	Established	Partially Established	Need for Improvement
**Infrastructure**	**Creating communities of practice and healthcare system networks**	• The RQDM acts as the supra-regional network and coordinating function		• Processes for broader stakeholder engagement lacking
	**Personnel, equipment, and resource planning**	• Systematic oversight for resource planning through the DBBM		
	**Informatics**	• Integration of LIS across centres beginning fall, 2022	• Some integration of EHR with laboratory information	
**Operations**	**Entry/exit point for innovation**	• Single point of entry through DBBM<br>• Explicit timelines for consideration		• Closed application process<br>• No reassessment process
	**Evaluative Function**	• Well-defined and consistent process for INESSS	• Some stakeholder engagement	
	**Service models**	• Service coordination across providers	• Need for further coordination in oncology	
	**Awareness and care navigation**		• Test list (“repertoire”) available but lacks some information regarding available tests or access to tests	• Navigation for care providers and patients lacking
**Environment**	**Integration of innovation and healthcare delivery**		• Translational research through Genome Quebec and the CQGC	• Investigational testing not funded
	**Financing approach**	• Clear funding formula with additional funding for test development, additional human resource costs<br>• Funds available at time of adoption		
	**Education and Training**		• Province-wide standards for education and training in development	
	**Regulation**	• ISO 15189 province-wide accreditation and proficiency standards<br>• Standards for analytic parameters or test proficiency are develope by the CQGC		

Chapter 5.

Impact of System Readiness in Canada

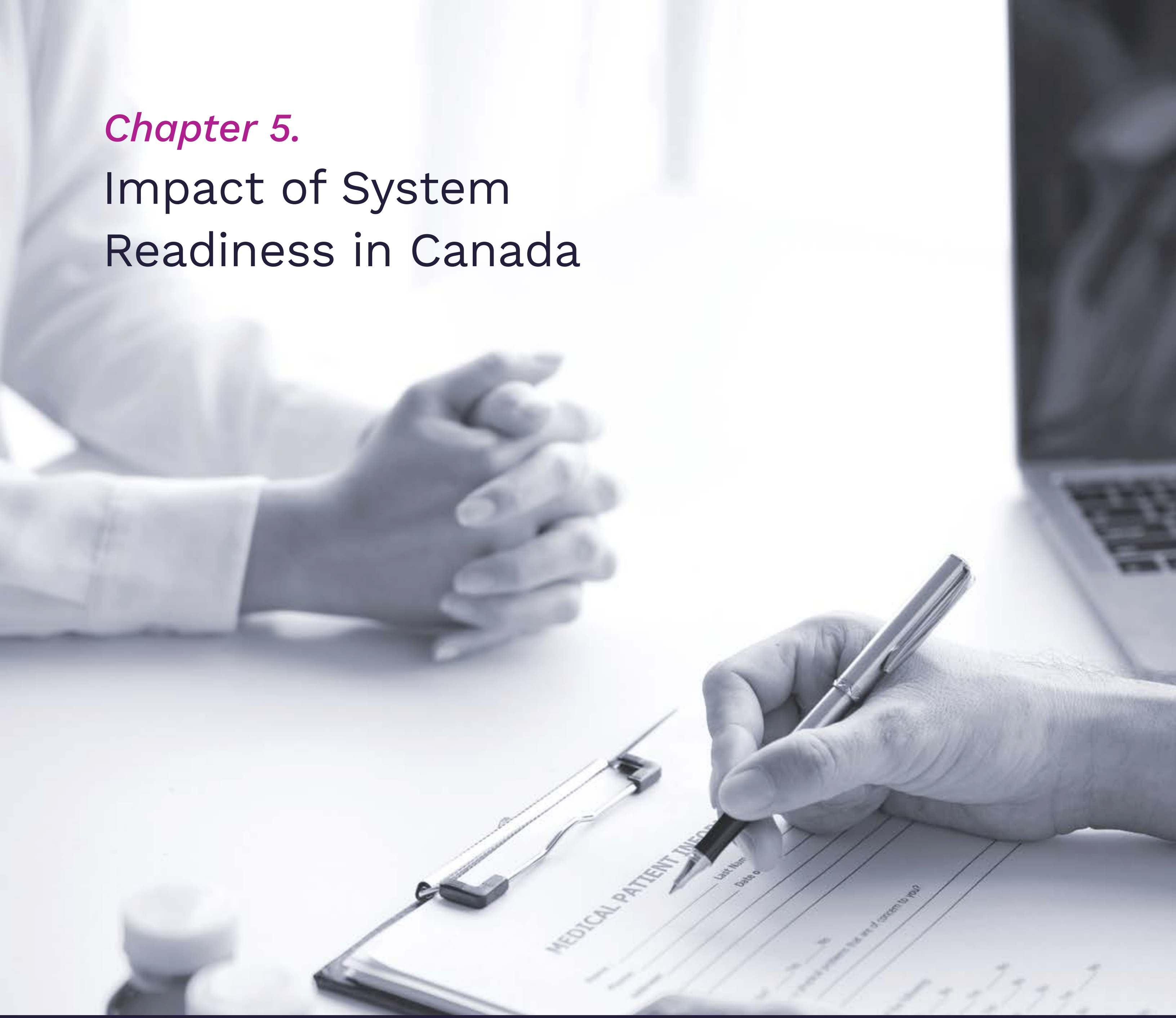

CHAPTER SUMMARY

- Healthcare system readiness for genome-based testing can be expected to yield positive impacts on important patient health outcomes, including alleviating symptoms, reducing harm from therapy, and improving survival and quality of life.
- Beyond these immediate benefits, readiness can also have a positive impact on patient and caregiver experiences, reducing the need for referrals and other diagnostic tests, and improving time to diagnosis and treatment..
- Unlike many conventional health technologies, testing can play a critical role in future scientific discovery and clinical trial enrollment, creating commercial and investment opportunities as well as future-proofing Canada's healthcare workforce.
- Taken together, readiness for genomic medicine represents an opportunity to reduce healthcare costs while creating the necessary infrastructure for delivering 21st century care.

Creating the necessary conditions for efficient and effective genomic medicine is a current and ongoing challenge for policymakers and healthcare administrators. A fundamental question is what impacts can be anticipated to the healthcare system and society, more broadly. Healthcare system impact can be thought of in terms of the "quadruple aim" of reducing per capita costs while improving population health outcomes, patient and caregiver experiences, and provider experiences. The broader impacts of innovation include providing an infrastructure for scientific discovery including clinical trials and the associated benefits of attracting and establishing a strong clinical workforce.

Healthcare Impact

Patient Health Outcomes

Improving the state of readiness for genomic medicine can have a wide range of positive impacts on population health. These impacts will ultimately depend on how and in whom the test is used. Genome-based tests evaluated for use in Canada and abroad over the last 7 years have described the following benefits on patient health which have been accepted by health technology assessment bodies: increasing diagnostic yield (number of cases detected)[1–5]; preventing future disease diagnosis in patients and genetic relatives[3]; reducing severe toxicities from drug therapy or the need for drug therapy[6–8]; preventing or delaying complications and symptoms from disease; improvement in health-related quality of life[9]; avoiding invasive procedures[8,10]; improving response to medication[11]; and increasing disease survivability[12].

While improving population health through genomic medicine is encouraging, there may also be harmful effects to patients or genetic relatives, particularly when genetic variants are discovered but without enough information to understand potential consequences (i.e., variants of unknown significance, or VUS).[13–15 Response to uncertainty from test results varies by therapeutic context and can be mitigated by counselling and other supports, including improved testing protocols, better VUS classification, and routine family-based assessment of variants. There may also be secondary or incidental findings (i.e., not related to the initial reason for testing) related to more comprehensive genomic analyses that can be mitigated through technical procedures and more-nuanced informed consent procedures. Canadian guidance has been developed to mitigate unintentional harm to patients and family members from these unintended effects.[16]

Patient and Healthcare Provider Experiences

Appropriate testing may create additional benefits for patients and their healthcare providers beyond health outcomes. When used for screening or diagnosis, this includes reducing the need for additional diagnostic tests (including invasive biopsy)[2,8,10]; improving time to diagnosis[4]; reducing unnecessary surgeries[17]; reducing the number of specialist referrals and follow-up visits[10,18]; reducing medication burden[11]; and improving equity of access to testing and utilization of testing[18].

Cost Impact

Although genomics-enabled precision medicine has been described as a "game changer" by some[19], accumulating evidence provides a more nuanced and compelling picture—the potential impact of genomic medicine on offsetting or reducing healthcare costs can vary widely.[20–22] Economic evaluations on the use of genetic services in healthcare, including prenatal care, screening and diagnosis, and the use of genome-based testing to guide therapeutic decision-making consistently show a wide range of impacts, from significant cost savings to expenditure that might be seen as unaffordable.

Fortunately for policymakers, this is not the same as the adoption of a new drug or new medical procedure with a wide possible range of impacts. Establishing a state-of-the-art genomic medicine service along with appropriate mechanisms for evaluation allows any jurisdiction to select those interventions or programs of care that can potentially deliver cost savings or benefits at reasonable/tolerable levels o additional expenditure. A purposive sample of evaluations relevant to Canada, in Table 2, illustrates the wide range of cost impacts from testing, and the potential policy response to this.

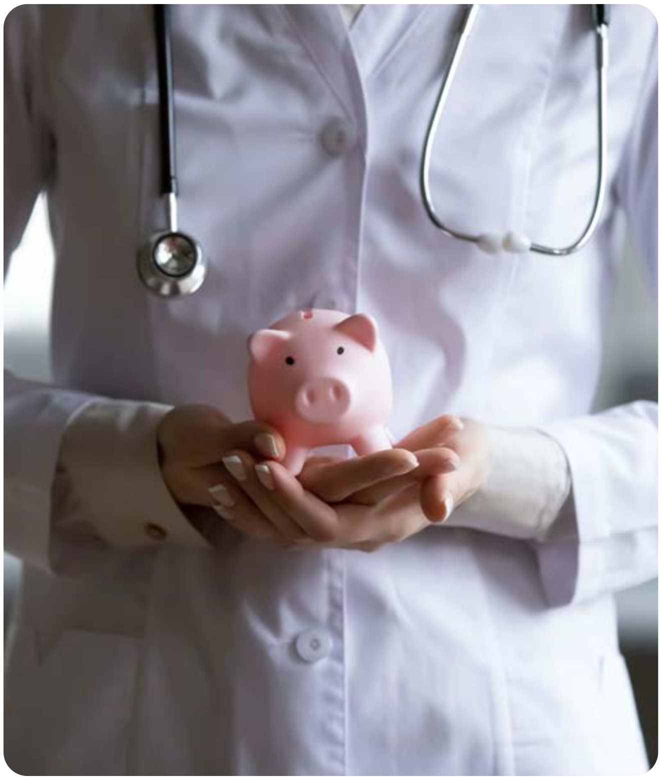

Research Impact

Scientific Discovery

Improving the state of readiness for genomic medicine is a necessary starting point for furthering our understanding the origin and natural history of disease. Scientific discovery in the realm of rare disease is already being bolstered by translational research funding programs, such as the Genome Canada-led "All for One" precision health partnership . The need to be ready for diagnostics has also been seen during the COVID-19 pandemic. Diagnostic technology played a critical role in monitoring and planning responses to the pandemic. Canadian research programs such as the University of Toronto's PRiME precision medicine initiative are working toward advancing genome-based solutions to improve Canada's response to monitoring and mitigating the impact of the pandemic.

While creating system readiness for genomic medicine can improve healthcare effectiveness and efficiency, Canadian healthcare systems are not positioned to "learn" from adopting commercially-driven applications of genomic medicine that do not further explore underlying theories of disease.[23,24] Advancing knowledge while creating opportunities for commercialization and economic growth requires a coordinated approach to scientific discovery that benefits from a nimble and flexible genomic medicine service as a basis for accelerating the translation of genomic data into patient care.[23]

As one commentator noted[25]:

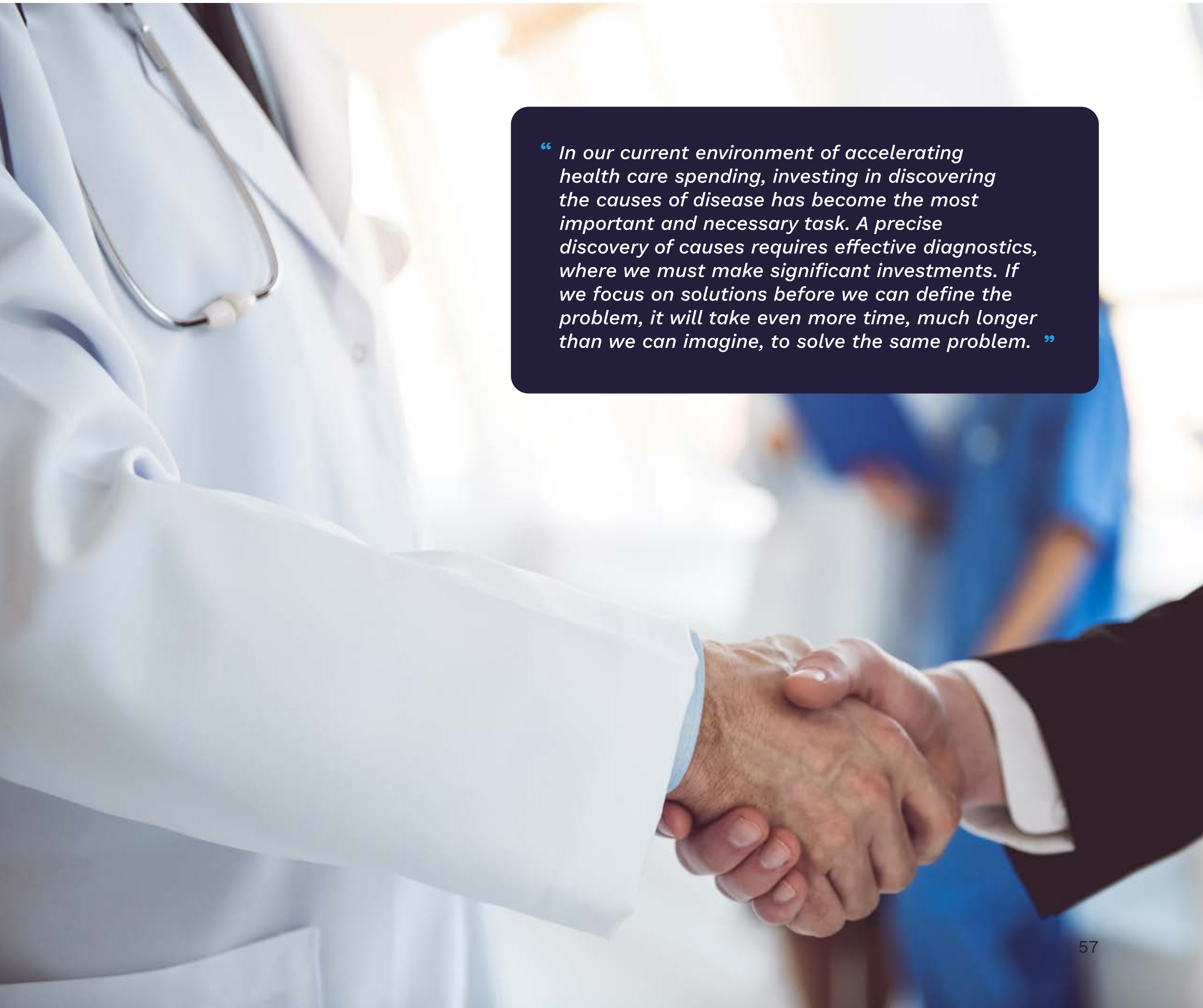

> *"In our current environment of accelerating health care spending, investing in discovering the causes of disease has become the most important and necessary task. A precise discovery of causes requires effective diagnostics, where we must make significant investments. If we focus on solutions before we can define the problem, it will take even more time, much longer than we can imagine, to solve the same problem."*

Table 2 *Estimated impact of genome-based testing in Canada (2015-2022)*

Type of Intervention	Intervention	Health	Patient & provider experience	Expenditure (Reductions)[1]	Value for Money	Reference
Prognosis (Prenatal / preconception)	**School-based screening for Tay-Sachs and Cystic Fibrosis**	• 22 more /1000 screened	-	$AUD 670M[2]	Reasonable (cost neutral)	Warren, 2005[1]
	**Non-invasive vs. traditional prenatal screening**	• 2.1 more trisomy / 10000 pregnancies	• 75% reduction in diagnostic tests	$35M	Questionable ($411K/case)	Ontario, 2019[2]
Diagnosis (screening or case finding)	**Lynch Syndrome (all new CRC)**	• 8 more / 1000 screened	• Family member status	$10.2M[3]	Questionable ($651K/ QALY)	CADTH, 2016[3]
	**Lynch Syndrome (select pop[4].)**	• 5 more / 1000 screened	• Family member status	$2.1M	Reasonable ($6K/QALY)	CADTH, 2016[3]
	**Targeted Exome Sequencing in Early-Onset Epilepsy**	• 10% greater yield	• Faster time to diagnosis	($500K)	Excellent (lower costs)	British Columbia, 2019[4]
	**Testing for familial hypercholesterol-emia**	• Reduced cardiovascular disease	-	($60M)	Excellent (lower costs)	Ontario, 2022[26]
	**Long QT Syndrome**	• Improved quality of life	-	$135M	Reasonable ($50K/QALY)	Perez, 2011[9]
	**Cascade testing with risk reducing surgery in *BRCA* carriers**	• 59 / 2800 cases prevented<br>• 25 deaths avoided	-	($194K)	Excellent (lower costs)	Hoskins, 2019[27]
	**Thyroid nodules of indeterminate cytology**	-	• Reduces unnecessary surgeries by 50%	$2.6M	Questionable (300K/QALY)	Ontario, 2022[17]
	**DPYD Genotyping in Patients with planned cancer treatment**	• Reduced toxicity from chemotherapy	-	($520K)	Excellent (lower costs)	Ontario, 2021[6]
	**Gene expression test to rule out melanoma**	-	• Fewer referrals<br>• Fewer biopsies	$1.8M	Questionable (not estimable)	Ontario, 2021 [10]
	**Noninvasive Fetal RhD Blood Group Genotyping (nonalloimmunized)**	• Avoid developmental problems	-	$7.7M	Reasonable ($154/QALY)	Ontario, 2020 [28]
	**Noninvasive Fetal RhD Blood Group Genotyping (alloimmunized)**	• Avoid developmental problems	-	($10.2M)	Excellent (lower costs)	Ontario, 2020 [28]
	**Unexplained Developmental Disabilities or Multiple Congenital Anomalies (controlled access)**	• 20% increased diagnostic yield	• More treatment modification	$8.7M	Questionable ($71K/treat-ment change)	Ontario, 2020 [5]

[1]Based on budget impact estimates. If specific to a Canadian province, then pro-rated to Canada
[2]Based on 500,000 grade 11-age children in Canada and $1339 additional costs per child
[3]Additional $382 per 26900 newly diagnosed cases
[4]Patients with MLH1 promoter hypermethylation, roughly 20% of all new cases

Cont'd: Diagnosis (screening or case finding)	**Unexplained Developmental Disabilities or Multiple Congenital Anomalies (1st tier testing, controlled)**	• 20% increased diagnostic yield	• More treatment modification	($7.7M)	Excellent (lower costs)	Ontario, 2020 [5]
	**Suspected sexually transmitted illness**	• Reduced symptoms, incl. pain	-	($323K)	Excellent (lower costs)	Quebec, 2022 [29]
	**Influenza diagnosis**	• Reduced morbidity from disease, medications	• Reduced patient transportation between facilities<br>• Reduced tests	($330K)	Excellent (lower costs}	Quebec, 2016 [30]
	**Pertussis diagnosis**	• Reduced morbidity from disease, medications	-	$600K	Questionable (not estimable)	Quebec, 2021 [31]
	**Multigene panels for muscle disease**	• Reduced harm from biopsy	-	($278K)	Excellent (lower costs)	Quebec, 2022 [32]
	**Stool analysis**	• Improved disease management	• Less referral<br>• Improved equity	($440K)	Excellent (lower costs)	Quebec, 2021 [18]
	**Premalignant Oral Dysplasia**	• Improved survivability	• Reduced clinic visits	NR	Excellent (lower costs)	British Columbia, 2016 [12]
Treatment	**Primary tumour in patients with cancer of unknown primary**	• Improved disease management	-	$35.2M	Reasonable ($44K/QALY)	Manitoba, 2016 [7]
	**EGFR t790 resistance mutation (liquid based, triage)**	• Improved response to medication, less toxicity	• Avoid biopsy<br>• Improve equity of access to testing	$3.3M	Questionable ($175K/ QALY)	Ontario, 2020 [8]
	**EGFR t790 resistance mutation (liquid based, alone)**	• Improved response to medication, less toxicity	• Avoid biopsy<br>• Improve equity of access to testing	($9.3M)	Excellent (lower costs)	Ontario, 2020 [8]
	**Early-stage, ER+, LN–, HER2– Breast Cancer**	• Reduction in chemotherapy	-	$4M	Reasonable ($20K/QALY)	Ontario, 2020 [33]
	**Major Depression**	• Improved response to medication	-	$27.3M	Questionable ($60K/QALY)	Ontario, 2021 [11]
**Policy response A:** Adopt only cost reducing interventions				**Cost reductions: $29.2M**		
**Policy response B**: Annual cost impact from implementation of cost reducing AND cost-effective (i.e., < $50K/QALY) interventions				**Annual costs: $17.6M**		

Clinical Trials

Improving Canada's state of readiness for genome-based testing will also be a pre-requisite for conducting clinical trials or quickly translating benefits from trials. In 2016 in Canada, there were an estimated 394 industry-sponsored trials providing care to ~6000 Canadian oncology patients.[34] At around the same time, worldwide, it was estimated that more than 90% of oncology trials involved targeted therapies.[19] In cancer, investigational treatment is often offered when other established options have been exhausted, even as part of provincial care protocols. Increasingly, in oncology, investigational trials are yielding substantive benefits for patients — a recent analysis of phase1 studies revealed targeted show median response rates across 58 biomarker-driven trials were 30% more on average (95%CI: 18.6-41.2) compared to 293 trials using a non-personalized strategy (0% [95%CI: 0-1.08])[35]. In this sense, readiness for genome-based medicine and clinical trials can offer potentially meaningful benefits for patients, beyond hope.

Future-Proofing a Healthcare Workforce

The era of genomic medicine and its potential to change medical decision making is already being reflected in the training of future healthcare professionals. Many countries have begun to make investments in workforce education.[36] For example, Health Education England (HEE launched a four-year £20 million Genomics Education Programme (GEP in 2014 to ensure their "1.2 million-strong NHS workforce has the knowledge, skills and experience to keep the UK at the heart of the genomics revolution in healthcare."[37] Australia similarly made creating a "skilled workforce that is literate in genomics" as part of their National strategic priorities.[38] These are initiatives are intended to ensure high quality care from the increasing integration of genomic medicine into mainstream medicine, an attempt to "future proof" a healthcare workforce.

In Canada, there is some indication that education at an undergraduate level is evolving.[39,40] This change of focus may have more immediate relevance in some medical specialties, such as pediatrics, internal medicine/oncology and obstetrics/gynecology. The lack of system readiness to meet the demands of a future workforce may be an issue, however, particularly in regions where workforce retention is an issue.

The Business Case for Change

Systemic readiness for genomic medicine holds the potential for obvious impacts for patients and broader society. While it cannot be predicted with a high level of accuracy to what extent these impacts will occur, it is clear that there are already gains to be made by more fully integrating genomic medicine into current processes governing care delivery and discovery. More certainly, any economic benefits will further increase as the operational costs of delivering tests decreases, and the costs of expensive care or resources avoided through testing increases. Investments in appropriate operational, infrastructure and broader healthcare conditions will also reduce ongoing change management costs from adapting to genome-based testing approaches on a one-off basis, or the costs of relying on out-of-country providers.

In healthcare, it is rare that investments in medical technology realize reductions in overall costs. However, when considering analyses applicable to the Canadian healthcare environment, it can quickly be seen that cost reductions are possible. The sample of analyses by independent researchers and HTA bodies identified (Table 2) suggest roughly $30M dollars of cost reductions could be achieved through the adoption of genome-based biomarker approaches of proven effectiveness and that have been identified as "cost-saving". If healthcare systems further adopted cost-effective interventions (i.e., those that are effective but incur overall additional costs then annual costs increase further by to a $17.6M annual increase.

While these expenditures and reductions are small relative to overall Canadian expenditure on healthcare, they are remarkable in an environment where almost all investments in healthcare technology lead to additional expenditure, and few healthcare innovations (other than reorganiza-tion of care organization and improving information communications infrastructure) actually lead to the routine avoidance of costs. The cost to establish most of the conditions of testing highlighted in Chapter 3 are also quite small, relative to annual healthcare expenditure. None of these immediate savings also capture longer-term benefits of scientific discovery , commercializing opportunities and future-proofing Canada's workforce.

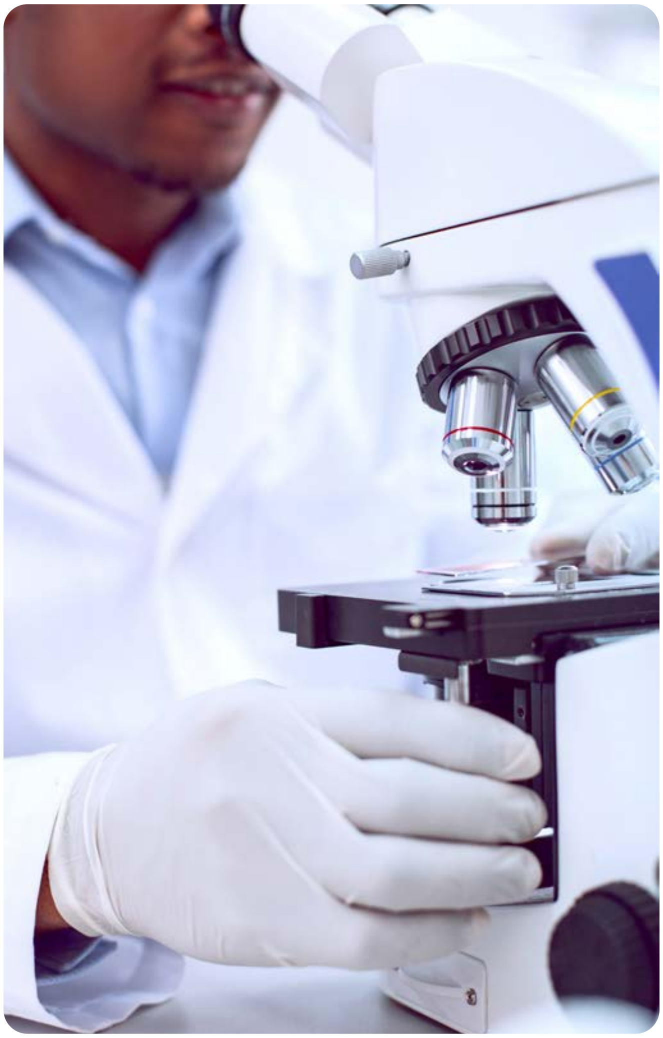

References

1. Warren E, Anderson R, Proos AL, et al. Cost-effectiveness of a school-based Tay-Sachs and cystic fibrosis genetic carrier screening program. *Genetics in Medicine* 2005;**7**:484–94. doi:10.1097/01.gim.0000178496.91670.3b
2. Health Quality Ontario. Noninvasive Prenatal Testing for Trisomies 21, 18, and 13, Sex Chromosome Aneuploidies, and Microdeletions: A Health Technology Assessment. *Ont Health Technol Assess Ser* 2019;**19**:1–166.
3. Assasi N, Blackhouse G, Campbell K, et al. DNA Mismatch Repair Deficiency umour Testing for Patients With Colorectal Cancer: A Health Technology Assessment. Ottawa (ON): : *Canadian Agency for Drugs and Technologies in Health 2016*. http://www.ncbi.nlm.nih.gov/books/NBK384771/ (accessed 2 Sep 2022).
4. Demos M, Guella I, DeGuzman C, et al. Diagnostic Yield and Treatment Impact of Targeted Exome Sequencing in Early-Onset Epilepsy. *Front Neurol* 2019;**10**:434. doi:10.3389/fneur.2019.00434
5. Ontario Health (Quality). Genome-Wide Sequencing for Unexplained Developmental Disabilities or Multiple Congenital Anomalies: A Health Technology Assessment. *Ont Health Technol Assess Ser* 2020;**20**:1–178.
6. Ontario Health (Quality). DPYD Genotyping in Patients Who Have Planned Cancer Treatment With Fluoropyrimidines: A Health Technology Assessment. *Ont Health Technol Assess Ser* 2021;**21**:1–186.
7. Hannouf MB, Winquist E, Mahmud SM, et al. Cost-effectiveness of using a gene expression profiling test to aid in identifying the primary tumour in patients with cancer of unknown primary. *Pharmacogenomics J* 2017;**17**:286–300. doi:10.1038/tpj.2015.94
8. Ontario Health (Quality). Cell-Free Circulating Tumour DNA Blood Testing to Detect EGFR T790M Mutation in People With Advanced Non-Small Cell Lung Cancer: A Health Technology Assessment. *Ont Health Technol Assess Ser* 2020;**20**:1–176.
9. Perez MV, Kumarasamy NA, Owens DK, et al. Cost-Effectiveness of Genetic Testing in Family Members of Patients With Long-QT Syndrome. *Circulation: Cardiovascular Quality and Outcomes* 2011;**4**:76–84. doi:10.1161/CIRCOUTCOMES.110.957365
10. Ontario Health (Quality). Pigmented Lesion Assay for Suspected Melanoma Lesions: A Health Technology Assessment. *Ont Health Technol Assess Ser* 2021;**21**:1–81.
11. Ontario Health (Quality). Multi-gene Pharmacogenomic Testing That Includes Decision-Support Tools to Guide Medication Selection for Major Depression: A Health Technology Assessment. *Ont Health Technol Assess Ser* 2021;**21**:1–214.
12. Cromwell I, Regier DA, Peacock SJ, et al. Cost-Effectiveness Analysis of Using Loss of Heterozygosity to Manage Premalignant Oral Dysplasia in British Columbia, Canada. *Oncologist* 2016;**21**:1099–106. doi:10.1634/theoncologist.2015-0433
13. Mighton C, Shickh S, Uleryk E, et al. Clinical and psychological outcomes of receiving a variant of uncertain significance from multigene panel testing or genomic sequencing: a systematic review and meta-analysis. *Genetics in Medicine* 2021;**23**:22–33. doi:10.1038/s41436-020-00957-2
14. Oliveri S, Ferrari F, Manfrinati A, et al. A Systematic Review of the Psychological Implications of Genetic Testing: A Comparative Analysis Among Cardiovascular, Neurodegenerative and Cancer Diseases. *Front Genet* 2018;**9**:624. doi:10.3389/fgene.2018.00624
15. Burke W, Parens E, Chung WK, et al. The Challenge of Genetic Variants of Uncertain Clinical Significance: A Narrative Revie . *Ann Intern Med* 2022;**175**:994–1000. doi:10.7326/M21-4109
16. Boycott K, Hartley T, Adam S, et al. The clinical application of genome-wide sequencing for monogenic diseases in Canada: Position Statement of the Canadian College of Medical Geneticists. *Journal of Medical Genetics* 2015;**52**:431–7. doi:10.1136/jmedgenet-2015-103144
17. Ontario Health (Quality). Molecular Testing for Thyroid Nodules of Indeterminate Cytology: A Health Technology Assessment. *Ont Health Technol Assess Ser* 2022;**22**:1–111.
18. INESSS. *INESSS*. http://www.inesss.qc.ca/ (accessed 2 Sep 2022).
19. Global-oncology-trends-2018.pdf. https://www.iqvia.com/-/media/iqvia/pdfs/institute-reports/global-oncology-trends-2018.pdf (accessed 2 Sep 2022).
20. Kasztura M, Richard A, Bempong N-E, et al. Cost-effectiveness of precision medicine: a scoping review. *Int J Public Health* 2019; **64**:1261–71. doi:10.1007/s00038-019-01298-x
21. Weymann D, Pataky R, Regier DA. Economic Evaluations of Next-Generation Precision Oncology: A Critical Review. *JCO Precision Oncology* 2018;1–23. doi:10.1200/PO.17.00311
22. Mateo J, Steuten L, Aftimos P, et al. Delivering precision oncology to patients with cancer. *Nat Med* 2022;**28**:658–65. doi:10.1038/s41591-022-01717-2
23. Hey SP, Kesselheim AS. Countering imprecision in precision medicine. *Science* 2016;**353**:448–9. doi:10.1126/science.aaf5101
24. Brock A, Huang S. Precision Oncology: Between Vaguely Right and Precisely Wrong. *Cancer Res* 2017;**77**:6473–9. doi:10.1158/0008-5472.CAN-17-0448
25. The Real Power of Precision: Redefining the Precision Medicine Initiative. *Harvard Health Policy Review*. https://issuu.com/harvardhealthpolicyreview/docs/2015_fall_precisionmed (accessed 15 Feb 2023).
26. Maguire T. Genetic Testing for Familial Hypercholesterolemia: *Health Technology Assessment*. 2022;**168**.
27. Hoskins P, Eccleston A, Hurry M, et al. Targeted surgical prevention of epithelial ovarian cancer is cost effective and saves money in BRCA mutation carrying family members of women with epithelial ovarian cancer. A Canadian model. *Gynecologic Oncology* 2019;**153**:87–91. doi:10.1016/j.ygyno.2019.01.018
28. Ontario Health (Quality). Noninvasive Fetal RhD Blood Group Genotyping: A Health Technology Assessment. *Ont Health Technol Assess Ser* 2020;**20**:1–160.
29. INESSS. *INESSS*. http://www.inesss.qc.ca/ (accessed 2 Sep 2022).
30. Martin G, Simon B. Évaluation économique du diagnostic moléculaire du virus influenza. Québec, QC, CAN: *Institut national d'excellence en santé et services sociaux* 2016.
31. Tchekanda E, Bélanger S. Avis: Modalités et trajectoire diagnostiques de la coqueluche. *Published Online First:* 2021. https://www.inesss.qc.ca/en/publications/publications/publication/modalites-et-trajectoire-diagnostiques-de-la-coqueluche.html (accessed 2 Sep 2022).
32. État des connaissances - Panel de la sclérose latérale amyotrophique avec ou sans démence frontotemporale par SNG. ;:**45**.
33. Ontario Health (Quality). Gene Expression Profiling ests for Early-Stage Invasive Breast Cancer: A Health Technology Assessment. *Ont Health Technol Assess Ser* 2020;**20**:1–234.

34. Tran DT, Akpinar I, Jacobs P. The Costs of Industry-Sponsored Drug Trials in Canada. *Pharmacoecon Open* 2020;**4**:353–9. doi:10.1007/s41669-019-0161-0
35. Schwaederle M, Zhao M, Lee JJ, et al. Association of Biomarker-Based Treatment Strategies With Response Rates and Progression-Free Survival in Refractory Malignant Neoplasms: A Meta-analysis. *JAMA Oncology* 2016;**2**:1452–9. doi:10.1001/jamaoncol.2016.2129
36. Stark Z, Dolman L, Manolio TA, et al. Integrating Genomics into Healthcare: A Global Responsibility. *Am J Hum Genet* 2019;**104**:13–20. doi:10.1016/j.ajhg.2018.11.014
37. About us. *Genomics Education Programme.* https://www.genomicseducation.hee.nhs.uk/about-us/ (accessed 6 Sep 2022).
38. National Health Genomics Policy Framework 2018-2021. *Canberra: Department of Health* 2017.
39. Thurston VC, Wales PS, Bell MA, et al. The current status of medical genetics instruction in US and Canadian medical schools. *Acad Med* 2007;**82**:441–5. doi:10.1097/ACM.0b013e31803e86c5
40. Plunkett-Rondeau J, Hyland K, Dasgupta S. Training future physicians in the era of genomic medicine: trends in undergraduate medical genetics education. *Genet Med* 2015;**17**:927–34. doi:10.1038/gim.2014.208

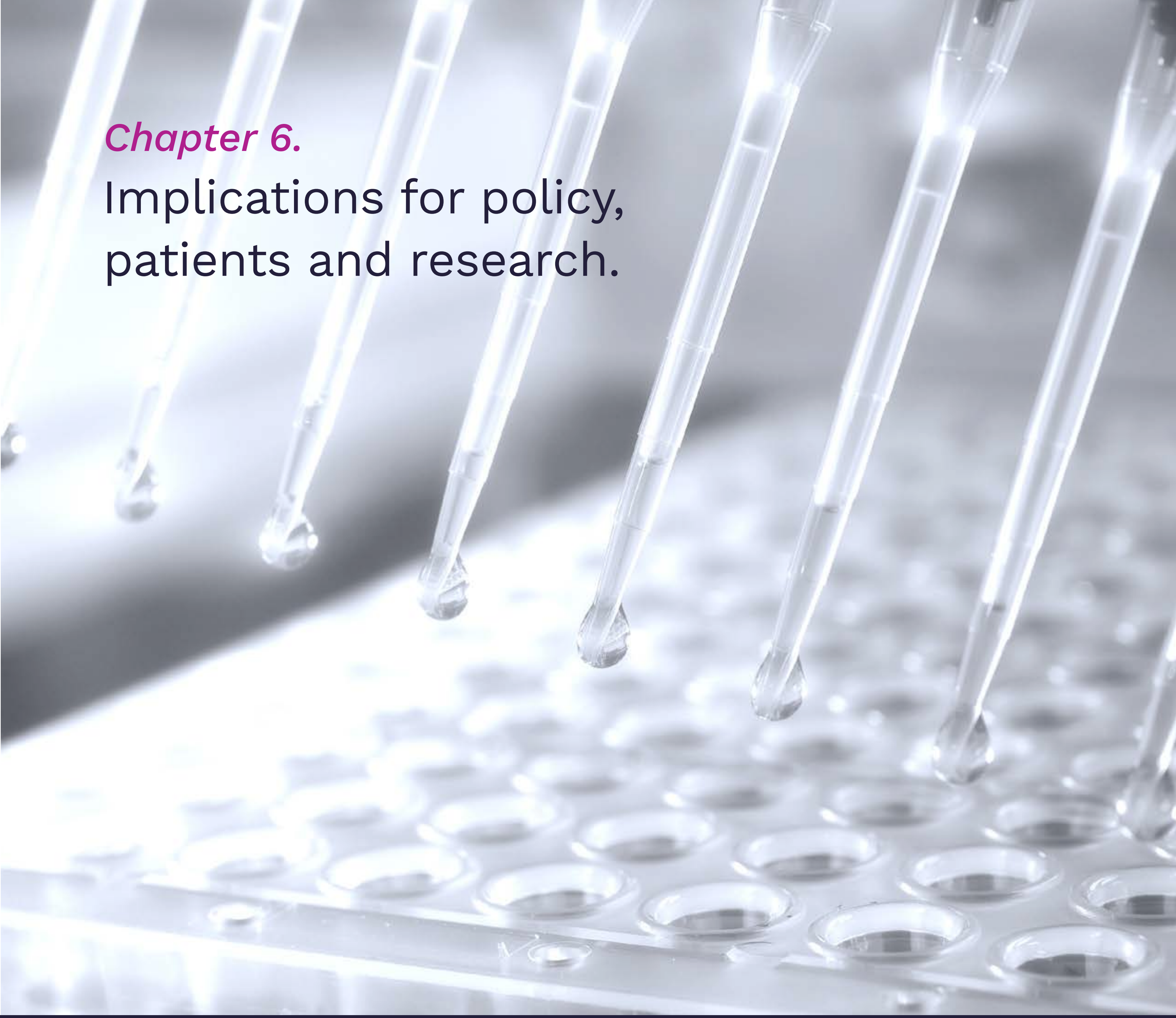

Chapter 6.

Implications for policy, patients and research.

CHAPTER SUMMARY

- Canada and its healthcare regions are in varying states of readiness for genomic medicine; given the future demand for testing we will likely see an increase in inequities across Canada due to inconsistent management and implementation of testing.
- Each province has different policy priorities in regards to creating an optimal environment for genomic medicine. Larger barriers to overcome include integration of laboratory information systems and changing financing approaches.
- While some provinces have created evaluative approaches for testing, these processes would better benefit by working more closely with commercial innovators and having transparent timelines, and evaluative criteria .
- There may be opportunities for larger scale provincial collaboration, however this will still require better developed provincial processes.

This Canada "State of Readiness" Progress Report highlights how ready Canada, through its provincial healthcare systems, is doing with regards to genome-based testing. More importantly, it suggests that provinces are in varying stages of evolution and that potential benefits to patients from genomic medicine will vary based on where they live. Currently, patients in Alberta will be more likely to benefit from a future of genomic medicine, while those in Ontario have a more uncertain future—even so, current efforts in Ontario are promising. Progress in jurisdictions such as Alberta and Quebec, however, suggest efforts to consolidate and improve testing infrastructure is a long, multi-year journey.

A key strength of some Canadian jurisdictions is the establishment of single service organizations dedicated to testing. These organizations allow for many of the necessary conditions required for testing, including establishment of necessary communities of practice, standards and resources required, oversight and resource planning, evaluation, and coordination of testing. While the creation of these organizations is a certain sign of progress, not all organizations in Canada have the same remit and some are still limited by conditions beyond their own control.

The British Columbia Provincial Laboratory Medicine Services (PLMS) organization, for example, while supported by legislation commencing in 2015, must still work within a BC governance model that allows individual health regions significant autonomy in decisions regarding information sharing and care delivery. The same is true in Ontario which must contend with a legacy of regionalization and hospital-level autonomy. In contrast, programs in Quebec and Alberta have benefitted from a higher level of centralized decision-making authority. Change in these provinces has been facilitated by vertical organizational integration and their ability to more readily influence care provision and the sharing of information.

One promising area for development in most provinces is the application of health technology assessment (HTA) to carefully consider the introduction (and obsolescence) of tests(1). HTA can serve as an excellent policy tool to support the management of health technologies where there are many new technologies to consider, a large potential for expenditure growth coupled with important potential impacts on patients, and considerable uncertainty regarding the value of technology. The growth of HTA provincially can be seen in the realm of formulary management, where provincial jurisdictions have created drug formularies informed by more sophisticated review processes, which have ultimately coalesced into a coordinated reimbursement review hosted by the Canadian Agency for Drugs and Technologies in Health.

While the emergence of HTA to address test decisions in each province is promising, HTA processes for testing in Canada are still generally lacking in key HTA principles(1,2) and good HTA practices(3). This includes processes that are consistent, timely, transparent, and responsive to and engaged with stakeholders(1,2). A more open *and* engaged approach to technology management may be foreign to laboratory leaders and Canadian health administrators who have, until now, managed laboratory technology using smaller internal processes. However, the interplay of social values reflected in genome-based testing, including the need for equitable healthcare and the potential for a large number of technology proposals and unmanageable expenditure growth necessitates a 21st century approach to HTA.

Robust HTA may be less feasible for smaller service organizations such as in Nova Scotia, who have fewer resources to commit to outward-facing evaluative processes. As with drugs, smaller provinces may benefit from a collective HTA approach. Quebec (so far) is the only service provider with a more transparent and timely HTA process, as it relies on INESSS, a long-established HTA organization recognized internationally(4).

Another feasible area for development across provinces is the development of navigational tools for patients and the public including referral guidelines, a test directory, eligibility criteria, tools/education for ordering genetic testing, and a care clinic directory. The lack of navigational tools was highlighted as a key gap (and policy priority in care delivery in Quebec, where processes to consider, onboard and coordinate the delivery of tests have been established through its Ministry-led Réseau Québécois de Diagnostic Moléculaire. The Ontario Provincial Genetics Program has similarly identified resources for care navigation as a priority work area. While many provinces, including Ontario, currently offer public test lists, genetic tests are often excluded or are have been inventoried separately. Navigational tools coupled with province-wide educational standards will be needed to effectively deliver genomic medicine into the future.

Some challenges identified across provinces may take more time to resolve. One key feature of any technology management process is the ability to quickly release funds or finance necessary technology once decisions to adopt have been made. This is particularly true when decisions to adopt (or change a testing paradigm have important consequences in terms of patient health and healthcare experience. While the vast majority of testing in provinces is delivered through contractual arrangements with community-based (private, for-profit laboratories), the responsibility for genetic an other specialized forms of testing has fallen to hospital laboratories, that are in turn funded through provincial block funding arrangements and annual budget cycles, along with public and private research grants, and private fundraising. These annual funding

> "*This State of Readiness Progress Report … suggests that potential benefits to patients from genomic medicine will vary based on where they live.*"

envelopes give hospitals the ability to quickly adopt and deliver new technology when they are seen as medically necessary and affordable. However, when the human resource, capital and operational costs of genetic testing are seen as too costly, laboratory leaders must rely on additional provincial funding, which can be slower than the speed of innovation.

To date, many provinces have used research funding, often from drug companies with targeted therapies requiring testing to make up for a shortfall in funding the development and delivery of tests. The problem with this approach is a variation of what has been called a Private Finance Initiative problem, where a "public sector party (e.g., a government department or a hospital trust invites private contractors in a tendering procedure to design, build, finance and operate public infrastructure according to the output specifications set out by the public authorities."(5) This can lead to substantive problems with governance and fiscal management where public sector actors are highly dependent on the private sector for the delivery of public services, and yet public actors remain accountable to the public at large. Health service delivery can be easily disrupted when the funding entity decides to cease funding.

Adding to this challenge is a need to change the funding formula for genetic testing. Charges for community-based testing have traditionally been operationalized through test schedules and based on historical costs of labour consumables and caps to limit excessive expenditure. Current per-test price constrained to test type, approach and patient type also does not consider efficiencies that could be realized with

changes in approach to testing type (e.g., multigene assay versus single gene approaches), approach (e.g., reflex testing or upfront testing versus ordered testing or sequential testing) or patient type (first line versus second line). Genetic tests are much more costly in terms of consumables and labour and require considerable upfront investment to implement, challenging these traditional per-test costing assumptions. While the funding formula for new tests was not publicized in most provinces, it appears only Quebec has made changes to its financing approach to account for some of this shortfall.

Ensuring a supply of testing that is responsive to patient and provider demand requires a sustainable funding solution that addresses both of these challenges. For provinces in Canada, this means reducing their reliance on the budgets of individual hospitals that provide these tests and, instead, directly providing operational funding through a dedicated provincial program or service organization. This funding must, in turn, consider the unique cost structure associated with genetic tests. In Canada, Alberta has adopted this approach'— providing funding based on appropriate forecasting to a single service organization, Alberta Precision Laboratories. Quebec has similarly created a Ministry-led program which allocates funds to individual hospitals, and is able to release funds when making decision to adopt new tests. Despite this nimbler financing arrangement in Alberta, they have continued to rely on private financing for new tests, when negotiating the adoption of new drugs through product listing agreements.

Specific policy priorities for each province are highlighted below:

Alberta

Alberta has established many of the necessary conditions (6) required to deliver genome-based testing to best benefit patients. It is leading Canada in its readiness for the coming era of genomic medicine. Alberta's key strengths are the use of a single service organization (APL) that provides oversight and resource planning coupled with an integrated laboratory information across province and some level of integration and exchange with commercial and public sector innovators. However, there are still opportunities to improve system readiness. Three key priorities are highlighted in Table 3.

A starting point is to create a 21st century process of evaluation and stakeholder engagement that is in line with good practices(1).Given the increasing demand for new tests and testing platforms, and the increasing impact on testing (and therapeutic innovators, there is a need for better processes of communication and engagement with the innovation community to understand potential demands for the future of testing as well as being part of a much more transparent process of accepting test proposals and conducting test reviews. Better engagement with stakeholders has the potential to improve understanding about what tests will be needed today and into the future. It will also give commercial innovators an opportunity to discuss the impact of testing decisions and policies and potentially create opportunities for partnerships or research investments that will lead to better outcomes and experiences in patient care.

While the APL has leveraged large scale communities of practice within the healthcare system such as the strategic clinical networks, there is still a hesitancy to engage with industry and other external stakeholders. Combined with an opaque evaluative process and funding formula, the result is a set of policies and practices in Alberta that do not fully support innovation as commercial and public innovators are left with little understanding of what types of testing technology are valued in Alberta, how they are valued, and ultimately what innovation is ultimately needed. A starting point for Alberta might be to involve industry and other stakeholders in its HTA process (as is done in Ontario and the UK) and additionally create networks or communities for engagement that involve non-clinical actors for the purposes of planning.

***Table 3** Key policy priorities to optimize genome-based testing in Alberta*

Evidence	Priority Action
High performing health systems require broad engagement of those impacted by testing. These include the patients, administrators, IT professionals, implementation and genome scientists, public and private sector innovators and others (scientists, legal and ethics experts, professional organizations, bioethicists, regulators)(7).	Expand opportunities for engagement with broader members of the healthcare / innovation community. This may be of particular use for healthcare planning.
More transparency around the test review process, timelines and criteria, will benefit a broader group of stakeholders. In doing so, it will also more closely adhere to current principles of technology assessment and deliberation (2,8) and improve perceptions of legitimacy for test adoption and create more opportunities for valuable innovation.	Improving the process of deliberation that surrounds the consideration and adoption of tests. Alberta's "one-test-at-a-time" approach will ultimately be unsustainable as more tests are introduced.
Unlike traditional tests, funding formulas for genetic testing must consider the need for additional human resources associated with development and profi-ciency testing (9) The current reliance on the private sector to fund test development may be counterproductive as priorities are influenced by who is paying, rather than unmet need, equity, or efficiency (10).	Improving the financing approach to include funding for test development and to account for capital infrastructure, human resources, and other associated costs of testing

British Columbia

British Columbia is leading necessary health system transformation through its development of a single Provincial program, the BC Provincial Laboratory Medicine Services (PLMS), which is creating many of the necessary operational conditions and underlying infrastructure required to optimize genetic testing (6). BC also has a single point of entry with explicit timelines for evaluation and coordination across service providers, a (somewhat) nimble financing approach and some integration of innovative testing. However, there are still some key opportunities for improvement. These are listed in Table 4.

One key priority for British Columbia is the integration of laboratory information systems, which will be a key for test development, interpretation, and clinical decision support (11,12) and for providing patients with equitable access to testing regardless of where they live in the province. BC's current care environment relies on a decentralized governance model which makes necessary integration of informatics systems (and necessary integration with other laboratory and clinical information) more difficult. This will invariably lead to challenges with the efficient and effective delivery of care, duplication of test ordering and the need for ongoing and high-level service coordination.

Decentralization creates additional challenges including difficulty creating resources for province-wide navigation and standards for education and training of care providers. Ultimately, the Provincial Health Services Authority has been mandated to "work collaboratively with the Ministry and regional health authorities to implement a cross-provincial plan and service coordination for pathology and laboratory medicine"(13) as well as some aspects of information technology and infrastructure and is expected to work on initiatives related to this in the coming years. Work to integrate laboratory information systems and care navigation in BC will likely need to be part of current efforts to create a province-wide shared clinical information system (called CST Cerner). Currently, three health regions (Vancouver Coastal; PHSA, and Providence Healthcare) are working together to establish the orders and results criteria for laboratory tests.

A second key challenge is to extend opportunities for engagement with the health system to a broader community. While the PLMS currently relies on consultation with clinical representatives through a committee structure, it could also consider inviting commercial innovators into its evaluative processes or striking a separate committee (i.e., such as an innovation committee) to represent stakeholders currently external to the healthcare system but whose involvement could potentially improve care delivery, consistent with best practices (1).

***Table 4** Key policy priorities to optimize genome-based testing in British Columbia*

Evidence	Priority Action
Informatics is essential for test development, interpretation, and clinical decision support (11,12). Ensuring adequate integration of test results into electronic health records will also provide a key resource for real-world monitoring, disease management, quality assessment and assurance, and financing (14)	Create a cross-regional integrated laboratory information system and a plan for integration into electronic health records.
High performing health systems require broad engagement of those impacted by testing. These include the patients, administrators, IT professionals, implementation and genome scientists, public and private sector innovators and others (scientists, legal and ethics experts, professional organizations, bioethicists, regulators)(7).	Expand opportunities for engagement with broader members of the healthcare / innovation community. This could include expanding discipline committee membership or creating new committees.
Effective delivery of genetic testing requires navigation tools for patients and the public including referral guidelines, a test directory, eligibility criteria, tools/education for ordering genetic testing, and a care clinic directory.(15)	Improving the processes of navigation for care providers and patients and develop standards for education and training.

Nova Scotia

Much of the infrastructure for Nova Scotia has been established through a dedicated program and coordination through its key teaching hospitals. Nova Scotia's smaller size relative to other Canadian provinces has also allowed it to have a nimbler financing approach and be an early adopter of investigational testing. Other key strengths in Nova Scotia include a dedicated program (Pathology and Laboratory Medicine Program - PLMP) that provides oversight and resource planning , high level of service coordination and the willingness to integrate innovative testing into its test lists. However, Nova Scotia still lacks an explicit onboarding process and evaluative evaluative approach — conditions (6) that will be required to best benefit patients in the future.

Some key actions for Nova Scotia are listed in Table 5. These gaps are similar to those seen in British Columbia and Alberta. However, given its population size, there may also be opportunities and incentives to coordinate evaluative processes and care with other Atlantic provinces. A current cross-Atlantic research initiative (called the Atlantic Cancer Consortium) is spearheading efforts to improve genetic testing in lung cancer across the region through a demonstration project. These types of initiatives may lead to opportunities to further coordinate other necessary services and reduce individual provinces' reliance on out-of-province testing.

***Table 5** Key policy priorities to optimize genome-based testing in Nova Scotia*

Evidence	Priority Action
Evaluation and adoption of testing must be responsive to innovation, transparent (8), timely and well connected to current investments in translational and discovery research as well as a community of care.(2)	Nova Scotia would benefit from a transparent evaluation process and a single-entry approach, supported by horizon scanning.
Informatics is essential for test development, interpretation, and clinical decision support (11,12). Ensuring adequate integration of test results into electronic health records will also provide a key resource for real-world monitoring, disease management, quality assessment and assurance, and financing (14)	A fully integrated laboratory information system connected to clinical health records would provide more benefit to patients and care providers and avoid unnecessary duplication and delay.
High performing health systems require broad engagement of those impacted by testing. These include the patients, administrators, IT professionals, implementation and genome scientists, public and private sector innovators and others (scientists, legal and ethics experts, professional organizations, bioethicists, regulators)(7).	There are opportunities to expand engagement with broader members of the healthcare / innovation community, particularly commercial innovators.

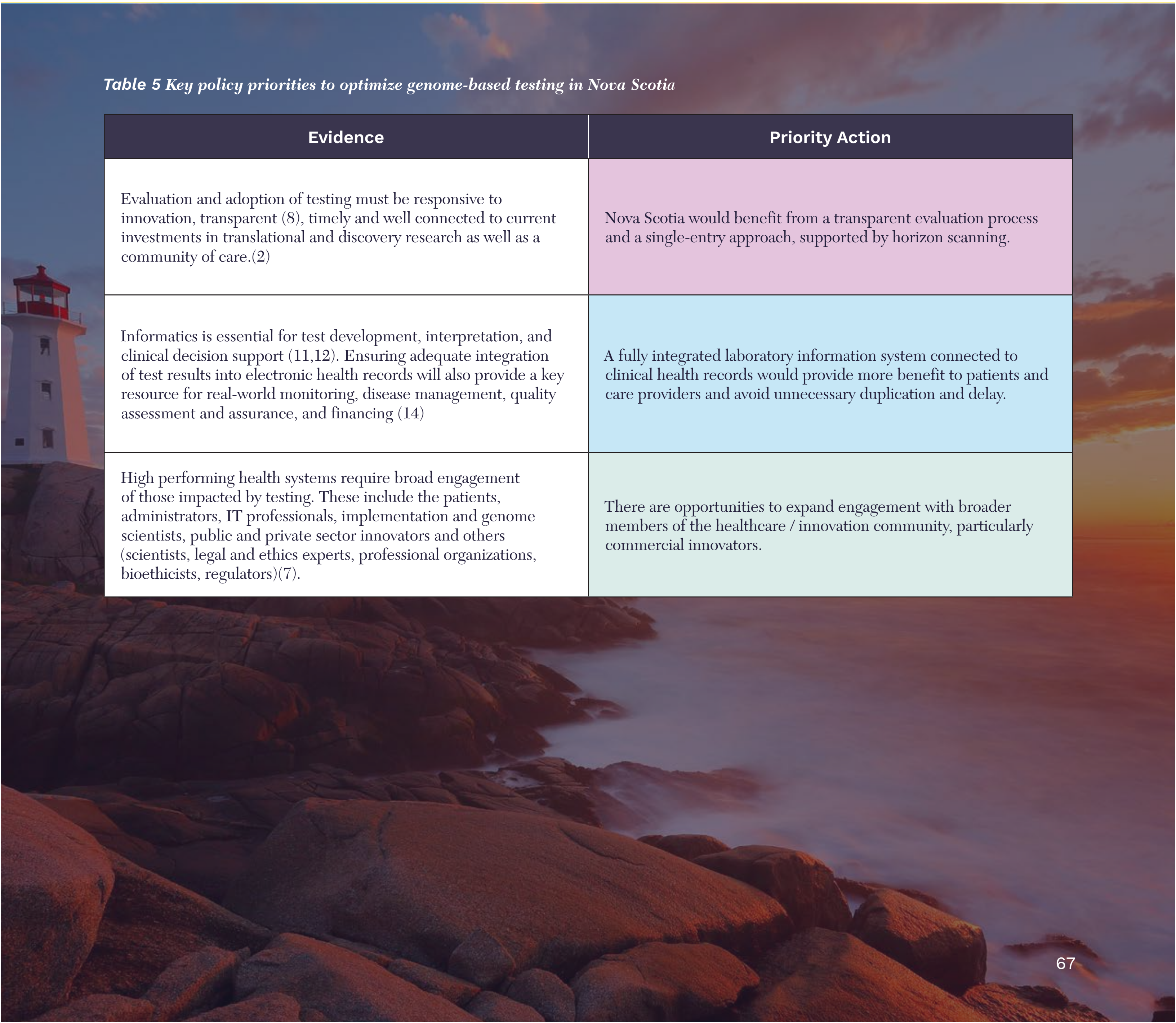

Ontario

In 2021, Ontario made the establishment of many of the necessary conditions (6) to deliver genome-based testing a health system priority. These efforts have come about quite late compared to other provinces but are timely, as Ontario is now able to capitalize on recent centralized health system reform which have created opportunities for improved coordination. It is early days for Ontario, and there are many significant opportunities based on evidence of best practices to improve readiness for genome-based testing.

Key priorities for Ontario are listed in Table 6. A feasible first step for Ontario is the consolidating the current patchwork of HTA processes and committees into a single process (16). The current framework means there is a lack of clarity for innovators who want to introduce new tests. Another potential option for change in Ontario may be to more clearly defining the terms of reference for individual HTA processes to have them work in lockstep. This is the approach taken in the UK, where diagnostics may be assessed through one of two HTA programs (Medical Technologies Evaluation Programme or the Diagnostics Assessment Programme) depending on whether additional costs are involved and other factors relating to complexity of testing.

Given the need test implementation that is responsive to clinical needs and healthcare quality, Ontario will also need to consider a more flexible funding model that allows for carry forward expenditures or the release of funds as needed (such as with the drug program) or simply a shift away from Ministry as decision-maker for individual tests and towards Ministry as steward over a genetic program that provides a service planning and oversight function for all testing along with a budget to allow for cross-healthcare system flexibilit . Like Quebec, it will also need to consider how to provide reliable funding for test development and the additional human resource needs associated with genetic testing.

***Table 6** Key policy priorities to optimize genome-based testing in Ontario*

Evidence	Priority Action
High functioning health systems must act as stewards, rather than decision-makers for individual purchasing.(17)	Ontario must shift away from the Ministry acting as a decision-maker for the funding of individual tests, and toward a system of Ministry as a steward. This will ensure expenditure, and care quality are driven by needs of the clinical community and avoid unnecessary patient delay.
Evaluation and adoption of testing must be responsive to innovation, transparent(8), timely and well connected to current investments in translational and discovery research as well as a community of care.(2)	Ontario has numerous, loosely connected systems of evaluation of testing. It must consolidate evaluation processes and adopt a single-entry approach, supported by horizon scanning.
Informatics is essential for test development, interpretation, and clinical decision support (11,12). Ensuring adequate integration of test results into electronic health records will also provide a key resource for real-world monitoring, disease management, quality assessment and assurance, and financing (14)	Ontario must create an integrated laboratory information system integrated with clinical health records to provide genetic testing that will most benefit patients and care providers while reducing unnecessary expenditure.

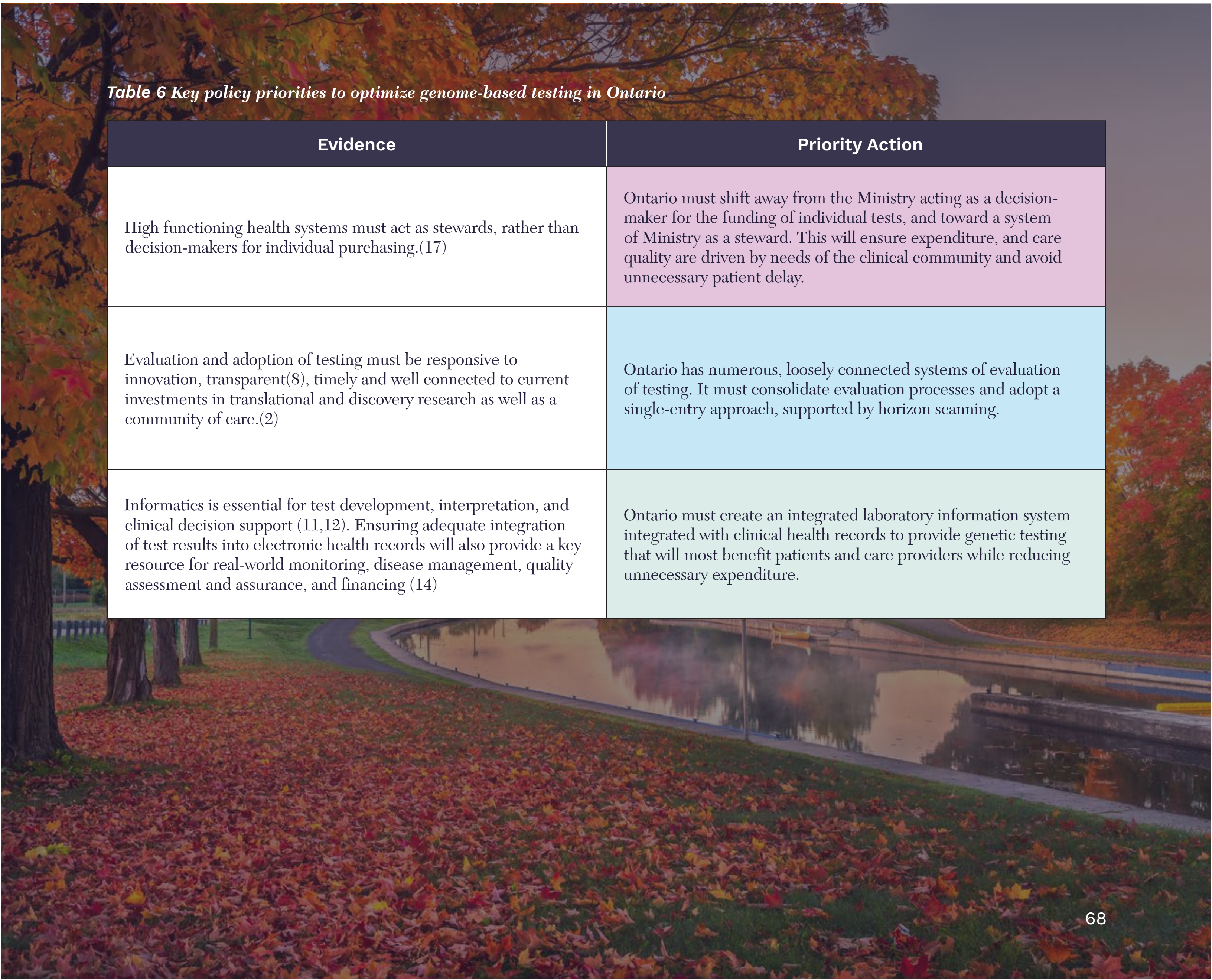

Quebec

Quebec has been a pioneer in laboratory governance reform and the use of a transparent and principled test evaluation process. It has established many of the necessary conditions (6) required to deliver genome-based testing to best benefit patients through its single service organization, the Direction de la Biovigilance et de la Biologie médicale (DBBM) The DBBM continues to provide oversight and resource planning across an integrated testing environment and hosts a single point of entry, along with INESSS, providing a transparent evaluation process for new tests. Another of Quebec's key strengths is its finance approach, with a more value-based funding approach that recognizes test development, along with the ability to release funds quickly after decisions are made.

Quebec has also more recently added a Bureau of Innovation (the Bureau de l'innovation en santé et en services sociaux) stemming from Quebec's 2017-2027 Life Sciences Strategy. Part of the Bureau's mandate is to accelerate innovation into the health sector. Coupled with the goal of fostering business that focuses on personalized medicine, Quebec may be prepared to improve its readiness for genome-based testing in the near future.

Some key opportunities to improve readiness are listed in Table 7.

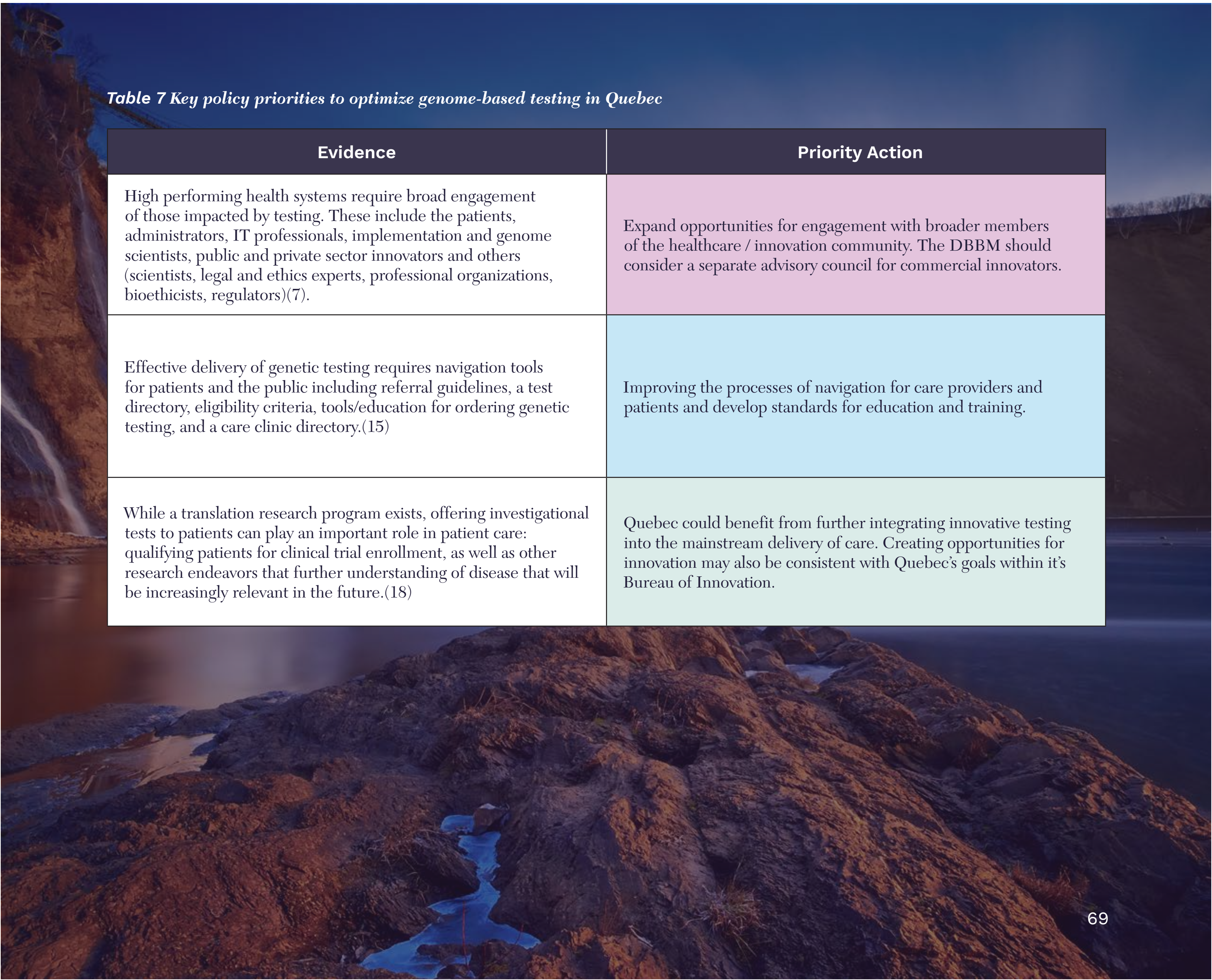

Table 7 Key policy priorities to optimize genome-based testing in Quebec

Evidence	Priority Action
High performing health systems require broad engagement of those impacted by testing. These include the patients, administrators, IT professionals, implementation and genome scientists, public and private sector innovators and others (scientists, legal and ethics experts, professional organizations, bioethicists, regulators)(7).	Expand opportunities for engagement with broader members of the healthcare / innovation community. The DBBM should consider a separate advisory council for commercial innovators.
Effective delivery of genetic testing requires navigation tools for patients and the public including referral guidelines, a test directory, eligibility criteria, tools/education for ordering genetic testing, and a care clinic directory.(15)	Improving the processes of navigation for care providers and patients and develop standards for education and training.
While a translation research program exists, offering investigational tests to patients can play an important role in patient care: qualifying patients for clinical trial enrollment, as well as other research endeavors that further understanding of disease that will be increasingly relevant in the future.(18)	Quebec could benefit from further integrating innovative testing into the mainstream delivery of care. Creating opportunities for innovation may also be consistent with Quebec's goals within it's Bureau of Innovation.

Concluding Remarks

General remarks

This progress report suggests that Canada's major healthcare regions are moving toward a state of readiness for genomic medicine, although using different approaches and at different rates. It highlights the many challenges that health systems face when they are required to quickly respond to a disruptive technology.

Even more so, this report highlights the differences in access to care that Canadian may face when they are served by individual health regions with different priorities and health care structures. Simply knowing a technology will promote the health and welfare of Canadians is not enough; we need to have responsive and resilient healthcare systems that are able to quickly shift priorities and be able to recognize and enable value innovation (19).

Implications for Canadian research

While this report is intended to motivate necessary change within healthcare systems, it also recognized policymakers may require further evidence to inform change. Given current gaps in Canada's genetic services infrastructure, there may be several areas of opportunity for future research funding. These include:

1. Research that supports the movement of innovation to practice including the alignment of genomic test development with the requirements for genomic test implementation. This research will likely require a high level of engagement from to patients, physicians and health systems to ensure its acceptance.
2. Development of an approach to data sharing – linking research data sharing with health system data sharing for genomic tests which will ultimately benefit patients.
3. Co-development of the ethical, legal, and social implications of new genomic testing – with a particular focus on providing the framework for developing navigational tools for those using the tests in the policy, health system and public/patient spheres.
4. Research that will build on current investments in rare-disease; as in cancer, genomic testing will increasingly play a role in rare disease and may provide helpful insights into how genomic testing should be implemented across other therapeutic areas.

Implications for Canadian patients

Genomic medicine has already demonstrated its incredible potential for enabling better and more timely care, and improving improved patient prognosis and quality of life. Already, patients have received more timely diagnosis for themselves. In cancer, genome-based testing has enabled access to more effective and less toxic therapies. For families, knowing what genetic or genomic mutations are responsible for their loved one's disease may also be potentially lifesaving and also allow for earlier (and less costly) interventions with better prognosis.

Although the pace of progress in Canada has been slow, patients, especially those with rare conditions and with poor prognosis, don't have time to spare. Making progress toward a state of readiness is not only a policy priority, it will affect the survival and quality of life of many Canadians.

The progress report also reveals some Canadians may currently benefit more based on where they live and who there care providers are. A recurring theme across patients with lived experience suggests patients in Canada worry that access to genetic services is determined not only by postal code, but by clinicians and how well they are informed about updates to the available tests.

The state of progress also reveals varying states of awareness about testing across the country. In addition to education standards, patients will require more access to genetic counselors who can help patients understand what test results mean and provide necessary psychosocial support. In some cases, test results are not immediately actionable. In others, actions may be taken through through clinical trials, special access programs, or patient support programs. Testing reveals that benefits to patients may fall outside of publicly funded healthcare services. It suggests the need for changing traditional models of care.

References

1. Garfield S, Polisena J, S. Spinner D, Postulka A,   . Lu C, Tiwana SK, et al. Health Technology Assessment for Molecular Diagnostics: Practices, Challenges, and Recommendations from the Medical Devices and Diagnostics Special Interest Group. *Value in Health.* 2016 Jul 1;**19**(5):577–87.
2. Drummond MF, Schwartz JS, Jönsson B, Luce BR, Neumann PJ, Siebert U, et al. Key principles for the improved conduct of health technology assessments for resource allocation decisions. *Int J Technol Assess Health Care.* 2008;**24**(3):244–58; discussion 362-368.
3. Kristensen FB, Husereau D, Huić M, Drummond M, Berger ML, Bond K, et al. Identifying the Need for Good Practices in Health Technology Assessment: Summary of the ISPOR HTA Council Working Group Report on Good Practices in HTA. *Value Health*. 2019**;22**(1):13–20.
4. Battista RN, Côté B, Hodge MJ, Husereau D. Health technology assessment in Canada. *Int J Technol Assess Health Care*. 2009 Jul;**25** Suppl 1:53–60.
5. Greer SL, Wismar M, Figueras J, European Observatory on Health Systems and Policies, editors. Strengthening health system governance: better policies, stronger performance. *Maidenhead, Berkshire: Open University Press*; 2016. **272** p. (European observatory on health systems and policies series).
6. Husereau D, Steuten L, Muthu V, Thomas DM, Spinner DS, Ivany C, et al. Effective and Efficient Delivery of Genome-Based   esting-What Conditions Are Necessary for Health System Readiness? *Healthcare*. 2022 Oct 19;**10**(10):2086.
7. Health C for D and R. Collaborative Communities: Addressing Health Care Challenges Together. FDA [Internet]. 2021 Oct 12 [cited 2022 Jun 23]; Available from: https://www.fda.gov/about-fda/cdrh-strategic-priorities-and-updates/collaborative-communities-addressing-health-care-challenges-together
8. Oortwijn W, Husereau D, Abelson J, Barasa E, Bayani DD, Santos VC, et al. Designing and Implementing Deliberative Processes for Health Technology Assessment: A Good Practices Report of a Joint HTAi/ISPOR Task Force. *Int J Technol Assess Health Care*. 2022 Jun 3;**38**(1):e37.
9. Christensen KD, Phillips KA, Green RC, Dukhovny D. Cost Analyses of Genomic Sequencing – Lessons Learned from the MedSeq Project. *Value Health*. 2018 Sep;**21**(9):1054–61.
10. Greer SL, Wismar M, Figueras J, European Observatory on Health Systems and Policies, editors. Strengthening health system governance: better policies, stronger performance. *Maidenhead, Berkshire, England: Open University Press;* 2016. **272** p. (European Observatory on Health Systems and Policies series).
11. Louie B, Mork P, Martin-Sanchez F, Halevy A, Tarczy-Hornoch P. Data integration and genomic medicine. *Journal of Biomedical Informatics*. 2007 Feb 1;**40**(1):5–16.
12. Shah S. Better bioinformatics will help labs manage genetic testing. *MLO Med Lab Obs.* 2AD;**49**(2):28, 31.
13. PHSA-2021-22-mandate-letter.pdf [Internet]. [cited 2022 Dec 9]. Available from: http://www.phsa.ca/about-site/Documents/PHSA-2021-22-mandate-letter.pdf
14. Warner JL, Jain SK, Levy MA. Integrating cancer genomic data into electronic health records. Genome Med. 2016 Oct 26;8(1):113.
15. Delikurt T, Williamson GR, Anastasiadou V, Skirton H. A systematic review of factors that act as barriers to patient referral to genetic services. *Eur J Hum Genet*. 2015 Jun;**23**(6):739–45.
16. Husereau D, Sullivan T, Feilotter HE, Gomes MM, Juergens R, Sheffield BS, et al. Optimizing the delivery of genetic and advanced diagnostic testing in the province of Ontario: challenges and implications for laboratory technology assessment and management in decentralized healthcare systems. *J Med Econ.* 2022;**25**(1):993–1004.
17. World Health Organization. Everybody's business : strengthening health systems to improve health outcomes : WHO's framework for action [Internet]. *Geneva: World Health Organization;* 2007. Available from: https://apps.who.int/iris/bitstream/handle/10665/43918/9789241596077_eng.pdf
18. Manolio TA, Rowley R, Williams MS, Roden D, Ginsburg GS, Bult C, et al. Opportunities, resources, and techniques for implementing genomics in clinical care. *The Lancet.* 2019 Aug 10;**394**(10197):511–20.
19. Faghfoury H, Guerin A. Ensuring timely genetic diagnosis in adults. CMAJ. 2023 Mar 20;195(11):E413–4.

Appendix A: Lab Organization Search

Conducted: February 25, 2022
Database(s): Ovid MEDLINE(R) ALL 1946 to February 25, 2022
Search Strategy:

#	Searches	Results
1	*diagnostic services/ or *clinical laboratory services/ or *genetic testing/ or *Molecular Diagnostic Techniques/	29363
2	exp *"Organization and Administration"/	768370
3	*"delivery of health care"/ or *"delivery of health care, integrated"/	74744
4	2 or 3	829847
5	1 and 4	1436
6	((laboratory or laboratories or ((genetic or molecular) adj (testing or diagnostic*))) adj2 (best practice* or plan* or service* or manag* or infrastructure or (deliver* adj2 (care or service*)) or organiz* or organis* or integrat* or administ* or harmonis* or harmoniz* or structur* or restructur*)).ti.	1715
7	5 or 6	3131
8	Diagnostic Services/og, st [Organization & Administration, Standards]	537
9	Clinical Laboratory Services/og, st [Organization & Administration, Standards]	464
10	*Genetic Testing/og, st [Organization & Administration, Standards]	1035
11	*Laboratories/og, sd	1703
12	or/8-11	3720
13	7 or 12	6426
14	(Animals/ or Models, Animal/ or Disease Models, Animal/) not Humans/	4930268
15	((animal or animals or canine* or dog or dogs or feline or hamster* or lamb or lambs or mice or monkey or monkeys or mouse or murine or pig or pigs or piglet* or porcine or primate* or rabbit* or rats or rat or rodent* or sheep* or veterinar*) not (human* or patient*)).ti,kf,jw.	2447417
16	14 or 15	5361308
17	13 not 16	6205
18	limit 17 to yr="2012 - Current"	2278

Google Advanced Scholar and Google Advanced:
Laboratory or lab organization OR integration OR planning OR services OR trends OR infrastructure "genetics" filetype:pd

Appendix B: Semi-Structured Interview Guide

Background

I have been asked by a consortium of companies (Amgen Canada Inc., AstraZeneca Canada, Eli Lilly Inc., GlaxoSmithKline Inc. (GSK Canada), Janssen Inc./J&J, Pfizer Canada Inc., Thermo Fisher Scientific Inc., and Roche Canada) to investigate what the current and future state of readiness for advanced (genome-based) diagnostic testing in Canada is and might become.

By advanced diagnostic testing we mean molecular testing (DNA testing such as sequencing, PCR and DNA microarray), cytogenetics (chromosome testing such as karyotyping and FISH) , and testing for metabolic products (protein testing through immunoassay or immunohistochemistry).

This work is to help all involved in advanced diagnostic testing in Canada to identify what can be done to make sure that Canadian health systems are prepared for the future of testing.

You have been identified as someone with expert knowledge in the area who could provide significant value to understanding the present and future of advanced diagnostic systems either nationally and internationally. As such, we would like to discuss the subject with you by phone for 45 to 60 minutes in a semi-structured interview.

This interview would cover your specific areas of expertise and the content developed through this interview would help inform the creation of a report that will be made available publicly.

Your contribution to this report will be acknowledged as a key informant, but there will be no comments specifically attributed to you. Notes from the interview will be shared with you after the call to ensure accuracy and to identify any areas of clarification required

Semi-structured interview guide

The interview begins with the interviewer stating the purpose of the interview, the topics that he wants to explore and the depth of response expected[1]

Purpose:

Interviewer: The purpose of today's interview is two-fold:

1. It will help identify current challenges with the uptake and routine delivery of advanced diagnostic testing
2. To explore what conditions are necessary and desirable for creating robust systems of advanced diagnostic testing (either in your region or generally)

Interviewer: I would like to cover a few topics today that will help answer the question concerning how advanced testing is being conducted today and what changes may be necessary to ensure its continued and effective delivery.

In particular I would like to explore your views on what approaches to the introduction and evaluation of tests, their development and validation, and financing of tests are needed as well as human resource and infrastructure required.

In each case, I will try to describe how much feedback is needed. However, I want to encourage you to speak freely in response to each question, even if you feel it doesn't directly address the question. We will have [time] for discussion.

Questions

1. Do you feel the current testing services offered are sufficient to keep up with the current and future demand for advanced testing?
2. What are the current challenges with the uptake and routine delivery of advanced diagnostic testing?
   - Do you have any cases that exemplify these challenges?
   - Do these challenges differ depending on whether testing is intended for diagnosis, therapeutic decisions, or hereditary testing?

3. What do you feel needs to change in order to keep up with current/future demand and address these challenges?
    - Who are the key decision makers, organizers and administrators of advanced testing that are currently involved?
    - Who else needs to be involved?
    - Are there any proposed changes currently?

4. Do you have any further thoughts on what needs to change to support a more nimble approach to the awareness, acceptance, and adoption of advanced testing?
5. Permission to Use Name, Interviewee demographics

References

1. Rubin HJ, Rubin IS. Qualitative Interviewing: The Art of Hearing Data. 2nd ed. Sage Publications, Inc; 2004.
